# Advantages and Limitations of Androgen Receptor-Based Methods for Detecting Anabolic Androgenic Steroid Abuse as Performance Enhancing Drugs

**DOI:** 10.1371/journal.pone.0151860

**Published:** 2016-03-21

**Authors:** Kathy Bailey, Tahmineh Yazdi, Umesh Masharani, Blake Tyrrell, Anthony Butch, Fred Schaufele

**Affiliations:** 1 Center for Reproductive Sciences, University of California San Francisco, San Francisco, California, United States of America; 2 Division of Endocrinology, University of California San Francisco, San Francisco, California, United States of America; 3 Department of Pathology and Laboratory Medicine, Geffen School of Medicine at UCLA, Los Angeles, California, United States of America; 4 Department of Obstetrics and Gynecology, University of California San Francisco, San Francisco, California, United States of America; Baylor College of Medicine, UNITED STATES

## Abstract

Testosterone (T) and related androgens are performance enhancing drugs (PEDs) abused by some athletes to gain competitive advantage. To monitor unauthorized androgen abuse, doping control programs use mass spectrometry (MS) to detect androgens, synthetic anabolic-androgenic steroids (AASs) and their metabolites in an athlete’s urine. AASs of unknown composition will not be detected by these procedures. Since AASs achieve their anabolic effects by activating the Androgen Receptor (AR), cell-based bioassays that measure the effect of a urine sample on AR activity are under investigation as complementary, pan-androgen detection methods. We evaluated an AR BioAssay as a monitor for androgen activity in urine pre-treated with glucuronidase, which releases T from the inactive T-glucuronide that predominates in urine. AR BioAssay activity levels were expressed as ‘T-equivalent’ concentrations by comparison to a T dose response curve. The T-equivalent concentrations of androgens in the urine of hypogonadal participants supplemented with T (in whom all androgenic activity should arise from T) were quantitatively identical to the T measurements conducted by MS at the UCLA Olympic Analytical Laboratory (0.96 ± 0.22). All 17 AASs studied were active in the AR BioAssay; other steroids were inactive. 12 metabolites of 10 commonly abused AASs, which are used for MS monitoring of AAS doping because of their prolonged presence in urine, had reduced or no AR BioAssay activity. Thus, the AR BioAssay can accurately and inexpensively monitor T, but its ability to monitor urinary AASs will be limited to a period immediately following doping in which the active AASs remain intact.

## Introduction

The abuse of unapproved PEDs remains a barrier to fair athletic competition [1–4]. For elite athletes, PED abuse is currently monitored by unannounced testing [5, 6]. The effectiveness of random sampling as a deterrent to PED abuse relies on the degree to which a susceptible athlete considers that the risk of being banned from competition outweighs the personal or monetary rewards that undetected doping may bring [6].

The abuse of PEDs, particularly androgens, is common also amongst non-elite athletes and by those in the general population seeking to improve body image [7, 8]. Annual surveys sponsored by the United States National Institute on Drug Abuse suggest that ~0.5% of 19–30 year olds in the US use AASs or the precursor to T, androstenedione, outside the care of a physician in any year [9]. Rates are higher in youths: in 2013, 2.9% of US grade 12 males self-reported androgen abuse over the prior twelve months [10]. That rate is similar to the androgen abuse rates reported in other western countries [11]. Although the current rate of androgen abuse amongst US grade 12 males is down substantially from a peak of 8% in the early 2000s [10, 12], AAS abuse by adolescents and adults remains troubling [13] and unmonitored.

Most methods of androgen monitoring rely on detecting, by MS, the chemical signature of T and known AAS within the urine of the athlete [14–17]. Since chemical assessment relies on a prior knowledge of the AASs, novel methods are currently under consideration that detect AAS activity in an athlete’s bodily fluids, even if the AAS is unknown [5, 18–20]. The list of androgens that could be used for doping continues to rise as pharmaceutical companies develop ‘selective androgen receptor modulators’ with androgen-like effects on the muscle but that lack the less desirable androgenic effects on other tissues [21–23]. Detection methods that survey for anabolic androgen activity, regardless of the chemical nature of the compound, therefore would find applications in both the clinic and in PED monitoring.

For pharmacologic research purposes, androgenic activity has long been assessed by the long-term androgenic and anabolic alterations in tissues of animal models injected with purified compounds [24]. However, applying these animal models to PED abuse monitoring would be impractical since insufficient androgen could be extracted from the athlete’s bodily fluids and because the assays would be costly, insensitive, time-consuming and an unnecessary use of animals. By contrast, miniaturized AR BioAssays, which are widely used for high throughput drug development studies [25–30], consist of cells engineered to permit easy, comparatively immediate quantification of a reporter of androgen-regulated AR activity on tiny samples. Initial studies showed a good correlation of AR BioAssay activation by known androgens with the ability of the same compounds to effect anabolic and androgenic changes in orchidectomized rats [31]. In theory, any known or unknown agonist of the AR, including those present in urine, will activate the AR BioAssay and could be useful for PED monitoring [5, 32].

Here, the efficacy of an AR BioAssay for detecting T and AAS abuse was examined in detail. AR BioAssay accuracy was evaluated by comparison of androgen measurement against MS measurement of T in urine samples and by detailed characterization of the AR BioAssay’s ability to detect AASs and the long-lived AAS metabolites most commonly monitored in urine by anti-doping laboratories. We discuss the types of PED abuse that are most amenable to detection by an AR BioAssay.

## Results

### AR BioAssay Design

**Fig 1:**
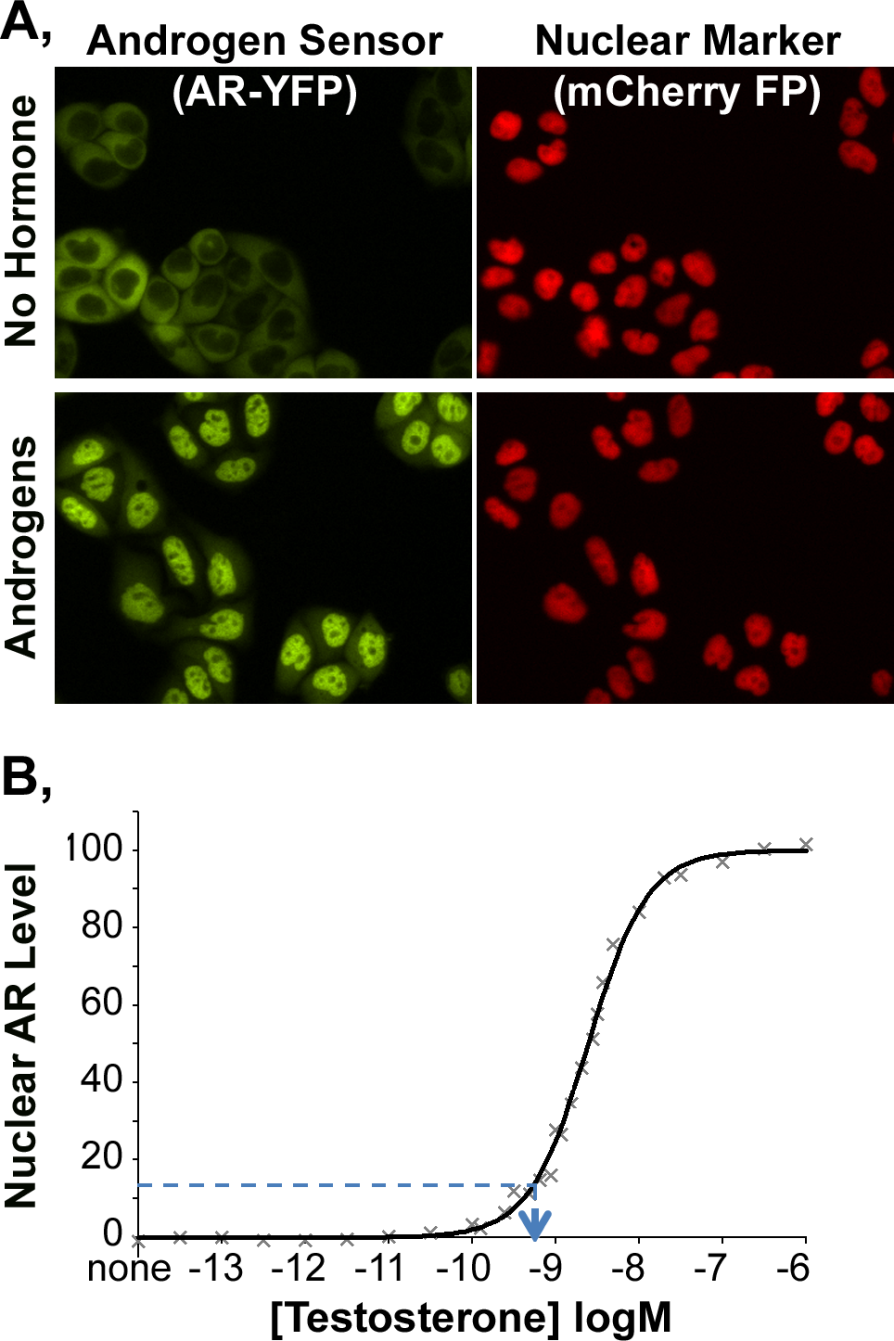
AR BioAssay for androgens. **A,** Fluorescence microscopy images of YFP-tagged AR and mCherry-NLS-mCherry nuclear marker expressed in the reporter cells grown in androgen-depleted cell culture media (upper panels). Incubation with an androgen redistributes the AR to the cell nucleus (lower panels). **B,** AR BioAssay standard curve: the concentration of T in the media dictates the level of ‘green’ fluorescence of YFP-tagged AR in image regions defined by red fluorescence emitted from mCherry FP-marked cell nuclei change. Maximum AR-YFP nuclear fluorescence is set as 100% and minimum is set as 0%. The nuclear AR level following incubation with a glucuronidase-treated urine sample defines the amounts of androgens present in the well (blue lines), which then is corrected for the urine dilution into the cell culture media to obtain the ‘T-equivalent’ concentration of urinary androgens present.

The AR BioAssay used here quantifies the androgen-mediated movement of a fluorescent protein (FP)-tagged human AR from the cytoplasm to the cell nucleus and was previously developed for high throughput quantification of AR response to androgenic drugs [26, 29]. That AR BioAssay consists of a HeLa cell line that stably expresses the yellow FP (YFP)-tagged AR together with an mCherry-NLS-mCherry construct (where NLS is nuclear localization signal). The ‘red’ fluorescence from mCherry-NLS-mCherry identifies the locations of cell nuclei in a field collected by high throughput microscopy (Fig 1A, right panels). Collection of ‘green’ fluorescence from the same field (left panels) identifies the locations and amounts of YFP-tagged AR relative to those nuclei. In the absence of an androgen, the AR resides predominantly in the cytoplasm of each cell (Fig 1A, upper panels). Upon the addition of an androgen, the AR moves into the cell nucleus (lower panels).

The AR BioAssay is conducted by plating reporter cells into a 384-well dish and adding known amounts of the androgen testosterone (Fig 1B) or a urine sample with an unknown androgen level. Comparing the level of nuclear YFP in cells incubated with urine to the nuclear YFP levels generated by the T dose response curve (Fig 1B, dotted line) allows extrapolation of the total amounts of urine androgen, expressed as ‘T-equivalent’ concentrations. Details of the procedures, including protocols for subtracting the background fluorescence from the cell culture media and other sources, are provided in the Materials and Methods section.

### Measurement Validation of the AR BioAssay

The accuracy of AR BioAssay measurement first was examined with urine samples obtained from T-treated males, in whom almost all androgens are T. In those participants, comparison of the T-equivalent concentration of androgens measured by the AR BioAssay should be the same as the T concentrations measured on the same urine samples by the UCLA Olympic Analytical Laboratory.

Androgen levels in 21 urine samples provided by 6 T-supplemented males were determined by incubating 0.5 μl of urine with the AR BioAssay cell line grown in 40 μl total volume cell culture media; up to 2.0 μl urine/40 μl was tolerated by the cells. Since >98% of urinary T is excreted into urine as an inactive conjugate with glucuronic acid [33, 34], the urine samples were pretreated with recombinant glucuronidase to release intact active T prior to addition to the AR BioAssay. There was none-to-little AR BioAssay activation if the urine was not pre-treated with glucuronidase (not shown).

Nuclear YFP fluorescence was measured following overnight incubation with the AR BioAssay cell line. Nuclear translocation was complete within 5 hours [35]. Thus, the 18–20 hour incubation used in the current study was sufficient to achieve steady state translocation at the time of AR-YFP measurement. The averages of quadruplicate AR-YFP measurements for each urine sample were compared against a T-standard curve (see Materials and Methods) to establish the androgen concentration (in T-equivalents); that androgen concentration was multiplied by the 80-fold dilution of 0.5 μl urine in 40 μl cell culture media for comparison against the urinary T concentrations measured on the same sample by mass spectrometry (MS) at the UCLA Olympic Analytical Laboratory.

**Fig 2:**
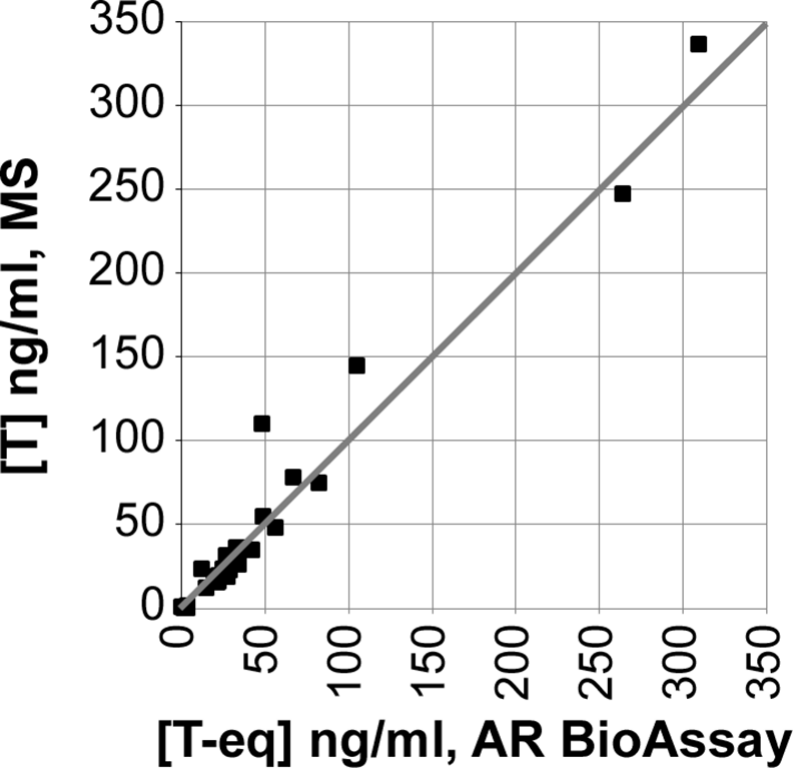
Equivalency of androgen (AR BioAssay) and T (MS) measurements in urine of T-treated patients. AR BioAssay reporter cells were grown in 39.5 μl androgen-depleted culture media and 0.5 μl of glucuronidase-treated urine. The AR-YFP fluorescence level activated by androgens in the urine was compared against the T standard curve to extrapolate androgen concentration in T-equivalent activity levels. That concentration was multiplied by the 80-fold dilution of the urine in the cell culture media. These urine androgen levels (x-axis) then were compared to T measurements conducted by MS on the same samples (y-axis). For T-supplemented individuals, linear regression would be hypothesized to show equivalency (slope = 1.0) of androgen and T levels.

The MS measurement of T and the AR BioAssay measurement of androgens in these T-supplemented males were identical (Fig 2; R^2^ = 0.98, slope = 1.04, y-intercept = 2.4). For those samples above the concentration considered to be measureable by the AR BioAssay (Fig 1, blue arrow; 8 ng/ml urinary T if the urine is diluted 80-fold in cell culture media), the concentration of T measured by the AR BioAssay was 0.96 ± 0.22 that measured by MS. The MS and AR BioAssay measurements for each urine sample were not statistically different (p = 0.20; by paired T-test). Thus the AR BioAssay provided measurements of T quantitatively identical to the well-validated MS T measurements conducted by a reputable laboratory.

### Tracking Androgen Levels Following T Injection

**Fig 3:**
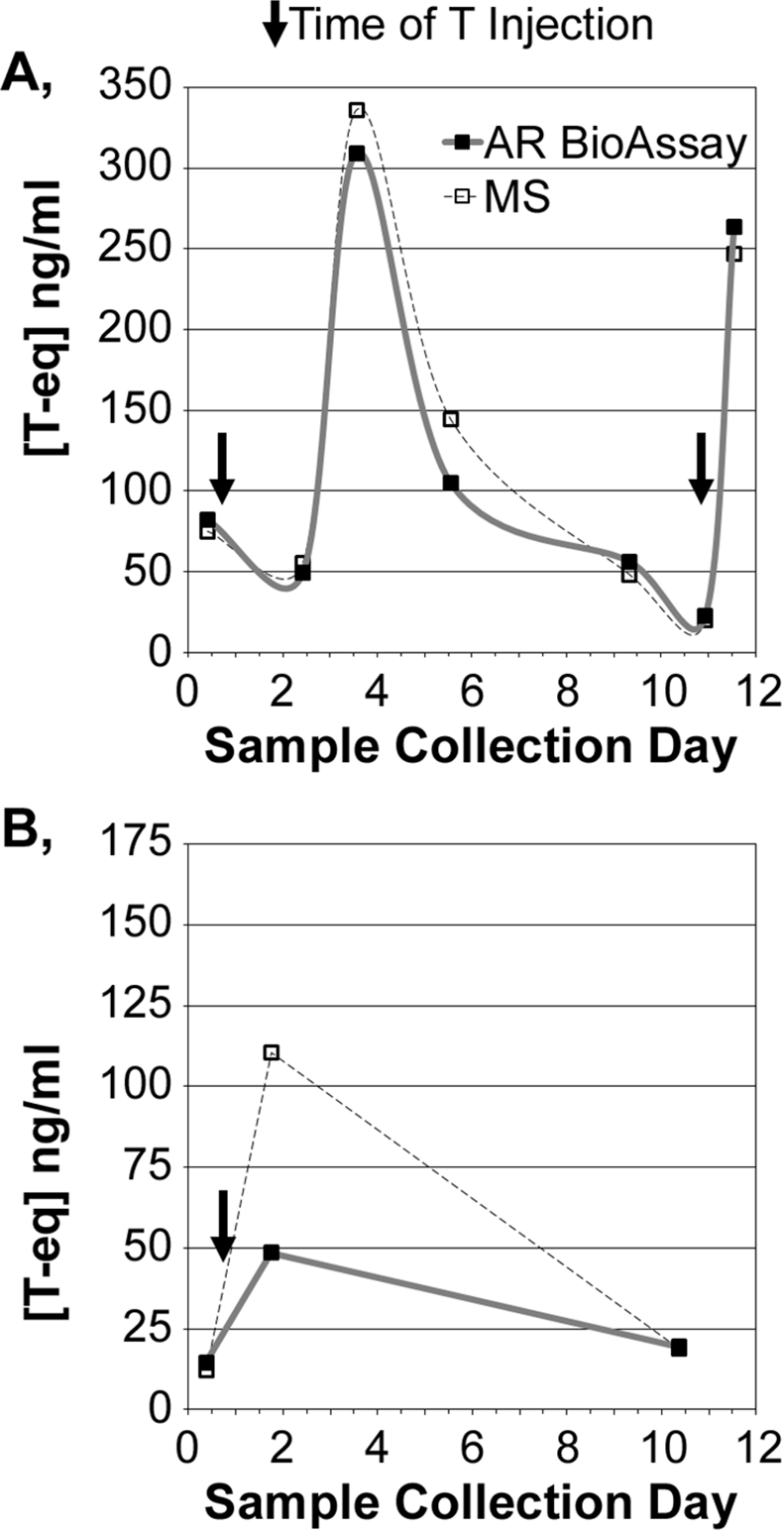
Rise and fall in urine androgens following injection with T. Androgen levels measured by AR BioAssay (solid line) and T levels measured by MS in glucuronidase-treated urine from two hypogonadal patients (**A** and **B**) pre- and post-T injection (arrows). Samples were collected and stored at home and returned at the next physician visit.

As further validation of the ability of the AR BioAssay to track androgen levels, two study participants (Fig 3A and 3B) provided urine samples before and after injection with T (arrows in Fig 3). The rise and decline in urinary T measurement were readily measured in both subjects by the AR BioAssay (solid line). Confirmatory measurements of T by MS also are shown (dotted line). One sample in Fig 3B showed the highest discrepancy between the AR BioAssay and MS measurements from all 39 urine samples tested, but the overall measurements obtained by AR BioAssay and MS were predominantly equivalent (Fig 2).

### Urinary Androgens

The AR BioAssay also should detect androgens other than T. In 18 urine samples from participants not receiving T therapy, AR BioAssay measurement of urinary androgen concentrations were 1.13 ± 0.23 the measurements of T detected by MS. Although this ratio of androgens:T was significantly higher than the 0.96 ± 0.22 measured in patients who were administered T (p = 0.03; by unpaired T-test), the findings indicate that the bulk of glucuronidase-released, active androgens naturally present in the urine is T.

### Androgen/EpiTestosterone Comparison

For PED testing, urinary T measurements are compared to the levels of epitestosterone (epiT), a stereoisomer of T. epiT-glucuronide and T-glucuronide are present at approximately equal concentrations in the urine of most individuals. Individuals receiving exogenous T experience an increase in the T-to-epiT ratio, which forms an initial assessment for doping with T [16, 36], although individual differences in T and epiT metabolism/disposition in the urine impact that assessment [33]. For the 11 urine samples from T-supplemented participants in our study with measurable androgens (>8 ng/ml T-equivalents), the androgen/epiT ratio, measured by AR BioAssay/MS respectively, was 19.8 ± 13.9 (range from 3.5 to 54.9), far above that measured in the 18 urine samples from participants not receiving exogenous T (1.15 ± 0.24; range from 0.53 to 1.41). For comparison, the T/EpiT ratio (both measured by MS) were 22.4 ± 16.8 (T-supplemented; range from 4.1 to 52.6) and 1.03 ± 0.17 (endogenous androgens only; range from 0.49 to 1.22). Thus, the AR BioAssay is capable of identifying increases in the T/EpiT ratio that occur after administration of T.

### Androgen Specificity of the AR BioAssay

**Fig 4:**
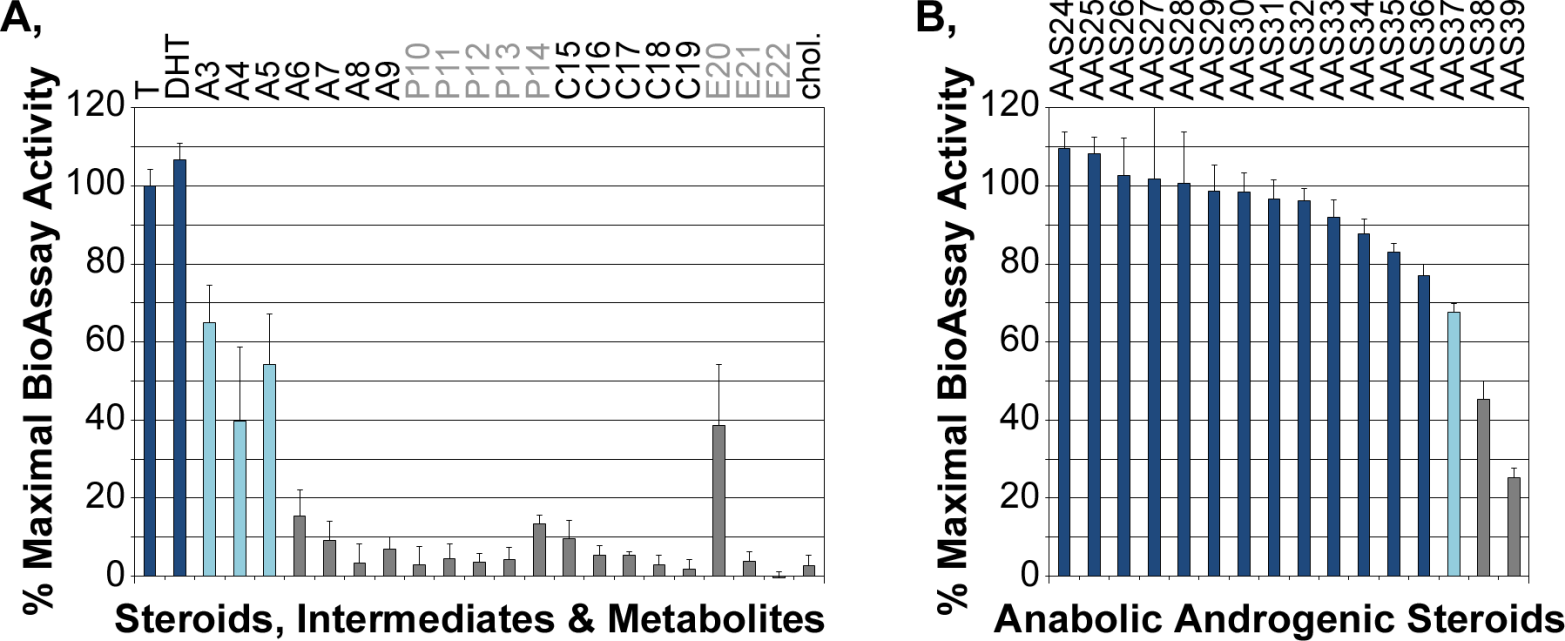
**Efficacy of A, natural steroids and B, AASs on the AR BioAssay.** Maximal AR BioAssay activity was determined from dose response curves (Fig 2). The compounds that achieved a maximal level of BioAssay activity at highest concentrations studied (10^-6^M) are shown in blue; steroids with efficacies comparable to testosterone (100% efficacy) are shown in dark blue. Those with lower efficacies are shown in light blue. Those compounds not achieving a maximum level of BioAssay activity are shown in grey. The mean ± sd for each compound were averaged from the number of independent dose response studies shown (see S1 Fig, which also includes the names and chemical structures of the compounds). In A, compounds are grouped according to global structural similarities to androgens (A), progestagens (P), corticosteroids (C) and estrogens (E).

**Fig 5:**
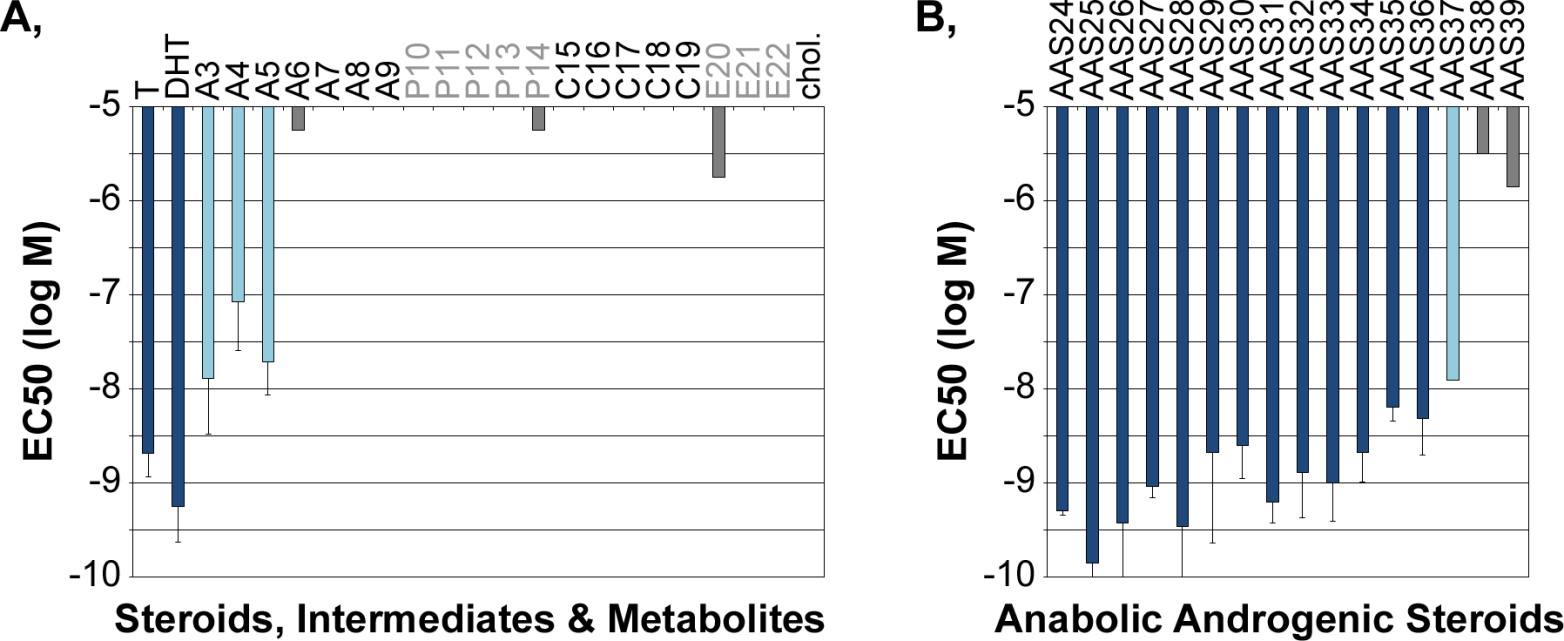
**Potency (Log EC50) of A, natural steroids and B, AASs in the AR BioAssay.** Potency can be established only for compounds that reached maximal activity at the highest concentrations investigated (10^-6^M). The more negative a Log EC50, the lower the concentration of compound required to reach half-maximal activity (i.e. higher potency). Many of the AAS’s have a higher potency than testosterone. Note that designer AAS’s, such as tetrahydrogestrinone (‘the clear’) are detected in this assay with an efficacy similar to DHT. See Fig 4 for description of symbols and procedures.

For the detection of PEDs beyond T, the AR BioAssay must respond to natural or synthetic androgenic compounds, and be non-responsive to chemically related, non-androgenic steroids. To examine the specificity of the AR BioAssay for androgens, a broad variety of steroids, androgens, AASs and their metabolites were characterized in dose response studies. The structures of the compounds investigated, their sources and the number of independent studies averaged in the data presented are provided in S1 Fig. The response summaries (Figs 4 and 5) are color-coded into three broad activity classes. Compounds that reached maximal AR BioAssay activity by concentrations of 10^-6^M or lower are shown as dark and light blue bars (Fig 4). Compounds that generated maximum AR BioAssay activity levels that were 75% or more the maximal level activated by T are depicted with dark blue bars whereas compounds that reached maximal activity levels less than 75% of the T-maximal activity are depicted with light blue bars. Grey represents ligands that did not achieve maximal activity at 10^-6^M; these are either completely inactive or have very modest activity at 10^-6^M. None of the compounds with no-to-low activity blocked AR BioAssay activation by 2x10^-9^M T (data not shown), indicating that those compounds are not antagonists competitively inhibiting the AR and therefore are not being recognized by the AR BioAssay.

### Efficacy of Natural Steroids and AASs in the AR BioAssay

Fig 4A summarizes the maximal AR activity level (“efficacy”) of a variety of natural steroids. The maximum level supported by T was set as 100%. The AR BioAssay was strongly activated by T and dihydrotestosterone (DHT). The immediate precursors (3β-androstanediol and androstenedione) and a metabolite (4-androstenediol) in the classical androgen biosynthesis pathway had reduced efficacy compared to T (Fig 4A, A3-A5). Other metabolites (5-androstenediol and epiT) and intermediates (dehydroepiandrosterone) had little to no activity (Fig 4A, A6-A8). Precursors to DHT synthesized in the alternative ‘back-door’ biosynthetic pathway [37–40] also showed no activity (Fig 4A, A9, P10).

All other natural steroids and steroid intermediates examined (Fig 4A, progestins: P11-P14; corticosteroids: C15-C19: estrogens: E20-E22; cholesterol) had no-to-low activity at the highest, non-physiologic concentrations, suggesting that the AR BioAssay has the desired specificity for active androgens. One natural steroid, estradiol, activated the AR only at a concentration 1000-fold above that of the peak pre-ovulatory serum concentration found in females and therefore would not physiologically impact AR activity. The androgen specificity of the AR BioAssay was confirmed by examining the efficacy of 16 known AASs commonly used as PEDs (Fig 4B). All 16 AASs strongly activated the AR BioAssay, most to the maximal level typical of T (dark blue). Thus, there appears to be a very high specificity of the AR BioAssay for detecting anabolic androgenic activities.

### Potency of Androgens in the AR BioAssay

Beyond defining the efficacy of different compounds in the AR BioAssay (Fig 4), the dose response curves also compare the compound doses required to activate the AR BioAssay. Fig 5A and 5B shows the potency (log of the concentration at which the AR BioAssay activity reached half maximum) for each steroid or AAS in which activity reached a maximum by 10^-6^M compound (dark and light blue bars) and for a limited number of poorly active compounds in which the maximum could be approximated (gray bars). Many of the AASs had a higher potency (more negative logEC50) than did the natural androgens T and DHT. Those with lowest efficacy (light blue bars) also tended to be those with lowest potency (logEC50 of -7.5 to -8.5, or 3 to 30 nM).

### AR BioAssay Detection of Androgen Byproducts

It may be possible to detect AAS abuse as an elevation in AR BioAssay activity in relationship to the T measured in the same urine sample. However, in the body most AASs are directly degraded prior to excretion. Thus, unlike the urinary T-glucuronide from which the intact T can be rescued by glucuronidase treatment, AR BioAssay efficacy for AAS detection rests with the extent to which AAS metabolites remain active in the urine. MS detection of the long-lived AAS byproducts constitutes the current method of detecting PED abuse [15].

**Fig 6:**
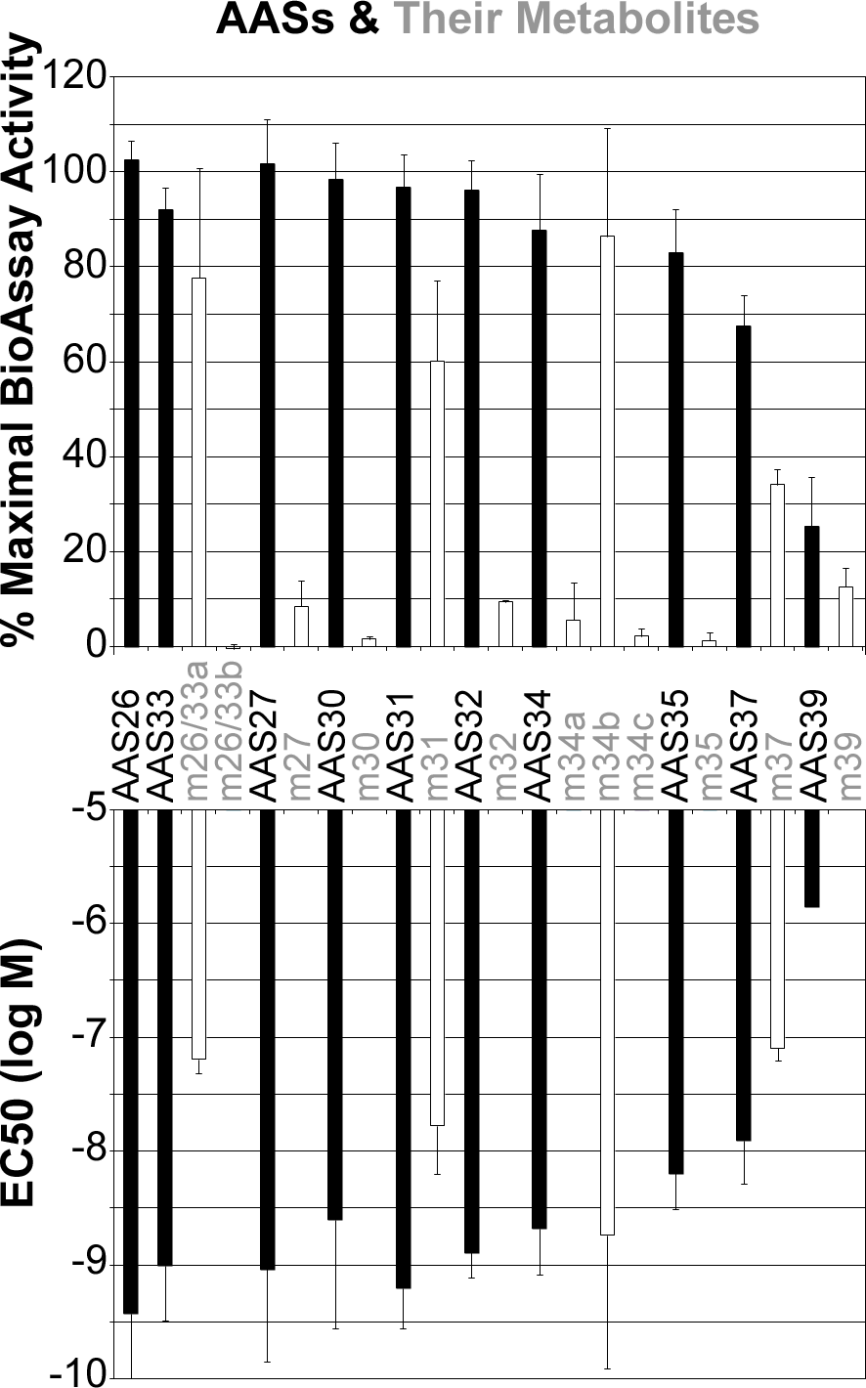
**A, Efficacy and B, Potency of AAS metabolites.** The metabolites (white bars; e.g. m27) are shown relative to the AASs from which they are metabolized in the body (black bars; e.g. AAS27). See Fig 4 for description of symbols and procedures.

Very little is known about the AR activity of the most prevalent AAS metabolites commonly surveyed by accredited anti-doping laboratories. We therefore examined the potency and efficacy of 12 known, long-lasting AAS metabolites generated from 10 common AASs (Fig 6A and 6B). The efficacies of two metabolites (Fig 6A, m26/33a,b) common to mestanolone (AAS26) and methyltestosterone (AAS33) were lowered or eliminated as previously indicated in a yeast-based, transcriptional reporter BioAssay [20]. Of the AAS metabolites, only 3’-hydroxy-stanozolol (m34b) had equivalent efficacy and potency to its AAS (stanozolol, AAS34). In general, the common AAS metabolites are considerably less active than the parental AAS and would be less able to be detected by an AR BioAssay.

The concentrations of androgens, AASs or their metabolites by the AR BioAssay that would have to be present in urine in order to be detected (Table 1) was calculated from the amount of compound needed to activate the AR BioAssay to a threshold detection level that was 10% of the maximal level of activation. The detection limit calculation assumes a dilution of 1 μl of urine into a 40 μl final volume of the BioAssay. Most of the high potency androgens (T, DHT) and AASs would be detected at low ng/ml concentrations. Even the most active long-lived AAS metabolite would need to be present at 10x the concentration of its parental AAS in order to be detected, which is not favorable for AR BioAssay detection.

**Table 1:** AR BioAssay detection limits for each compound in urine. Compounds showing any activity are in Bold. Detection limit is set to be AR nuclear fluorescence level of 10% the maximum of the T curve with a dilution of 1 μl of urine into 40 μl of total assay volume. Chemical structures and # of independent studies conducted for each compound are listed in S1 Fig.

Label in Figs 4–6	Steroid Class	Detection Limit ng/ml	Label in Figs 4–6	Steroid Class	Detection Limit ng/ml
**T**	**Androgen**	**4.7**	**AAS24**	**Androgen**	**1.2**
**DHT**	**Androgen**	**1.2**	**AAS25**	**Androgen**	**0.3**
**A3**	**Androgen**	**46.7**	**AAS26**	**Androgen**	**0.9**
**A4**	**Androgen**	**486—**	**m26/33a**	**metabolite**	**201—**
**A5**	**Androgen**	**83.0**	m26/33b	metabolite	>200,000—
**A6**	**Androgen**	**84,300—**	**AAS27**	**Androgen**	**2.2**
A7	Androgen	>200,000—	m27	metabolite	>200,000—
A8	Androgen	>200,000—	**AAS28**	**Androgen**	**0.8**
A9	Androgen	>200,000—	**AAS29**	**Androgen**	**5.2**
P10	Progestagen	>200,000—	**AAS30**	**Androgen**	**5.6**
P11	Progestagen	>200,000—	m30	metabolite	>200,000—
P12	Progestagen	>200,000—	**AAS31**	**Androgen**	**1.4**
P13	Progestagen	>200,000—	**m31**	**metabolite**	**60.2**
**P14**	**Progestagen**	105,000—	**AAS32**	**Androgen**	**3.4**
C15	Corticosteroid	>200,000—	m32	metabolite	>200,000—
C16	Corticosteroid	>200,000—	**AAS33**	**Androgen**	**2.6**
C17	Corticosteroid	>200,000—	**AAS34**	**Androgen**	**6.3**
C18	Corticosteroid	>200,000—	m34a	metabolite	>200,000—
C19	Corticosteroid	>200,000—	**m34b**	**metabolite**	**5.8**
**E20**	**Estrogen**	**10,000—**	m34c	metabolite	>200,000—
E21	Estrogen	>200,000—	**AAS35**	**Androgen**	**20.7**
E22	Estrogen	>200,000—	m35	metabolite	>200,000—
chol.	Cholesterol	>200,000—	**AAS36**	**Androgen**	**16.3**
			**AAS37**	**Androgen**	**49.6**
			**m37**	**metabolite**	**588—**
			**AAS38**	**Androgen**	**15,200—**
			**AAS39**	**Androgen**	**12,700—**
			m39	metabolite	>200,000—

## Discussion

An AR BioAssay, based upon the direct measurement of the nuclear levels of fluorescent protein-tagged AR, was extensively characterized against a wide variety of androgenic and non-androgenic compounds (Figs 4–6). The AR BioAssay showed outstanding specificity for intact androgens, either natural or synthetic. Other intermediates of steroid synthesis generally thought to be non-androgenic showed no or low activity in the AR BioAssay. Anti-androgens, commonly used in the clinical setting and unlikely to be abused as PEDs, showed the predicted ability to counteract androgen activation of the AR BioAssay (see S2 Fig, 5 nM DHT). However, prior studies in other stably engineered cell lines showed that some first and second-generation anti-androgens could, by themselves, increase the amounts of nuclear AR [28, 41]. Such AR BioAssay responses to anti-androgens would create an AR BioAssay response that would be misidentified as evidence for an androgenic function. The AR BioAssay used in this study was not activated by any first-, second- and third-generation anti-androgen (hydroxyflutamide, bicalutamide and MDV3100 or ARN-509; no DHT). It is therefore recommended that any BioAssay is comprehensively characterized against as many agents as possible to ascertain the confidence in which the activity measured is associated with the biological or clinical target activity. Overall, the pan-androgenic specificity of the current AR BioAssay suggests that it is strongly associated with active androgens.

The quantitative accuracy of the AR BioAssay for urine samples was validated in males undergoing T supplementation therapy (Figs 2 and 3). In prior studies from others examining urine from individuals who were not T-supplemented [32], an AR BioAssay based upon a transcriptional reporter of AR function also correlated with the measurement of endogenous androgens. In our studies of males not undergoing T therapy, the androgen levels measured in urine were only modestly higher than the MS-measured T concentrations. Thus, the AR BioAssay is an outstanding quantifier of androgen concentration, of which T appears to be the predominant glucuronidase-rescuable androgen in the urine.

The ability to track changes in androgen levels following T supplementation (Fig 3) suggests that longitudinal AR BioAssay tracking might identify a doping event. Indeed, longitudinal athlete biologic passport programs have been implemented to compare samples collected from elite athletes soon after doping against that individual’s prior personal measurements [42–44]. The passport represents an improvement over the prior practice of comparing measurements to the population average, which any individual may diverge from to generate false negative or false positive findings. The findings presented here suggest that the AR BioAssay would be amenable for passport analysis, at least for T supplementation, particularly if a parallel BioAssay were available to measure epiT (S3 Fig; mutant AR that binds epiT) as an internal control.

The motivation for using the AR BioAssay in PED testing is its theoretical ability to detect previously unknown urinary AASs [5, 18–20, 32]. That capability depends upon the retention in urine, or rescue from urine, of excreted functionally active androgens, AASs or particularly long-lasting AAS metabolites that enable detection of intermittent doping long after the doping event. However, only 1 of 12 long-lasting AAS metabolites known to be prevalent in urine following AAS doping was strongly active in the AR BioAssay (Fig 6). Thus, for almost all AASs, the AR BioAssay would detect abuse only if the urine sample is fortuitously collected soon after AAS doping.

The best method to assess by which an AR BioAssay detects unknown, functionally active AAS metabolites in urine samples is to conduct extensive studies on humans doped or not with each of the known AASs. A prior report of a prolonged elevation in urinary androgen levels detected by a yeast-based AR BioAssay two weeks after administration of methyl-testosterone to a volunteer suggested that some AASs may be detectable by an AR BioAssay [20]. Indeed, our dose response studies of the two primary known major metabolites of methyl-testosterone showed that one of them was active in the AR BioAssay (Fig 6A, compare AAS33 to m26/33a) albeit with a 65-fold poorer potency than methyl-testosterone (Fig 6B). However, in the prior study [20], MS analysis of those same, highly prevalent methyl-testosterone metabolites, conducted in parallel with the AR BioAssay measurement on the same urine samples, showed those metabolites to be eliminated rapidly, even though AR BioAssay activity was reported to persist. A persistence of other, currently unknown functionally active methyl-testosterone metabolites may account for that discrepancy between metabolite absence and the retention of AR BioAssay response. However, inspection of the AR BioAssay data provided with that isolated report also showed that androgen activity levels returned multiple times to baseline over the 14 day period investigated [20], which may be more consistent with the detection of a periodic fluctuation in endogenous urinary androgen concentrations than with detection of any unknown, long-lasting methyl-testosterone metabolites.

Overall, the data reported here indicates that the urinary metabolites of known AASs will be more reliably detected by existing MS procedures than by AR BioAssay methods, which implies that there is no substantive benefit for using the AR BioAssay to detect known AASs. Enthusiasm for applying the urinary AR BioAssay to detect AASs currently unknown to the anti-doping authorities, which was our primary motivation for the studies, also was dampened by our findings that most of the known, long-lasting AAS metabolites are functionally inactive (Fig 6). Thus, the AR BioAssay detects testosterone levels in urine with the accuracy and precision of MS (Fig 2) but the inactivity of almost all of the metabolites rapidly created upon AAS doping make it unlikely that a urinary AR BioAssay will provide a doping detection method that will substantively complement the deficient detection of unknown AASs by the well-established urinary AAS detection methods currently used by anti-doping authorities.

## Materials and Methods

### AR BioAssay Cell Line

HeLa cells stably co-expressing an mCherry-NLS-mCherry nuclear marker (linked to a blasticidin-resistance expression cassette) and a CFP-AR-YFP reporter (human AR dual-tagged at its amino and carboxy termini with CFP and YFP, respectively; linked to a G418-resistance expression cassette) were previously described [26, 45, 46]. HeLa cells do not contain endogenous androgen receptors that could compete with the CFP-AR-YFP reporter for the androgens available in the biologic fluid and thereby diminish assay sensitivity. Many cell types that have endogenous receptors, particularly those derived from prostate tumors, also have mutations within the AR that alter responses to some androgens, other steroids and anti-androgens [26, 28, 35, 41, 47–49] and which could interact with the CFP-AR-YFP reporter and alter its response to non-androgenic compounds. One should always be cautious that cell line-specific differences in the types and levels of coregulatory factors will affect the types and levels of any function-based BioAssay used for any application. Although characterization of the AR BioAssay against 55 steroids, steroid precursors and metabolites, AASs, AAS metabolites and anti-androgens (Figs 4–6 and S2) suggest that the nuclear localization response in the HeLa cellular background is globally associated with some aspect of anabolic androgenic activity, any natural or synthetic AR-regulating compound that generates a response affected by coregulatory pathways missing, underexpressed or overexpressed in the HeLa cell background may be disproportionately detected.

The reporter cells were maintained in DMEM-H21 cell culture media (University of California San Francisco (UCSF) Cell Culture Facility) supplemented with 5% fetal bovine serum (FBS, various sources), 2 mM glutamine (UCSF Cell Culture Facility), 700 μg/ml G418 and 10 μg/ml blasticidin. Cell lines were maintained in culture for less than 15 passages before new vials were thawed and propagated.

Prior to the start of an AR BioAssay measurement, the plated cells were androgen-depleted by washing three times with phosphate buffered saline then maintained for 20 or more hours in ‘androgen-free’ media consisting of a 50:50 mixture of phenol-red-free DME-H21/Ham’s F-12 media (Mediatech Inc 90-090PB, Herndon, VA, USA) supplemented with 5% fetal calf serum charcoal/dextran-stripped of steroids (HyClone SH30068.03, Thermo Scientific, Logan, UT, USA) and 0.4 mM glutamine. Cells then were seeded at 1500 cells per 30 μl of androgen-free media in each well of a 384-well optical imaging plate (Greiner Bio-One 781091, Frickenhausen, Germany).

### Preparation and Addition of Urine to AR BioAssay

One day after seeding, each well was treated with a urine sample diluted in media and treated with glucuronidase as follows: 30 μl of urine was incubated at 37°C for 60 minutes in 300 μl total volume with 270 μl of phenol-red-free DME-H21/Ham’s F-12 media containing 100U of recombinant β-glucuronidase (Sigma G4820, Sigma-Aldrich, St. Louis, MO, USA). Following incubation, two aliquots of 140 μl each of the mixture were then diluted 2-fold with 140 μl of phenol-red-free DME-H21/Ham’s F-12 media into which had been diluted either with a, T to a 8x10^-7^M final concentration from a 10^-3^M T stock in ethanol or b, the same volume the ethanol (0.08% in 140 ml). For each mixture, 10 μl was added to the 30 μl of cells/media in quadruplicate wells for final concentrations per well of a, 0.5 μl urine with 10^-7^M T/0.01% ethanol or b, 0.5 μl urine with 0.01% ethanol. The purpose for collecting parallel wells treated with urine and a high concentration of T is described under the section ‘Correcting Urine Amplification of YFP Fluorescence’.

No AR BioAssay activity was detected when urine samples were sham-incubated without glucuronidase (not shown) consistent with the majority of T being glucuronidated in urine. Pilot studies with differing levels of glucuronidase (not shown) were conducted to ensure that the amount of enzyme used was sufficient to fully release active T from the T-glucuronide with up to 2 μl urine/well. There was no visual impact on cell health with the amounts of urine and glucuronidase used in this study. Glucuronidase-containing crude extracts from the snail *Helix pomatia* also were examined as a source of glucuronidase but showed some batch variations that had modest toxicity on the reporter cells at the highest levels.

### Quantification of Nuclear AR (YFP) Fluorescence

One day following the addition of urine, green fluorescence from the YFP-tagged AR and red fluorescence from the mCherry-linked nuclear marker were collected on an IXMicro High Throughput Microscope (Molecular Devices Corp., Sunnyvale, CA, USA) using filter sets previously described [29] and a 10x objective that our prior studies had shown to maximize the numbers of cells collected in a field at a magnification sufficient to distinguish the margins of adjacent cell nuclei and enable accurate image analysis [26, 29]. Although the CFP-AR-YFP reporter also expresses CFP, CFP fluorescence was not quantified because the loss of CFP fluorescence by energy transfer to YFP changes with the types of AR ligands available [26, 35, 45, 46]. Thus, the ligand-dependent loss of CFP fluorescence confounds quantification of CFP amount in the nucleus in a mixture of unknown ligands. By contrast, the amounts of fluorescence emitted upon selective YFP excitation/measurement is not impacted by energy transfer allowing YFP quantification to accurately measure AR level in the nucleus. Another confounding variable of CFP measurement is a very high level of autofluorescence from the cell culture media in the channel used to collect CFP. The resulting weak CFP signal against a high background noise can be corrected for laboriously [29], but the introduction of such imprecision into quantification of the CFP-tagged AR fluorescence in the cell nucleus is unnecessary given the YFP measurement. The mCherry image was used to define the margins of each cell nucleus using the ‘Count Nuclei’ program of the IXMicro analysis software (Molecular Devices Corp). The YFP fluorescence image was used to detect the margins of the cytoplasm outside of the cell nucleus using the ‘Cell Scoring’ program of the analysis software. The ‘background’ fluorescence intensities over the non-cellular areas, together with calibration images that correct for the uneven background fluorescence originating with optical effects, were used to background-correct YFP intensity levels (see section 3.5 in [29]). The average correction relative to the calibration image in wells without and with urine were identical (2.20 vs 2.17 fluorescence intensity units on a 0–4095 scale), which indicates that the urine samples were not independently fluorescent.

The amounts of background-subtracted mCherry and YFP fluorescence intensities within the mCherry-defined nuclear areas were averaged from all nuclei in each of two fields collected for each well. Those measurements were transferred, together with other quality control information (see below) into Microsoft Excel (Redmond, WA, USA). The average YFP fluorescence intensity within the nuclei was compared for the two fields within each well. If they varied more than 30% from each other, these wells were flagged as inaccurate. When encountered, which was seldom, visual inspection indicated that one of the fields either had some non-cellular fluorescent object present or was grossly out of focus. Discarding those fields helps to quality control the data for improved accuracy. Also examined were other quality controls expected to remain constant from well to well, including the average size of the cell nuclei and the average mCherry intensity within the cell nuclei.

Alternative AR quantification methods to the measurement of the average fluorescence intensity of YFP-tagged AR in the cell nucleus also were evaluated. Those alternatives included the total amount of background-subtracted YFP-AR fluorescence in the nucleus (i.e., average intensity per pixel x number of pixels), the percent of background-subtracted AR fluorescence in the nucleus over the total background-subtracted AR fluorescence in the cell (nucleus and cytoplasm), and the ratio of the average fluorescence intensities in the nucleus relative to the cytoplasm. The presence of the mCherry-tagged nuclear marker enabled precise identification of the nuclear boundaries whereas, in the absence of a cytoplasmic marker, the boundaries of the cell were less rigorously identified particularly when cell density was high obfuscating the boundaries between cells. As a result, measurements limited to quantification of AR levels in the nucleus were found to be most precise and had the added benefit of being amenable to high cellular density (i.e., improved averaging from larger numbers of cells per field) and compatible with rapid collection of only two fluorescent channels (nuclear marker and YFP-tagged AR). We therefore conducted our quantification using the most robust and rapid measurement of YFP-tagged AR fluorescence intensity in the cell nucleus.

### Correcting Urine Amplification of YFP fluorescence

Control studies which compared T dose response curves without and with urine showed that most urine samples directly affected the intensity of the YFP reporter (although not the mCherry nuclear marker). The origin of this proportional amplification in YFP fluorescence intensity is unknown, although YFP fluorescence is known to be environmentally sensitive, particularly to Cl^-^ concentration and/or pH [50, 51]. Unless corrected for, the urine effects on YFP intensity would introduce errors into the extrapolation of androgen concentration from the non-amplified T dose response curve.

To determine the extent of YFP amplification, each urine sample was treated in parallel with 10^-7^M T, which is sufficient to saturate the response of the AR BioAssay to T (Fig 1B). Urine addition amplified this maximal background-subtracted YFP fluorescence intensity 1.18 ± 0.05 fold (range: 1.04 to 1.29). The amplification-factor, unique to each urine sample, then was used to correct the YFP fluorescence intensity measured in urine samples with no T added to the YFP intensity that would be present without the amplification effect. This correction was empirically validated by the finding, in T-supplemented subjects whose urinary T measurements ranged from 0.4 to 336 ng/ml, of a 1:1 correlation in AR BioAssay androgen measurement with MS measurement of T (Fig 2).

### Human Subjects

The study and human subjects protocol (10–04232), including the written consent form signed by study participants, were reviewed and approved by the UCSF Committee on Human Research Institutional Review Board. Urine samples were provided by consented subjects undergoing evaluation or T-supplementation therapy for hypogonadism at the UCSF Endocrine Clinic. Subjects receiving T-supplementation by injection were preferred for tracking the rise and decline in urinary T levels following T-injection (Fig 3). Samples were anonymized and de-identified prior to measurement according to the procedures of the approved protocol. No patient records were accessed for this report. As only two Endocrine Clinic subjects were not T-supplemented, additional non-T-supplemented urine samples were provided by a verbally consented, healthy volunteer with detailed knowledge of the protocol. Per study participation guidelines, the volunteer was an individual in a supervisory capacity limiting any concerns about any coercion of a subordinate to participate. Verbal consent was reported to the Institutional Review Board which approved the inclusion of this subject within this report.

### BioAssay Dose Response Curves

51 steroids, AASs, biosynthetic intermediates and metabolites were obtained from the sources listed in S1 Fig. AR BioAssay cells were prepared (see above) and exposed to 1og10 (3.1623-fold) serial dose response curves ranging from 10^−6^ to 10^-13^M final concentration in the 40 μl BioAssay volume. Each dose point was added to triplicate wells, each of which had two fields collected per well. Dual field collections acted as a control for measurement accuracy, as described in the “Quantification’ section. For the standard T curve, a greater density of dilution points between 10^−7^ to 10^-10^M was collected (Fig 1) to improve the extrapolation of androgen levels from the standard curve.

Dose response curves were prepared independently on different days (see Table 1, n). For each dose response curve, the minimum and maximum intensities and the EC50s were established by the best fit to the data points according to the equation Y = Bottom + (Top-Bottom)/(1+10^((LogEC50-X)*HillSlope)), where Y is YFP fluorescence intensity in the cell nucleus and X is the Log molar concentration of the compound. The software used for this non-linear regression analysis was Prism (GraphPad; San Diego, CA). The efficacy (Figs 4 and 6A) and EC50 (Figs 5 and 6B) responses shown are the mean ± standard deviation of the independent replicates. The extrapolation of urinary androgen concentrations against a T standard curve minimized day-to-day errors in the creation of curves by using an average of 52 dose response curves fit to the Top and Bottom values calculated on the day of the study.

### Statistical Analysis

Averages are presented at the mean ± standard deviation from multiple independent studies. The source data for the urine measurements (Figs 2 and 3) are provided in S1 Source Data. The EC50 and maximal BioAssay activity relative to T for all independent dose response curves averaged in the Figs 4–6 are provided in S2 Source Data and S3 Source Data. The comparison of AR BioAssay measurement of androgen concentration with MS measurement of T (Fig 2) was conducted by linear regression analysis using Microsoft Excel. Methods of statistical comparisons, noted in the text, also were conducted in Microsoft Excel or in GraphPad Prism.

## Supporting Information

S1 Fig Compounds investigated. Structures, sources, CAS number and number of independent studies averaged for compounds described in Figs 4–6.
(PDF)

S2 Fig Anti-Androgen Response of AR BioAssay. AR BioAssay is not affected by incubation with 5 μM of the four indicated anti-androgens whereas co-incubation with 5 μM of those anti-androgens with 5 nM DHT diminishes the strong AR BioAssay response to DHT.
(PDF)

S3 Fig EpiT AR BioAssay. **A,** Crystal structure of human AR (left panel) bound with DHT. AR amino acids T877 and L704 (yellow, right panels) flank carbon-17 in in the ‘D-ring’ of T and DHT. Structure is displayed using Cn3D from the publicly deposited coordinates of Zhou et al. [52]. The C-17 hydroxyl group projects ‘above’ the D ring in DHT/T (C17-β, arrow) and ‘below’ the D-ring in epiT (C17-α, not shown). The 17β-OH of T hydrogen bonds with the hydroxyl group at AR amino acid T877 whereas the 17α-OH in epiT would project away from T877. **B,** Fluorescence microscopy images depicting a selective response of the AR BioAssay (left panels) and the epiT AR BioAssay (right panels) to T and epiT, respectively. To design an epiT-binding AR, the hydrogen bond between T and AR amino acid T877 was disrupted by changing T877 to V, L or I. Those AR mutants were unresponsive to T and to epiT (not shown). To enable epiT binding, L704 on the 17α side of the D-ring was replaced with OH-containing amino acids (T or S) to generate a hydrogen bond to epiT. Only the L704T/T877V double mutant created a sensor that responded to epiT but poorly to T. The epiT AR BioAssay also included a V715M mutation that further sensitized the epiT AR BioAssay to epiT (not shown), possibly by creating a more snug interaction with the steroid ring. **C,** Quantification of nuclear YFP (AR) fluorescence levels in response to different doses of T or epiT. The normal specificity of AR for T over epiT (left panel) was reversed for the epiT AR BioAssay (right panel). The epiT AR BioAssay did not respond to 10^-6^M of the steroids and steroid metabolites listed in Fig 4A (not shown). However, the sensitivity of the epiT AR BioAssay for epiT was 100-fold less sensitive than that of the AR BioAssay for T and was insufficiently sensitive for detecting epiT at physiologic levels. These studies show it is possible to generate selective epiT BioAssays, but further improvements are needed to create one suitable for PED analysis.
(PDF)

S1 Source Data Source data for Figs 2 and 3. AR BioAssay measurement of androgen concentrations and mass spectrometry measurements of T and epiT concentrations for each of the 39 urine samples examined in this study.
(PDF)

S2 Source Data Source data for potency presented in Figs 1, 4, 5 and 6. Dose response curves were conducted for each of the 51 compounds examined in examine in triplicate wells (two fields per well) in 45 384-well plates prepared independently on 22 separate days. Data shown represent the outcome of curve fitting (top of curve, bottom of curve and EC50) for each of the 22 studies. Empty fields in the figure represent days in which those particular compounds were not examined. The mean EC50 for each compound is shown above the first set of data for each compound (pages 1, 7, 13, 19, 25, 31, 37, 43, 49).
(PDF)

S3 Source Data Source data for efficacy presented in Figs 1, 4, 5 and 6. The difference between the top and bottom of the dose response curves (see data in S2 Source Data) for each compound was compared against that of testosterone measured in parallel. The efficacy relative to testosterone was calculated and shown in this supporting figure. For ligands that do not substantially activate the AR BioAssay, curve fitting can result in nonsensical tops of the curve. In those instances, the AR BioAssay activity at 1 μM compound is shown relative to that of the maximal activation by testosterone.
(PDF)
