# Supplementary material for: Advantages and Limitations of Androgen Receptor-Based Methods for Detecting Anabolic Androgenic Steroid Abuse as Performance Enhancing Drugs
Source: PLoS One. 2016 Mar 21;11(3):e0151860. doi: 10.1371/journal.pone.0151860 (PMC4801337; doi:10.1371/journal.pone.0151860)

A1, Testosterone

CAS: 58-22-0
Sigma
#T1500
n = 52

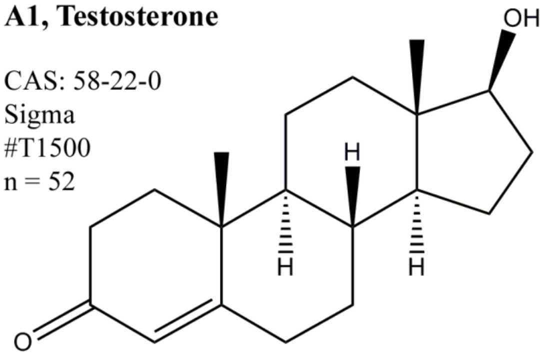

A2, DiHydroTestosterone

CAS: 521-18-6
Sigma
#A8380
n = 6

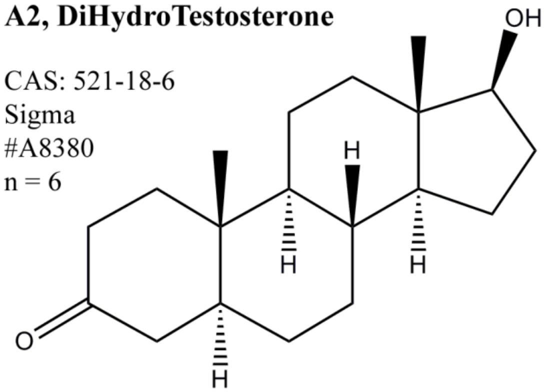

A3, Androstanediol

CAS: 571-20-0
Steraloids
#A1220-000
n = 7

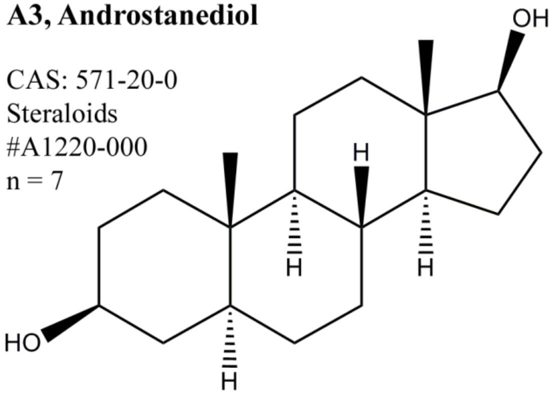

A4, Androstenedione

CAS: 63-05-8
Toronto Res. Chem.
#A637550
n = 12

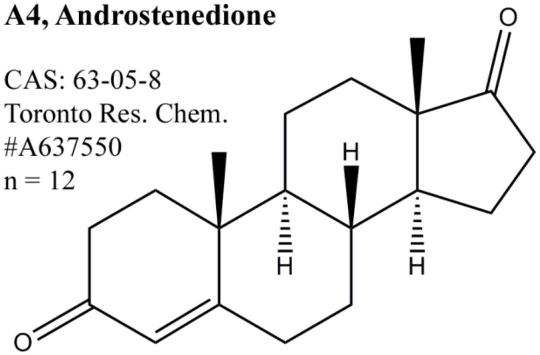

A5, 4-Androstenediol

CAS: 1156-92-9
Steraloids
#A5600-000
n = 9

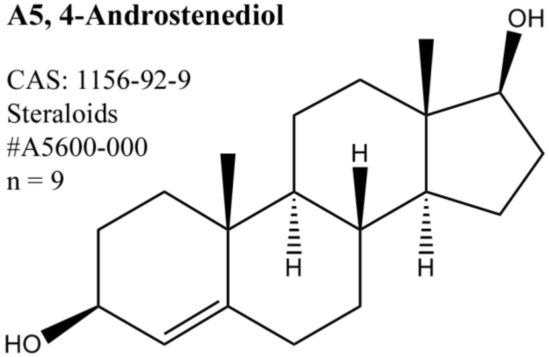

A6, 5-Androstenediol

CAS: 521-17-5
Steraloids
#A7830-000
n = 6

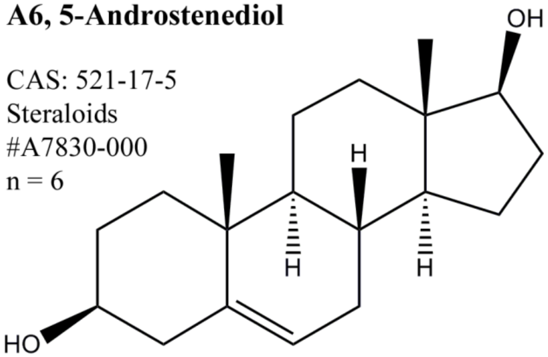

A7, epiTestosterone

CAS: 481-30-1
Sigma
#E5878
n = 4

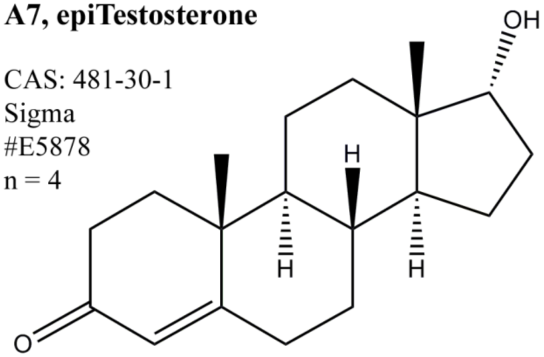

A8, DeHydroEpiAndrosterone

CAS: 53-43-0
Sigma
#D4000
n = 7

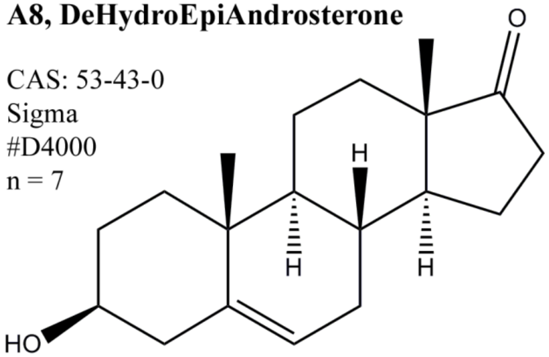

A9, Androsterone

CAS: 53-41-8
Toronto Res. Chem
#A637535
n = 6

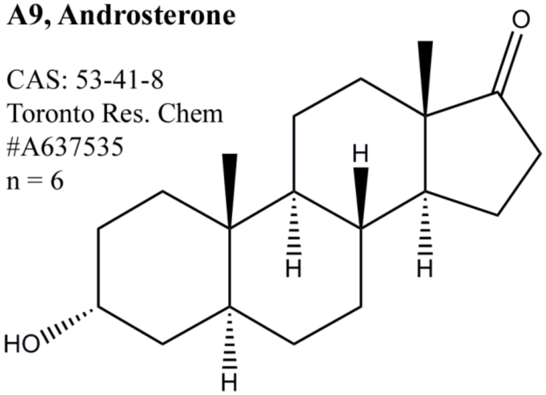

P10, 5α-Pregnan-17α-ol-3,20-dione

CAS: 570-59-2
Steraloids
#P3700-000
n = 6

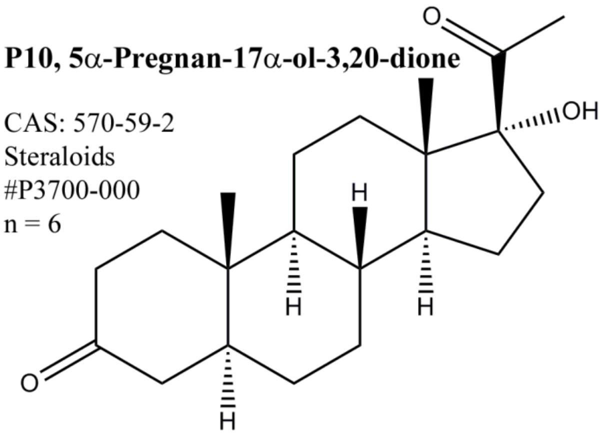

P11, 17α-OH-Progesterone

CAS: 68-96-2
Sigma
#H5752
n = 7

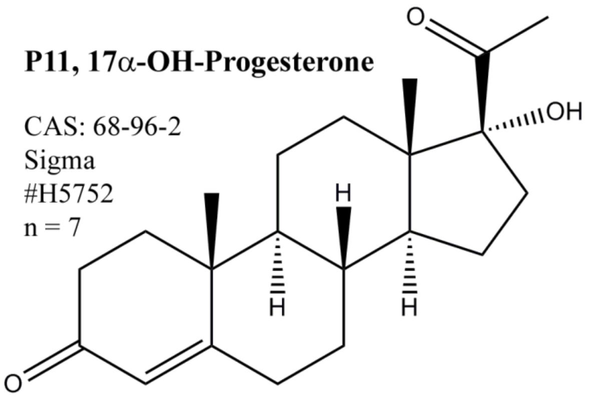

P12, 17α-OH-Pregnenolone

CAS: 387-79-1
Sigma
#H5002
n = 4

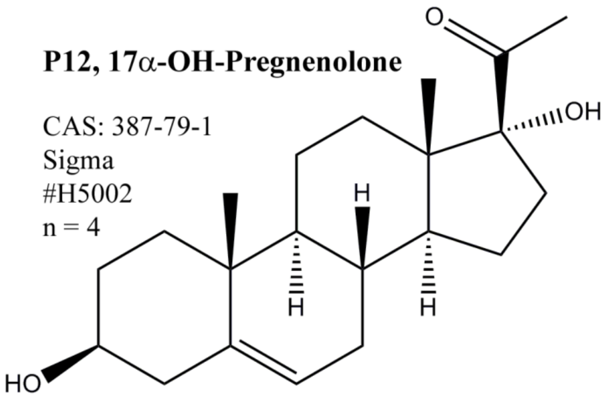

P13, Pregnenolone

CAS: 145-13-1
Sigma
#P9129
n = 4

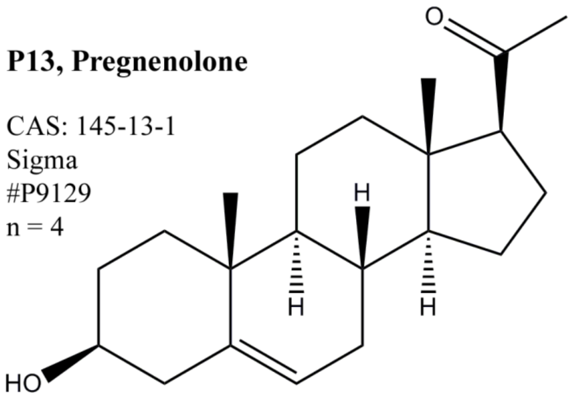

P14, Progesterone

CAS: 57-83-0
Sigma
#P0130
n = 4

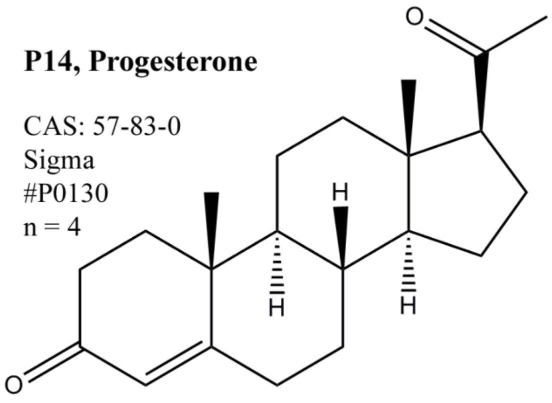

C15, 11-deoxyCorticosterone

CAS: 64-85-7
Sigma
#D105
n = 4

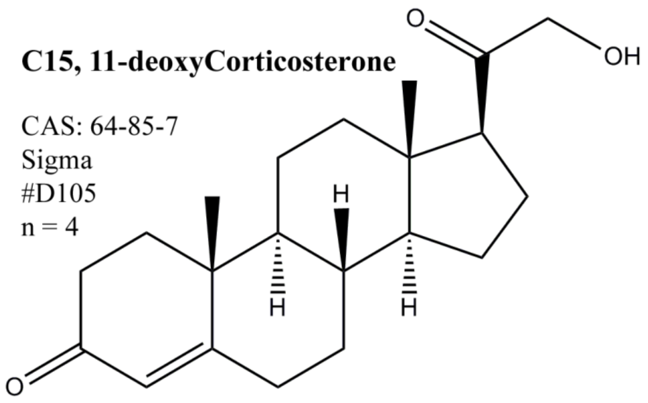

C16, 11-deoxyCortisol

CAS: 152-58-9
Sigma
#R0500
n = 4

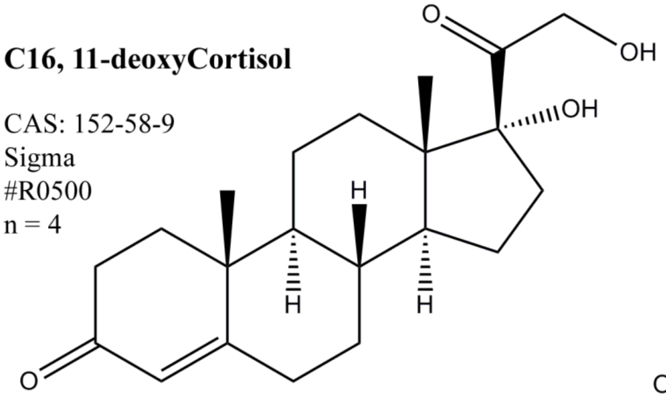

C17, Corticosterone

CAS: 50-22-6
Sigma
#C2505
n = 4

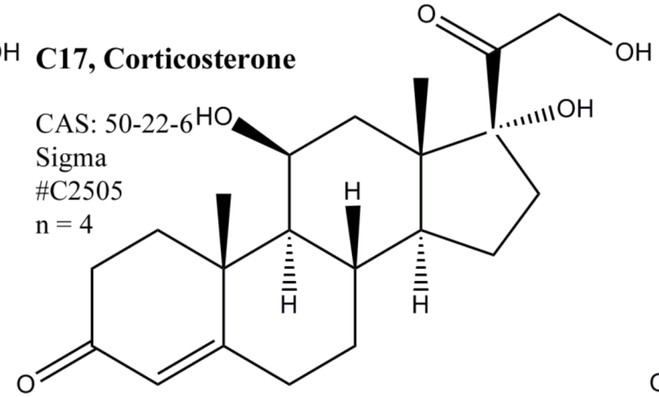

C18, Aldosterone

CAS: 52-39-1
Sigma
#A9477
n = 4

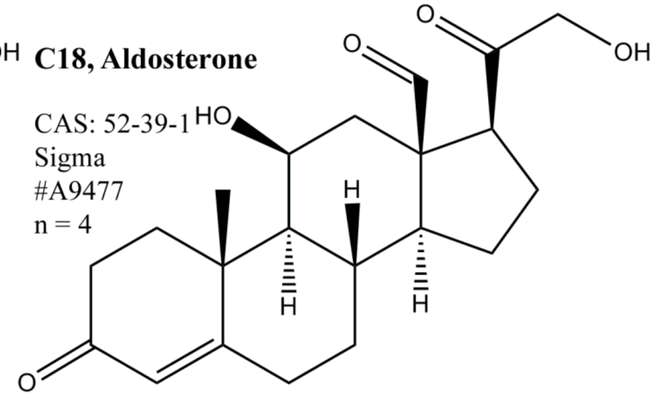

C19, Cortisol

CAS: 50-23-7
Sigma
#H4001
n = 4

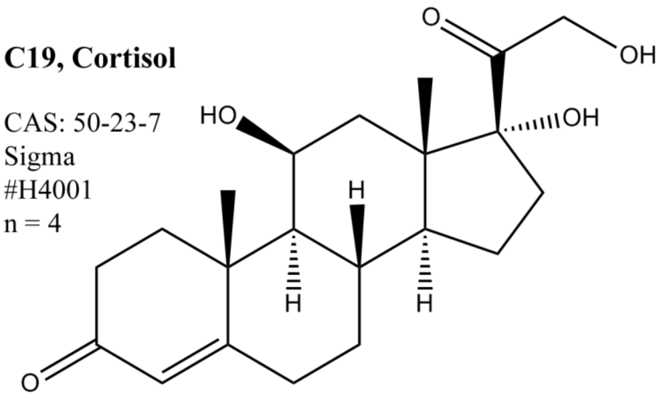

E20, Estradiol

CAS: 50-28-2
Sigma
#E1024
n = 7

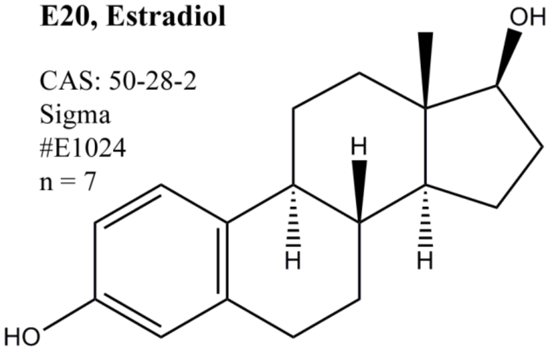

E21, Estrone

CAS: 53-16-7
Sigma
#(E9750)
n = 4

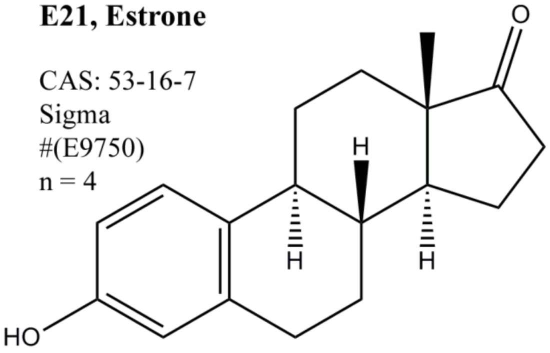

E22, Estriol

CAS: 50-27-1
Sigma
#(E1253)
n = 8

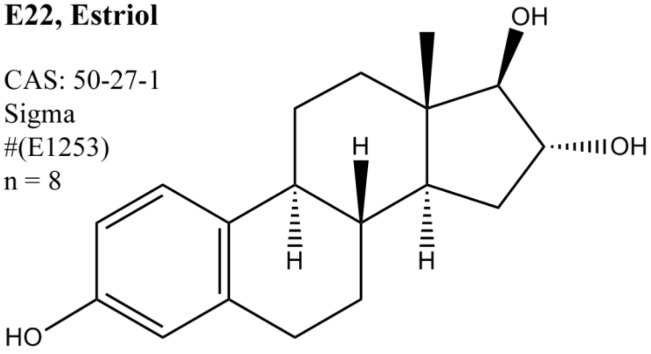

23, Cholesterol

CAS: 57-88-5
Sigma
#(C8667)
n = 4

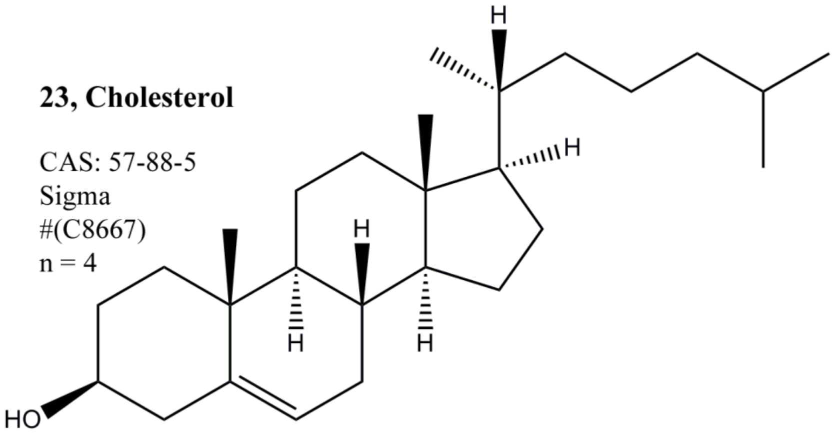

AAS24, TH-Gestrinone

CAS: 618903-56-3
Toronto Res. Chem.
#T29360
n = 2

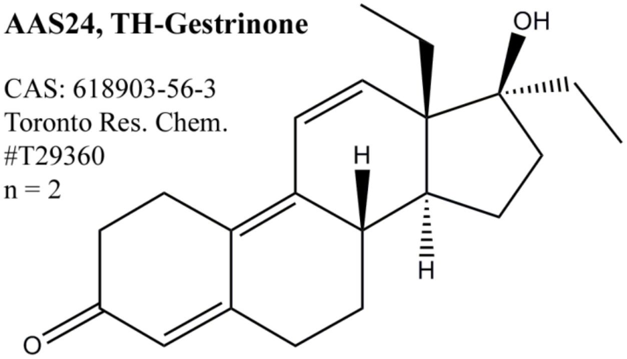

AAS25, Mibolerone

CAS: 3704-09-4
Toronto Res. Chem.
#M342000
n = 2

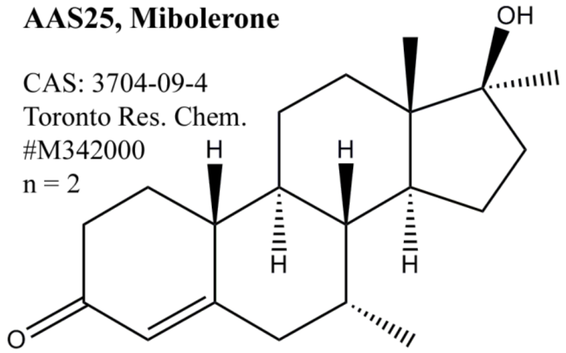

(The three catalog numbers in parentheses are those currently available from Sigma, although the stocks used were from chemicals with discontinued catalog numbers..)

AAS26, Mestanolone

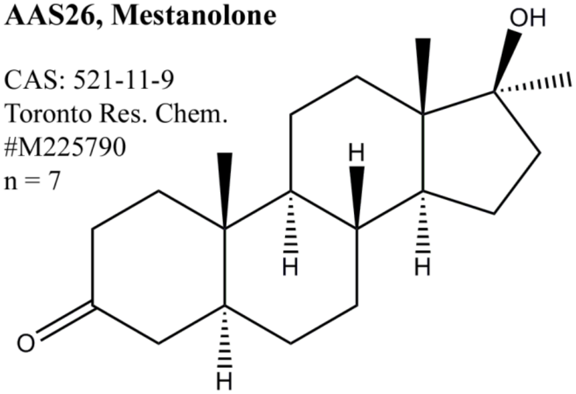

CAS: 521-11-9
Toronto Res. Chem.
#M225790
n = 7

m26/33a, metabolite 'a' of AAS26&33

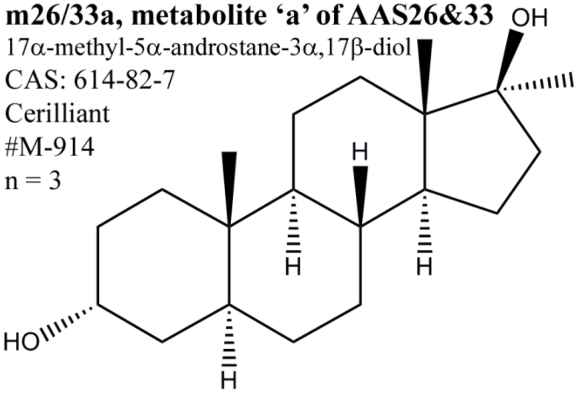

17α-methyl-5α-androstane-3α,17β-diol
CAS: 614-82-7
Cerilliant
#M-914
n = 3

m26/33b, metabolite 'b' of AAS26&33

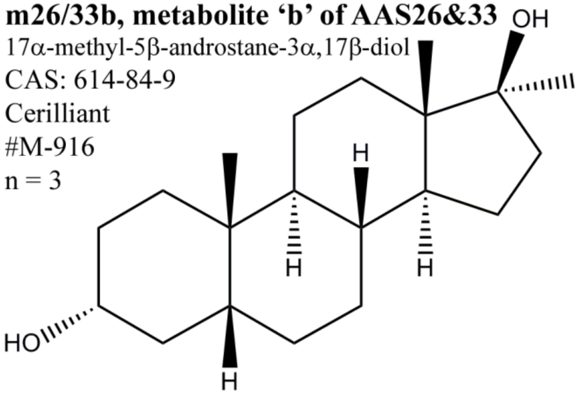

17α-methyl-5β-androstane-3α,17β-diol
CAS: 614-84-9
Cerilliant
#M-916
n = 3

AAS27, Mesterolone

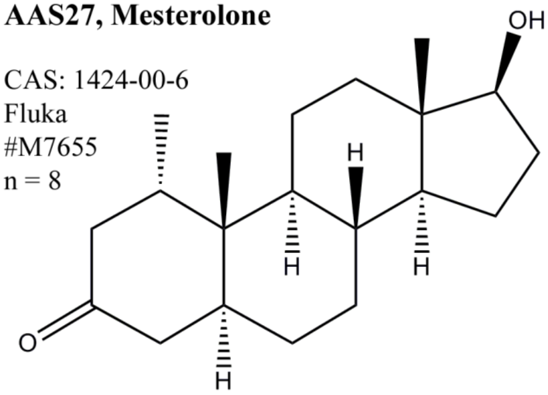

CAS: 1424-00-6
Fluka
#M7655
n = 8

m27, metabolite of AAS27

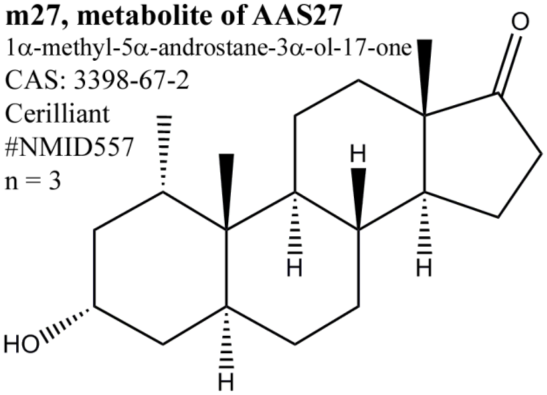

1α-methyl-5α-androstane-3α-ol-17-one
CAS: 3398-67-2
Cerilliant
#NMID557
n = 3

AAS28, Normethandrone

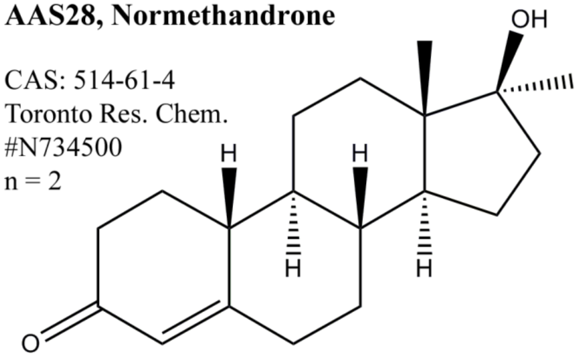

CAS: 514-61-4
Toronto Res. Chem.
#N734500
n = 2

AAS29, Gestrinone

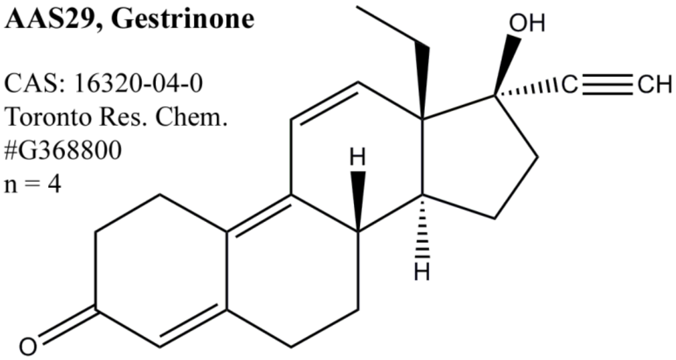

CAS: 16320-04-0
Toronto Res. Chem.
#G368800
n = 4

AAS30, 19-Nortestosterone

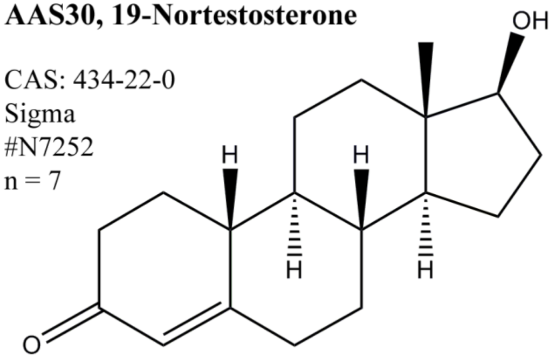

CAS: 434-22-0
Sigma
#N7252
n = 7

m30, metabolite of AAS30

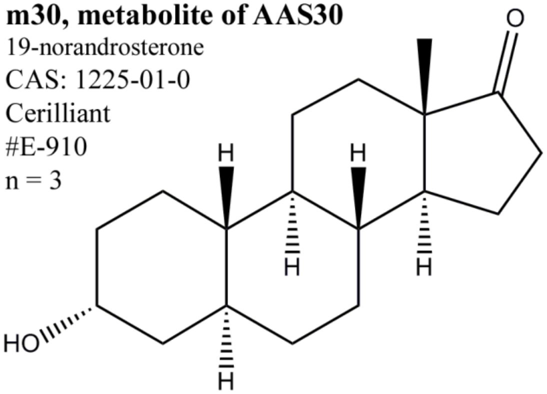

19-norandrosterone
CAS: 1225-01-0
Cerilliant
#E-910
n = 3

AAS31, Trenbolone

CAS: 10161-33-8
Fluka
#T3925
n = 7

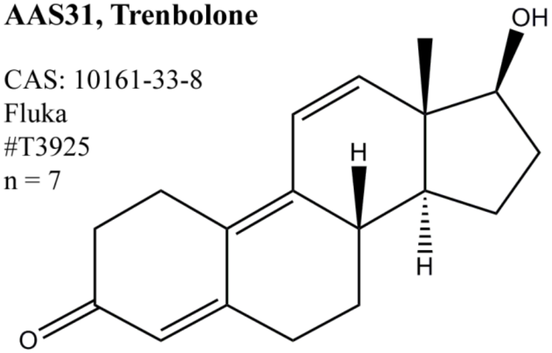

m31, metabolite of AAS31
epitrenbolone
CAS: 80657-17-6
Cerilliant
#NMID708
n = 3

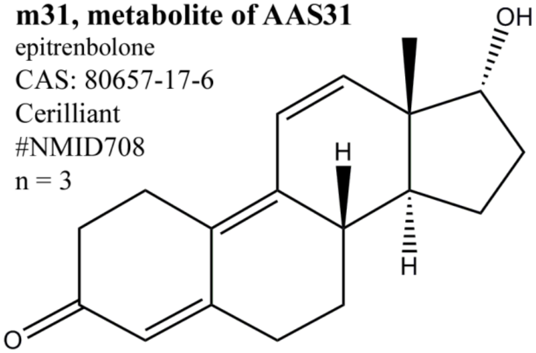

AAS32, Tibolone

CAS: 5630-53-5
Sigma
#T0827
n = 4

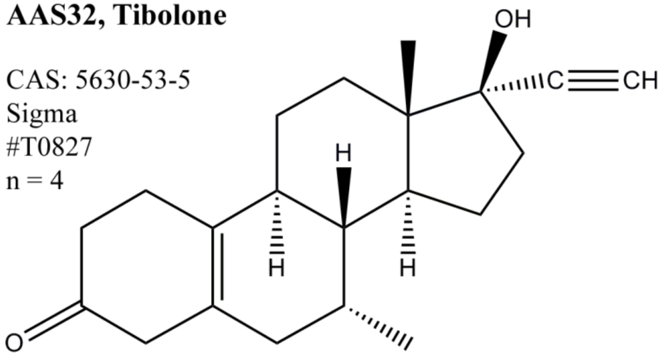

m32, metabolite of AAS32
3-OH-tibolone (3-α/β mixture)
CAS: 100239-45-0 (β)
UCLA Olympic
Anal. Lab synthesis
n = 3

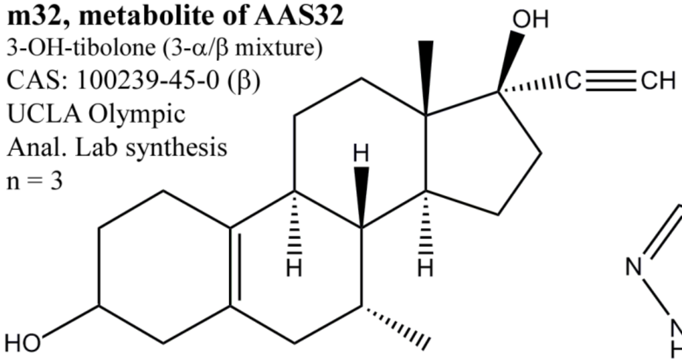

AAS34, Stanozolol

CAS: 10418-03-8
Fluka
#S7132
n = 5

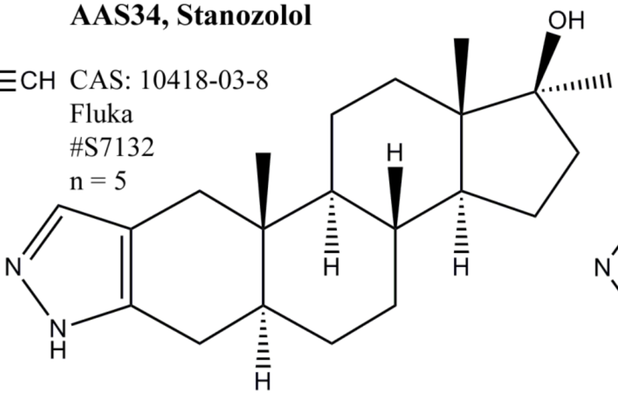

m34, metabolite ‘a’ of AAS34
16β-OH-stanozolol
CAS: 125590-76-3, for α/β
Cerilliant
#H-915
n = 3

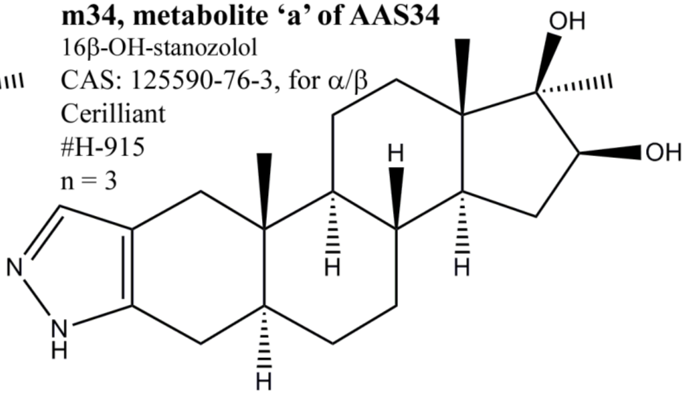

AAS33, Methyltestosterone

CAS: 58-18-4
Sigma
#M7252
n = 7

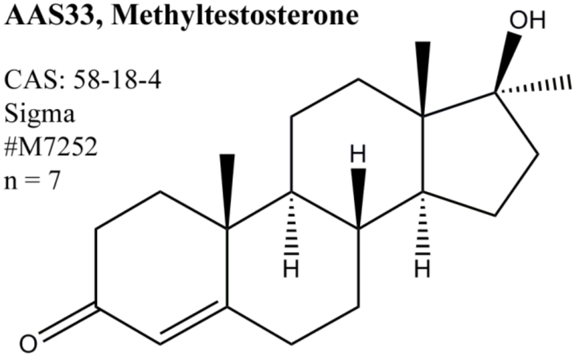

m34, metabolite ‘b’ of AAS34
3’-OH-stanozolol
CAS: 125709-39-9
Cerilliant
#NIMD577
n = 4

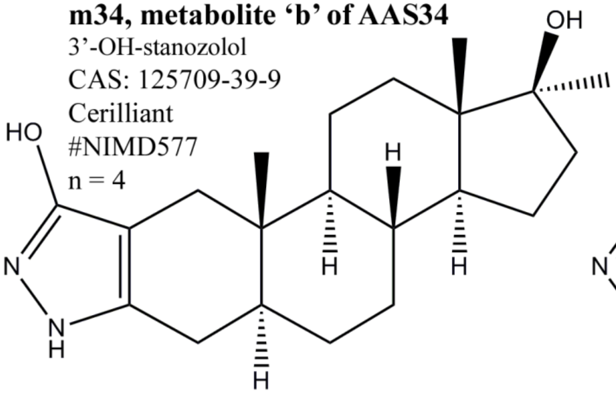

m34, metabolite ‘c’ of AAS34
4β-OH-stanozolol
CAS: 125636-92-2
Cerilliant
#NMID641
n = 3

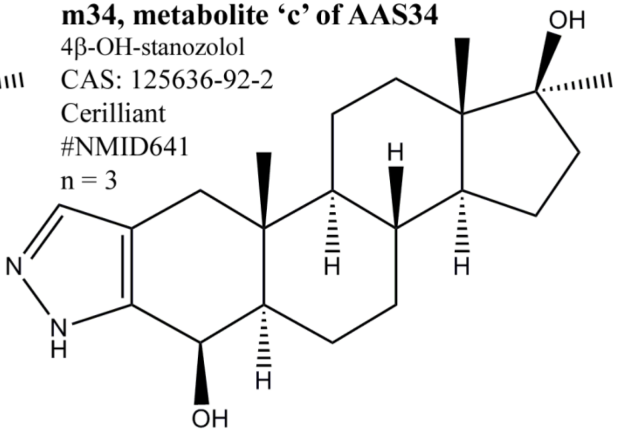

AAS35, Fluoxymesterone

CAS: 76-43-7
Sigma
#F7751
n = 6

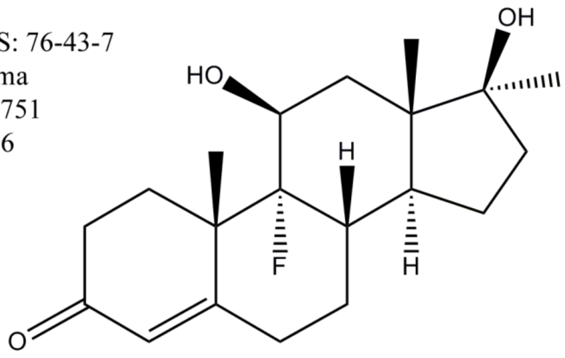

m35, metabolite of AAS35
9α-Fl-17αmethyl-androst-4-ene-α,6β,11β,17β-tetrol
CAS: 148505-57-1
Cerilliant
#NMID616
n = 3

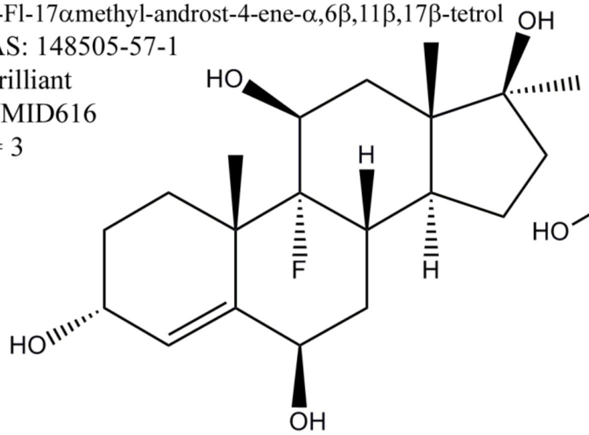

AAS36, Oxymetholone

CAS: 434-07-1
Toronto Res. Chem.
#O876500
n = 2

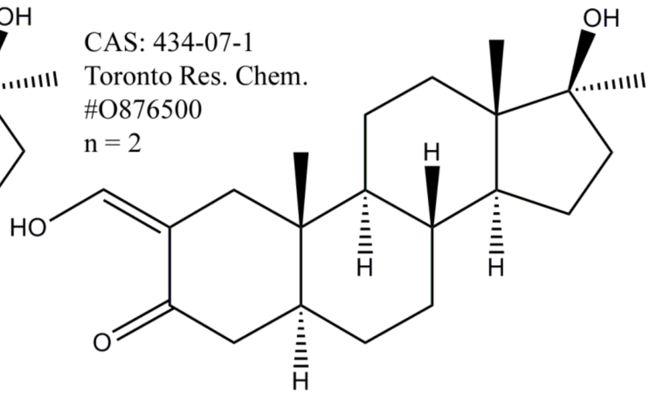

AAS37, Danazol

CAS: 17230-88-5
MP Biomed
#151463
n = 7

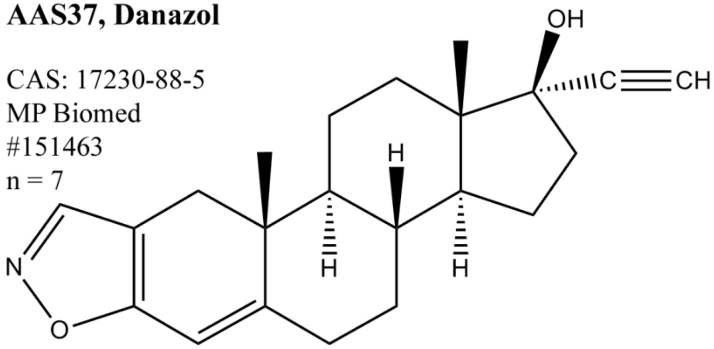

AAS38, 19-Norandrostenedione

CAS: 734-32-7
Toronto Res. Chem.
#N661000
n = 4

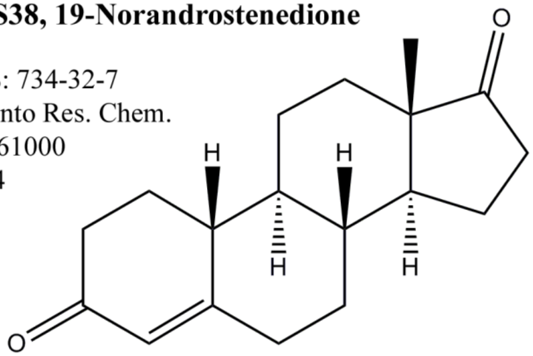

AAS39, Boldione

CAS: 897-06-3
Toronto Res. Chem.
#B675200
n = 7

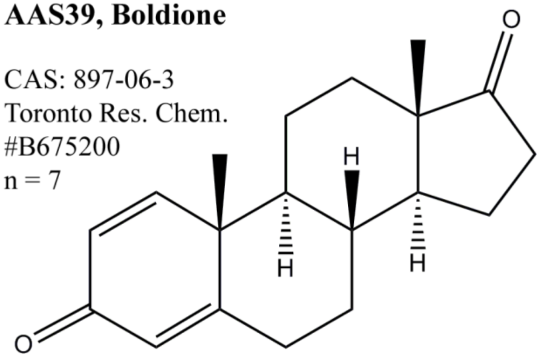

m37, metabolite of AAS37
17α-ethynyl-testosterone
CAS: 434-03-7
Sigma
#E1001
n = 3

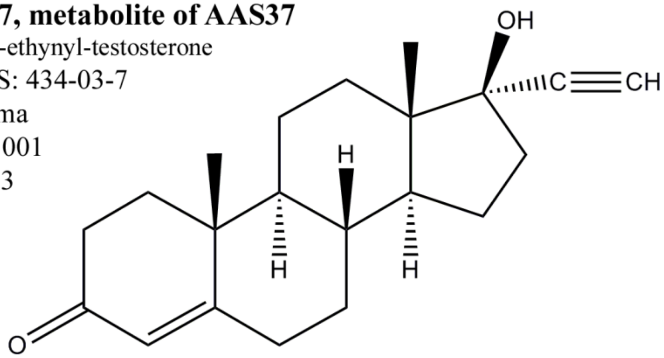

m39, metabolite of AAS39
5β-androst-1en-17β-ol-3-one
CAS: 10529-96-1
Cerilliant
#NMID564
n = 3

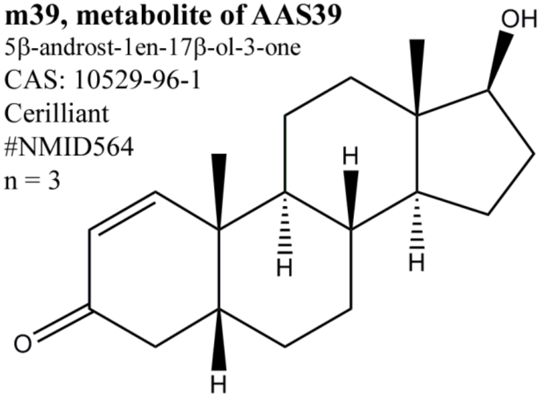

Supplement: S1 Fig — Structures, sources, CAS number and number of independent studies averaged for compounds described in Figs 4–6. (PDF) [file pone.0151860.s001.pdf]
