# Supplementary figures and images for: Advantages and Limitations of Androgen Receptor-Based Methods for Detecting Anabolic Androgenic Steroid Abuse as Performance Enhancing Drugs
Source: PLoS One. 2016 Mar 21;11(3):e0151860. doi: 10.1371/journal.pone.0151860 (PMC4801337; doi:10.1371/journal.pone.0151860)

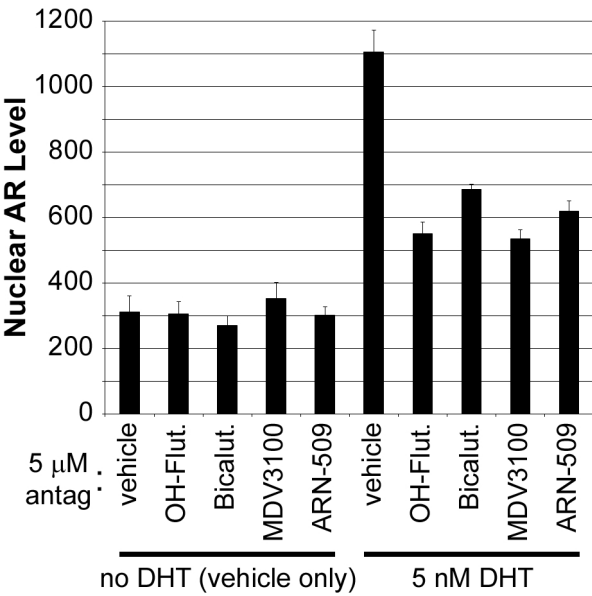

Supplement: S2 Fig — AR BioAssay is not affected by incubation with 5 μM of the four indicated anti-androgens whereas co-incubation with 5 μM of those anti-androgens with 5 nM DHT diminishes the strong AR BioAssay response to DHT. (PDF) [file pone.0151860.s002.pdf]

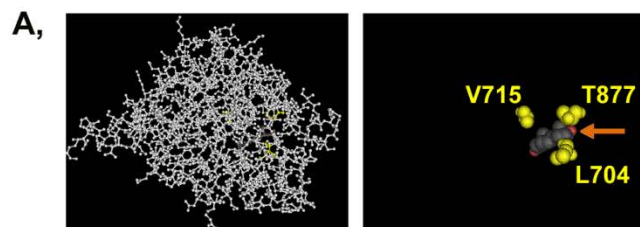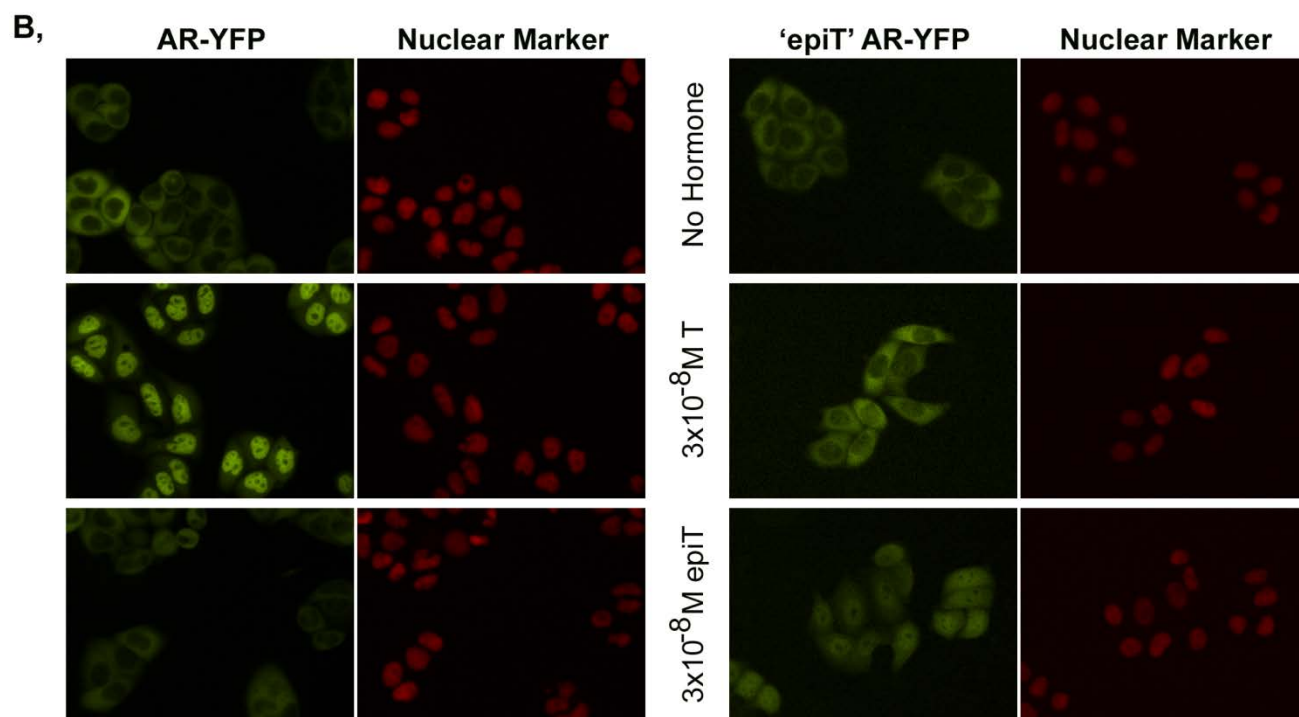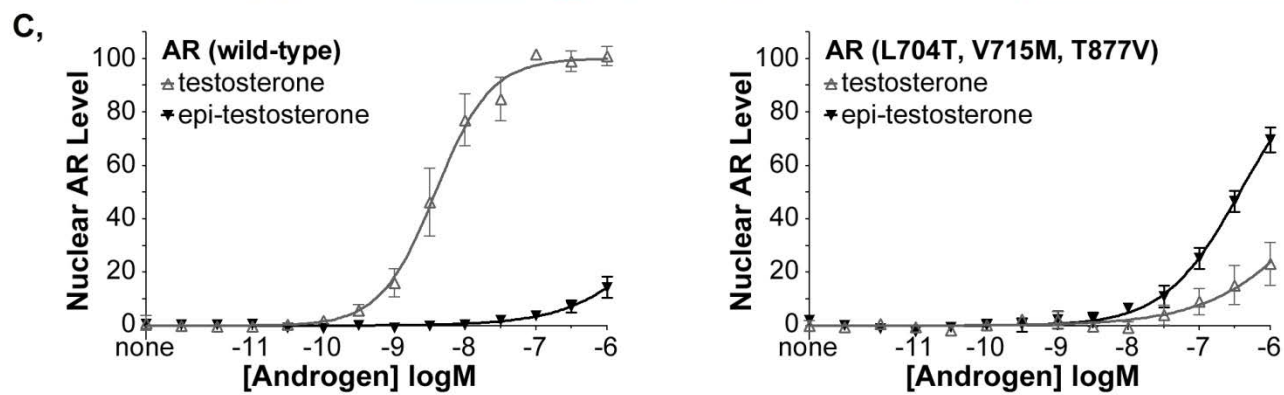

Supplement: S3 Fig — A, Crystal structure of human AR (left panel) bound with DHT. AR amino acids T877 and L704 (yellow, right panels) flank carbon-17 in in the ‘D-ring’ of T and DHT. Structure is displayed using Cn3D from the publicly deposited coordinates of Zhou et al. [52]. The C-17 hydroxyl group projects ‘above’ the D ring in DHT/T (C17-β, arrow) and ‘below’ the D-ring in epiT (C17-α, not shown). The 17β-OH of T hydrogen bonds with the hydroxyl group at AR amino acid T877 whereas the 17α-OH in epiT would project away from T877. B, Fluorescence microscopy images depicting a selective response of the AR BioAssay (left panels) and the epiT AR BioAssay (right panels) to T and epiT, respectively. To design an epiT-binding AR, the hydrogen bond between T and AR amino acid T877 was disrupted by changing T877 to V, L or I. Those AR mutants were unresponsive to T and to epiT (not shown). To enable epiT binding, L704 on the 17α side of the D-ring was replaced with OH-containing amino acids (T or S) to generate a hydrogen bond to epiT. Only the L704T/T877V double mutant created a sensor that responded to epiT but poorly to T. The epiT AR BioAssay also included a V715M mutation that further sensitized the epiT AR BioAssay to epiT (not shown), possibly by creating a more snug interaction with the steroid ring. C, Quantification of nuclear YFP (AR) fluorescence levels in response to different doses of T or epiT. The normal specificity of AR for T over epiT (left panel) was reversed for the epiT AR BioAssay (right panel). The epiT AR BioAssay did not respond to 10-6M of the steroids and steroid metabolites listed in Fig 4A (not shown). However, the sensitivity of the epiT AR BioAssay for epiT was 100-fold less sensitive than that of the AR BioAssay for T and was insufficiently sensitive for detecting epiT at physiologic levels. These studies show it is possible to generate selective epiT BioAssays, but further improvements are needed to create one suitable for PED analysis. (PDF) [file pone.0151860.s003.pdf]
