# Supplementary material for: Advantages and Limitations of Androgen Receptor-Based Methods for Detecting Anabolic Androgenic Steroid Abuse as Performance Enhancing Drugs
Source: PLoS One. 2016 Mar 21;11(3):e0151860. doi: 10.1371/journal.pone.0151860 (PMC4801337; doi:10.1371/journal.pone.0151860)
Supplement: S1 Source Data — AR BioAssay measurement of androgen concentrations and mass spectrometry measurements of T and epiT concentrations for each of the 39 urine samples examined in this study. (PDF) [file pone.0151860.s004.pdf]

						Detection Limit>		8	(ng/dl)	
**Urine Sample #**	**Subject #**	**T treatment**	**BioAssay [androgen]**	**MS [T]**	**MS [epiT]**	**BioAssay [androgen]**	**MS [T]**	**[androgen] / MS [T]**	**MS [T] / MS [epiT]**	**[androgen] / MS [epiT]**
			T-treated		T-treated					
			R2	0.98	avg	94.28	104.13	0.96	22.39	19.81
			Slope	1.04	sd	99.32	102.89	0.22	16.78	13.93
			Intercept	2.4	n	11	11	11	11	11
					Ttest androgen vs T:		0.20			
			No T-treatment		No T-treatment					
			R2	0.89	avg	30.57	27.47	1.13	1.03	1.15
			Slope	1.07	sd	11.44	9.02	0.23	0.17	0.24
			Intercept	1.1	n	18	18	18	18	18
						Ttest T-treat vs none:		0.03	0.00	0.00
1	2	T-treated	**1.7**	**0.8**	**9.7**					
2	3	T-treated	**14.6**	**12.3**	**0.8**	**14.6**	**12.3**	1.19	15.38	18.29
3	3	T-treated	**48.5**	**110.4**	**2.1**	**48.5**	**110.4**	0.44	52.57	23.08
4	3	T-treated	**19.4**	**18.8**	**1.8**	**19.4**	**18.8**	1.03	10.44	10.77
5	4	T-treated	**67.1**	**78.2**	**19.1**	**67.1**	**78.2**	0.86	4.09	3.51
6	5	T-treated	**2.5**	**0.4**	**0.3**					
7	5	T-treated	**3.4**	**0.6**	**0.3**					
8	5	T-treated	**3.1**	**0.9**	**0.5**					
9	5	T-treated	**2.7**	**0.7**	**0.4**					
10	5	T-treated	**3.5**	**0.8**	**0.4**					
11	5	T-treated	**2.6**	**0.7**	**0.3**					
12	5	T-treated	**3.2**	**1.6**	**0.5**					
13	5	T-treated	**0.4**	**1.1**	**0.4**					
14	8	T-treated	**82.1**	**75.0**	**4.7**	**82.1**	**75.0**	1.09	15.96	17.47
15	8	T-treated	**48.9**	**55.2**	**3.8**	**48.9**	**55.2**	0.89	14.53	12.88
16	8	T-treated	**309.4**	**336.2**	**9.1**	**309.4**	**336.2**	0.92	36.95	34.00
17	8	T-treated	**105.1**	**144.6**	**7.0**	**105.1**	**144.6**	0.73	20.66	15.02
18	8	T-treated	**56.3**	**48.2**	**3.6**	**56.3**	**48.2**	1.17	13.39	15.63
19	8	T-treated	**22.2**	**19.5**	**1.8**	**22.2**	**19.5**	1.14	10.83	12.36
20	8	T-treated	**263.5**	**247.0**	**4.8**	**263.5**	**247.0**	1.07	51.46	54.89

Non-treated samples on next page

21	1	none	**1.3**	**0.1**	**0.1**					
22	6	none	**22.4**	**16.7**	**16.5**	**22.4**	**16.7**	1.34	1.01	1.36
23	6	none	**28.3**	**23.8**	**23.7**	**28.3**	**23.8**	1.19	1.00	1.19
24	6	none	**34.3**	**26.3**	**25.2**	**34.3**	**26.3**	1.30	1.04	1.36
25	6	none	**24.7**	**23.5**	**19.7**	**24.7**	**23.5**	1.05	1.19	1.25
26	6	none	**21.7**	**15.7**	**18.4**	**21.7**	**15.7**	1.38	0.85	1.18
27	6	none	**42.3**	**34.7**	**32.1**	**42.3**	**34.7**	1.22	1.08	1.32
28	6	none	**28.7**	**22.8**	**20.3**	**28.7**	**22.8**	1.26	1.12	1.41
29	6	none	**26.0**	**20.0**	**19.5**	**26.0**	**20.0**	1.30	1.03	1.33
30	6	none	**33.5**	**27.6**	**28.2**	**33.5**	**27.6**	1.21	0.98	1.19
31	6	none	**12.2**	**23.6**	**22.9**	**12.2**	**23.6**	0.52	1.03	0.53
32	6	none	**31.5**	**28.3**	**29.9**	**31.5**	**28.3**	1.11	0.95	1.05
33	6	none	**32.7**	**36.1**	**31.1**	**32.7**	**36.1**	0.91	1.16	1.05
34	6	none	**26.5**	**32.0**	**26.2**	**26.5**	**32.0**	0.83	1.22	1.01
35	6	none	**26.5**	**29.2**	**24.6**	**26.5**	**29.2**	0.91	1.19	1.08
36	6	none	**27.9**	**24.9**	**22.8**	**27.9**	**24.9**	1.12	1.09	1.22
37	6	none	**34.7**	**36.3**	**31.0**	**34.7**	**36.3**	0.96	1.17	1.12
38	6	none	**27.8**	**19.0**	**20.2**	**27.8**	**19.0**	1.46	0.94	1.38
39	7	none	**68.6**	**53.9**	**111.0**	**68.6**	**53.9**	1.27	0.49	0.62

**Urine Sample #**	
1	Urine Collection Times for Subject 3 (Day; Fig 3B)
2	0.375
3	1.750
4	10.375
5	
6	
7	
8	
9	
10	
11	
12	
13	Urine Collection Times for Subject 8 (Day; Fig 3A)
14	0.403
15	2.417
16	3.563
17	5.548
18	9.329
19	10.930
20	11.542
