# Supplementary material for: Advantages and Limitations of Androgen Receptor-Based Methods for Detecting Anabolic Androgenic Steroid Abuse as Performance Enhancing Drugs
Source: PLoS One. 2016 Mar 21;11(3):e0151860. doi: 10.1371/journal.pone.0151860 (PMC4801337; doi:10.1371/journal.pone.0151860)
Supplement: S2 Source Data — Dose response curves were conducted for each of the 51 compounds examined in examine in triplicate wells (two fields per well) in 45 384-well plates prepared independently on 22 separate days. Data shown represent the outcome of curve fitting (top of curve, bottom of curve and EC50) for each of the 22 studies. Empty fields in the figure represent days in which those particular compounds were not examined. The mean EC50 for each compound is shown above the first set of data for each compound (pages 1, 7, 13, 19, 25, 31, 37, 43, 49). (PDF) [file pone.0151860.s005.pdf]

Legend
T-like' efficacy (max reached at 10-6M)
low efficacy (max reached at 10-6M)
unknown efficacy (max not reached at 10-6M)
**no to minimal activity, levels are >amount indicated**

					**A1**	**A2**	**A3**	
					**Testosterone**	**DHT**	**Androstanediol**	
	**avg EC50 (M)**				**2.04E-09**	**5.55E-10**	**1.29E-08**	
	**avg EC50 (nM)**				**2.04**	**0.55**	**12.94**	
	**avg log EC50 for those reaching maximum**				**-8.69**	**-9.26**	**-7.89**	
	**est. log EC50 when no max activity at 10-6M**							
	**avg log EC50**				**-8.69**	**-9.26**	**-7.89**	
	**sd log EC50**				**0.25**	**0.37**	**0.59**	
	**n log EC50**				**46**	**6**	**4**	
	**min log EC50**				**-9.19**	**-9.70**	**-8.61**	
	**max log EC50**				**-7.95**	**-8.65**	**-7.23**	
	sd EC50 lower range				1.15	0.23	3.30	
	sd EC50 upper range				3.61	1.31	50.81	
	MW				288.42	290.44	292.46	
	**ng/ml at EC50**				**0.59**	**0.16**	**3.79**	
	**detectable level in 0.5ul/40ul Assay**				**4.70**	**1.21**	**46.65**	
**Plate #'s**		**Testosterone**	Testosterone	Testosterone	Testosterone	**DHT**	**Androstanediol**	Androstanediol
AQ55	Bottom	227.7						
AQ56	Top	957.1						
	LogEC50	-7.947						
	HillSlope	0.8986						
	EC50	1.13E-08						
	amount at 10-6M	918.5						
**Plate #'s**		**Testosterone**	Testosterone	Testosterone	Testosterone	**DHT**	**Androstanediol**	Androstanediol
AQ286	Bottom	373.3				389.8		
AQ287	Top	1371				1429		
	LogEC50	-8.899				-9.501		
	HillSlope	1.14				2.053		
	EC50	1.26E-09				3.15E-10		
	amount at 10-6M	1437.3				1357.7		

**Plate #'s**		**Testosterone**	Testosterone	Testosterone	Testosterone	**DHT**	**Androstanediol**	Androstanediol
AQ520	Bottom	244.2	228.7	253.8		249.8		
AQ521	Top	1088	1054	1129		1170		
	LogEC50	-8.355	-8.521	-8.45		-9.011		
	HillSlope	1.76	1.805	1.312		1.593		
	EC50	4.42E-09	3.01E-09	3.55E-09		9.75E-10		
	amount at 10-6M	1040.6	1122.1			1212.7		
**Plate #'s**		**Testosterone**	Testosterone	Testosterone	Testosterone	**DHT**	**Androstanediol**	Androstanediol
AQ558	Bottom	204.2	226.5	185.6				
AQ559	Top	1190	1226	1191				
	LogEC50							
	HillSlope							
	EC50							
	amount at 10-6M	1225.9	1216.3	1058.4				
**Plate #'s**		**Testosterone**	Testosterone	Testosterone	Testosterone	**DHT**	**Androstanediol**	Androstanediol
AQ578	Bottom	242.3	179.1	215.3		173.1	185.2	183.6
AQ579	Top	1078	1006	1096		1084	757.4	759
	LogEC50	-8.701	-8.474	-8.711		-8.648	-7.635	-7.225
	HillSlope	1.829	1.662	1.82E+00		1.85	1.279	
	EC50	1.99E-09	3.36E-09	1.95E-09		2.25E-09	2.32E-08	5.96E-08
	amount at 10-6M	1088.2	945.7	1067.7		1010.5	750.2	711.9
**Plate #'s**		**Testosterone**	Testosterone	Testosterone	Testosterone	**DHT**	**Androstanediol**	Androstanediol
AQ595	Bottom	236.5	189.4	196.4	241.6			
AQ597	Top	1599	1676	1626	1794			
	LogEC50	-8.42E+00	-8.51E+00	-8.44E+00	-8.43E+00			
	HillSlope	1.629	1.487	1.599	1.72			
	EC50	3.80E-09	3.07E-09	3.65E-09	3.74E-09			
	amount at 10-6M	1566.5	1600.3	1617.6	1796.8			
**Plate #'s**		**Testosterone**	Testosterone	Testosterone	Testosterone	**DHT**	**Androstanediol**	Androstanediol
AQ636	Bottom	200.5	184.2					
	Top	1023	1038					
	LogEC50	-8.95	-9.047					
	HillSlope	1.411	1.451					
	EC50	1.12E-09	8.97E-10					
	amount at 10-6M	1034.9						

**Plate #'s**		**Testosterone**	Testosterone	Testosterone	Testosterone	**DHT**	**Androstanediol**	Androstanediol
AQ641	Bottom	222.6						
	Top	1063						
	LogEC50	-8.736						
	HillSlope	1.402						
	EC50	1.84E-09						
	amount at 10-6M	1013.2						
**Plate #'s**		**Testosterone**	Testosterone	Testosterone	Testosterone	**DHT**	**Androstanediol**	Androstanediol
AQ662	Bottom	112.5	93.16	110.9	105.6		80.66	
AQ664	Top	697.2	737.8	696.2	791.8		409	
	LogEC50	-8.551	-8.552	-8.586	-8.489		-8.086	
	HillSlope	1.467	1.367	1.69E+00	1.59E+00		1.361	
	EC50	2.82E-09	2.81E-09	2.60E-09	3.24E-09		8.20E-09	
	amount at 10-6M	677.8	736.3	673.3	792.4		379.8	
**Plate #'s**		**Testosterone**	Testosterone	Testosterone	Testosterone	**DHT**	**Androstanediol**	Androstanediol
AQ694	Bottom	71.85	59.11	78.79	80.77			
AQ696	Top	608.6	670.8	612.5	689.6			
	LogEC50	-8.841	-8.354	-8.848	-8.458			
	HillSlope	1.998	1.324	2.102	1.484			
	EC50	1.44E-09	4.42E-09	1.42E-09	3.48E-09			
	amount at 10-6M	639.1	684.2	636.7	698.1			
**Plate #'s**		**Testosterone**	Testosterone	Testosterone	Testosterone	**DHT**	**Androstanediol**	Androstanediol
AQ706	Bottom	48.90					42.23	
	Top	319.90					240.50	
	LogEC50	-8.698					-8.606	
	HillSlope	1.751					1.055	
	EC50	2.01E-09					2.48E-09	
	amount at 10-6M	316.10					232.03	
**Plate #'s**		**Testosterone**	Testosterone	Testosterone	Testosterone	**DHT**	**Androstanediol**	Androstanediol
AQ745	Bottom	54.00	60.92					
AQ746	Top	537.5	563					
	LogEC50	-8.7						
	HillSlope	1.11						
	EC50	2.00E-09						
	amount at 10-6M	535.7	522.0					

**Plate #'s**		**Testosterone**	Testosterone	Testosterone	Testosterone	**DHT**	**Androstanediol**	Androstanediol
AQ295	Bottom	115.5	108.8					
	Top	478.2	452					
	LogEC50	-8.999	-9.192					
	HillSlope	0.7435	1.003					
	EC50	1.00E-09	6.43E-10					
	amount at 10-6M	485.2	435.2					
**Plate #'s**		**Testosterone**	Testosterone	Testosterone	Testosterone	**DHT**	**Androstanediol**	Androstanediol
AQ296	Bottom	95.82	112.3					
	Top	436	463.9					
	LogEC50	-9.104	-9.19					
	HillSlope	0.8921	1.235					
	EC50	7.87E-10	6.46E-10					
	amount at 10-6M	442.4	501.4	464.8				
**Plate #'s**		**Testosterone**	Testosterone	Testosterone	Testosterone	**DHT**	**Androstanediol**	Androstanediol
AQ897	Bottom	98.86						
	Top	708.8						
	LogEC50	-8.483						
	HillSlope	9.289						
	EC50	3.29E-09						
	amount at 10-6M	783.0						
**Plate #'s**		**Testosterone**	Testosterone	Testosterone	Testosterone	**DHT**	**Androstanediol**	Androstanediol
AQ1347	Bottom	139.1	107.0	100.4				
AQ1348	Top	637.9	601.9	659.6				
AQ1349	LogEC50							
AQ1350	HillSlope							
	EC50							
	amount at 10-6M	677.2	641.2	728.7				
**Plate #'s**		**Testosterone**	Testosterone	Testosterone	Testosterone	**DHT**	**Androstanediol**	Androstanediol
AQ1355	Bottom	78.6	82.2	76.8	70.6			
AQ1356	Top	718.5	749.2	785.1	793.9			
AQ1357	LogEC50	-8.481	-8.556	-8.761	-8.662			
AQ1358	HillSlope	1.163	0.997	1.256	1.259			
	EC50	3.31E-09	2.78E-09	1.74E-09	2.18E-09			
	amount at 10-6M	755.5	724.2	810.2	800.0			

**Plate #'s**		**Testosterone**	Testosterone	Testosterone	Testosterone	**DHT**	**Androstanediol**	Androstanediol
AQ1360	Bottom	91.4	105.1	98.6				
AQ1361	Top	758.1	795.2	878.5				
AQ1362	LogEC50	-8.823	-8.830	-8.752				
AQ1363	HillSlope	1.206	1.339	1.464				
	EC50	1.50E-09	1.48E-09	1.77E-09				
	amount at 10-6M	744.5	782.9	896.1				
**Plate #'s**		**Testosterone**	Testosterone	Testosterone	Testosterone	**DHT**	**Androstanediol**	Androstanediol
AQ301	Bottom	222.7				223.4	223.1	
	Top	707.8				743.4	610.2	
	LogEC50	-8.823				-9.354	-7.609	
	HillSlope	1.272				1.510	0.944	
	EC50	1.50E-09				4.43E-10	2.46E-08	
	amount at 10-6M	703.3				785.3	600.8	
**Plate #'s**		**Testosterone**	Testosterone	Testosterone	Testosterone	**DHT**	**Androstanediol**	Androstanediol
AQ312	Bottom	231.5						
	Top	643.7						
	LogEC50	-8.732						
	HillSlope	1.187						
	EC50	1.86E-09						
	amount at 10-6M	641.0						
**Plate #'s**		**Testosterone**	Testosterone	Testosterone	Testosterone	**DHT**	**Androstanediol**	Androstanediol
AQ352	Bottom	187.8						
	Top	395.9						
	LogEC50	-8.682						
	HillSlope	1.204						
	EC50	2.08E-09						
	amount at 10-6M	392.5						
**Plate #'s**		**Testosterone**	Testosterone	Testosterone	Testosterone	**DHT**	**Androstanediol**	Androstanediol
AQ273	Bottom	250.3				244.5	244.2	
	Top	743.4				807.5	574.8	
	LogEC50	-8.791				-9.323	-8.002	
	HillSlope	1.527				1.350	1.584	
	EC50	1.62E-09				4.76E-10	9.95E-09	
	amount at 10-6M	716.7				799.8	571.5	

Plate #'s		Testosterone	Testosterone	Testosterone	Testosterone	DHT	Androstanediol	Androstanediol
AQ327	Bottom	276.8						
	Top	826.0						
	LogEC50	-8.757						
	HillSlope	1.370						
	EC50	1.75E-09						
	amount at 10-6M	840.7						
**Plate #'s**		**Testosterone**	Testosterone	Testosterone	Testosterone	**DHT**	**Androstanediol**	Androstanediol
AQ290	Bottom	272.5						
	Top	767.0						
	LogEC50	-8.738						
	HillSlope	1.434						
	EC50	1.83E-09						
	amount at 10-6M	744.8						
**Plate #'s**		**Testosterone**	Testosterone	Testosterone	Testosterone	**DHT**	**Androstanediol**	Androstanediol
AQ711	Bottom	228.4	237.2			228.4	227.4	
	Top	709.4	755.4			739.2	532.0	
	LogEC50	-9.123	-8.933			-9.698	-7.819	
	HillSlope	1.265	1.315			1.293	1.010	
	EC50	7.54E-10	1.17E-09			2.01E-10	1.52E-08	
	amount at 10-6M	725.6	780.8			755.9	526.8	
**Plate #'s**		**Testosterone**	Testosterone	Testosterone	Testosterone	**DHT**	**Androstanediol**	Androstanediol
AQ733	Bottom	225.0						
	Top	661.9						
	LogEC50	-8.702						
	HillSlope	1.237						
	EC50	1.99E-09						
	amount at 10-6M	661.0						
**Plate #'s**		**Testosterone**	Testosterone	Testosterone	Testosterone	**DHT**	**Androstanediol**	Androstanediol
AQ735	Bottom							
	Top							
	LogEC50							
	HillSlope							
	EC50							
	amount at 10-6M							

	A4		A5	A6	A7	A8	A9
	Androstendione		4-Androstenediol	5-Androstenediol	epitestosterone	DHEA	Androsterone
**avg EC50 (M)**	**8.41E-08**		**1.93E-08**	**4.90E-06**			
**avg EC50 (nM)**	**84.12**		**19.34**	**4899.42**	**10,000**	**10,000**	**10,000**
**avg log EC50 for those reaching maximum**	**-7.08**		**-7.71**				
**est. log EC50 if no max activity at 10-6M**				-5.31			
**avg log EC50**	**-7.08**		**-7.71**	**7.90**	**-4.76**	**37.92**	**-7.63**
**sd log EC50**	**0.51**		**0.35**	**38.02**	**4.49**	**82.42**	**2.35**
**n log EC50**	**9**		**9**	**6**	**4**	**4**	**5**
**min log EC50**	**-7.83**		**-8.29**				
**max log EC50**	**-6.27**		**-7.17**				
sd EC50 lower range	25.71		8.56				
sd EC50 upper range	275.23		43.70				
MW	286.41		290.44	290.44	288.42	288.42	290.44
**ng/ml at EC50**	**24.09**		**5.62**	**1422.99**	**2,884**	**2,884**	**2,904**
**detectable level in 0.5u/40ul Assay**	**485.96**		**82.96**	**11383.90**	**230,736**	**230,736**	**232,352**
**Plate #'s**	**Androstendione**	Androstendione	**Androstenediol-4**	**Androstendiol-5**	**epitestosterone**	**DHEA**	**Androsterone**
AQ55 Bottom					214.5	209.4	210.1
AQ56 Top					295.3	312.4	274.1
LogEC50					-7.126	-5.801	-6.887
HillSlope					0.8295	0.4715	3.689
EC50					7.48E-08	1.58E-06	1.30E-07
amount at 10-6M					286.7	255.2	272.8
**Plate #'s**	**Androstendione**	Androstendione	**Androstenediol-4**	**Androstendiol-5**	**epitestosterone**	**DHEA**	**Androsterone**
AQ286 Bottom					345	508.7	
AQ287 Top					3.50E+07	326.3	
LogEC50					1.963	-6.455	
HillSlope					0.6652	-13.97	
EC50					91.89	3.51E-07	
amount at 10-6M					537.7	508.7	

**Plate #'s**		**Androstendione**	Androstendione	**Androstenediol-4**	**Androstendiol-5**	**epitestosterone**	**DHEA**	**Androsterone**
AQ520	Bottom					247.8	-141.9	268.4
AQ521	Top					298.9	284.5	245.4
	LogEC50					-6.692	2.546	-11.78
	HillSlope					0.977	-0.1009	
	EC50					2.03E-07	351.2	1.66E-12
	amount at 10-6M					291.0	225.9	256.5
**Plate #'s**		**Androstendione**	Androstendione	**Androstenediol-4**	**Androstendiol-5**	**epitestosterone**	**DHEA**	**Androsterone**
AQ558	Bottom						131.6	
AQ559	Top						46119	
	LogEC50						161.4	
	HillSlope						0.01587	
	EC50							
	amount at 10-6M						235.0	
**Plate #'s**		**Androstendione**	Androstendione	**Androstenediol-4**	**Androstendiol-5**	**epitestosterone**	**DHEA**	**Androsterone**
AQ578	Bottom			181.9	175.8			
AQ579	Top			938.9	300.6			
	LogEC50			-7.169	-7.632			
	HillSlope							
	EC50			6.77E-08	2.33E-08			
	amount at 10-6M			901.3	293.8			
**Plate #'s**		**Androstendione**	Androstendione	**Androstenediol-4**	**Androstendiol-5**	**epitestosterone**	**DHEA**	**Androsterone**
AQ595	Bottom	201.3						
AQ597	Top	736.7						
	LogEC50	-7.83E+00						
	HillSlope	1.119						
	EC50	1.47E-08						
	amount at 10-6M	723.5						
**Plate #'s**		**Androstendione**	Androstendione	**Androstenediol-4**	**Androstendiol-5**	**epitestosterone**	**DHEA**	**Androsterone**
AQ636	Bottom	175.5		171.4				
	Top	483.2		554.8				
	LogEC50	-7.125		-7.403				
	HillSlope							
	EC50	7.50E-08		3.96E-08				
	amount at 10-6M	459.1		545.3				

Plate #'s		Androstendione	Androstendione	Androstenediol-4	Androstendiol-5	epitestosterone	DHEA	Androsterone
AQ641	Bottom	178.8	180.8	199				
	Top	415.6	454.8	588.7				
	LogEC50	-7.377	-7.132	-8.291				
	HillSlope	1.231		1.058				
	EC50	4.20E-08	7.38E-08	5.11E-09				
	amount at 10-6M	409.6	437.9	580.7				
**Plate #'s**		**Androstendione**	Androstendione	**Androstenediol-4**	**Androstendiol-5**	**epitestosterone**	**DHEA**	**Androsterone**
AQ662	Bottom	86.54	81.68	77.74	80.95			
AQ664	Top	231.8	339.7	407.9	173.6			
	LogEC50	-6.265	-6.866	-7.624	-7.588			
	HillSlope	0.5753	0.9784					
	EC50	5.44E-07	1.36E-07	2.38E-08	2.59E-08			
	amount at 10-6M	172.0	307.6	394.5	168.7			
**Plate #'s**		**Androstendione**	Androstendione	**Androstenediol-4**	**Androstendiol-5**	**epitestosterone**	**DHEA**	**Androsterone**
AQ694	Bottom	49.4		51.27				
AQ696	Top	193.1		348.7				
	LogEC50	-7.654		-7.975				
	HillSlope	0.9031		1.767				
	EC50	2.22E-08		1.06E-08				
	amount at 10-6M	188.3		359.2				
**Plate #'s**		**Androstendione**	Androstendione	**Androstenediol-4**	**Androstendiol-5**	**epitestosterone**	**DHEA**	**Androsterone**
AQ706	Bottom							
	Top							
	LogEC50							
	HillSlope							
	EC50							
	amount at 10-6M							71.30
**Plate #'s**		**Androstendione**	Androstendione	**Androstenediol-4**	**Androstendiol-5**	**epitestosterone**	**DHEA**	**Androsterone**
AQ745	Bottom			52.15	59.99			
AQ746	Top			297.4	86.21			
	LogEC50			-7.36	85.5			
	HillSlope				0.2664			
	EC50			4.36E-08				
	amount at 10-6M			220.8	102.8			

**Plate #'s**		**Androstendione**	Androstendione	**Androstenediol-4**	**Androstendiol-5**	**epitestosterone**	**DHEA**	**Androsterone**
AQ295	Bottom	100.8						
	Top	401.3						
	LogEC50	-6.438						
	HillSlope	0.8154						
	EC50	3.65E-07						
	amount at 10-6M	310.4						
**Plate #'s**		**Androstendione**	Androstendione	**Androstenediol-4**	**Androstendiol-5**	**epitestosterone**	**DHEA**	**Androsterone**
AQ296	Bottom	102						
	Top	346.9						
	LogEC50	-6.986						
	HillSlope	0.8683						
	EC50	1.03E-07						
	amount at 10-6M	317.4						
**Plate #'s**		**Androstendione**	Androstendione	**Androstenediol-4**	**Androstendiol-5**	**epitestosterone**	**DHEA**	**Androsterone**
AQ897	Bottom					102.9		
	Top					146.3		
	LogEC50					-7.174		
	HillSlope					0.6718		
	EC50					6.70E-08		
	amount at 10-6M					139.4		
**Plate #'s**		**Androstendione**	Androstendione	**Androstenediol-4**	**Androstendiol-5**	**epitestosterone**	**DHEA**	**Androsterone**
AQ1347	Bottom							
AQ1348	Top							
AQ1349	LogEC50							
AQ1350	HillSlope							
	EC50							
	amount at 10-6M							
**Plate #'s**		**Androstendione**	Androstendione	**Androstenediol-4**	**Androstendiol-5**	**epitestosterone**	**DHEA**	**Androsterone**
AQ1355	Bottom							
AQ1356	Top							
AQ1357	LogEC50							
AQ1358	HillSlope							
	EC50							
	amount at 10-6M							

Plate #'s		Androstendione	Androstendione	Androstenediol-4	Androstendiol-5	epitestosterone	DHEA	Androsterone
AQ1360	Bottom							
AQ1361	Top							
AQ1362	LogEC50							
AQ1363	HillSlope							
	EC50							
	amount at 10-6M							

Plate #'s		Androstendione	Androstendione	Androstenediol-4	Androstendiol-5	epitestosterone	DHEA	Androsterone
AQ301	Bottom	227.6					no fit	228.5
	Top	446.9						305.7
	LogEC50	-7.236						-6.194
	HillSlope	1.000						0.725
	EC50	5.81E-08						6.40E-07
	amount at 10-6M	434.7					237.6	273.4

Plate #'s		Androstendione	Androstendione	Androstenediol-4	Androstendiol-5	epitestosterone	DHEA	Androsterone
AQ312	Bottom							
	Top							
	LogEC50							
	HillSlope							
	EC50							
	amount at 10-6M							

Plate #'s		Androstendione	Androstendione	Androstenediol-4	Androstendiol-5	epitestosterone	DHEA	Androsterone
AQ352	Bottom			185.2	191.8			
	Top			298.6	235.5			
	LogEC50			-7.887	-7.540			
	HillSlope			0.997	-1.008			
	EC50			1.30E-08	2.88E-08			
	amount at 10-6M			296.9	234.2			

Plate #'s		Androstendione	Androstendione	Androstenediol-4	Androstendiol-5	epitestosterone	DHEA	Androsterone
AQ273	Bottom	246.0					no fit	247.1
	Top	456.3						330.7
	LogEC50	-7.319						-6.199
	HillSlope	0.975						0.855
	EC50	4.79E-08						6.33E-07
	amount at 10-6M	446.5					259.0	297.0

Plate #'s		Androstendione	Androstendione	Androstenediol-4	Androstendiol-5	epitestosterone	DHEA	Androsterone
AQ327	Bottom							
	Top							
	LogEC50							
	HillSlope							
	EC50							
	amount at 10-6M							
**Plate #'s**		**Androstendione**	Androstendione	**Androstenediol-4**	**Androstendiol-5**	**epitestosterone**	**DHEA**	**Androsterone**
AQ290	Bottom			273.4	276.0			
	Top			565.2	364.5			
	LogEC50			-7.813	-7.666			
	HillSlope			1.070	1.076			
	EC50			1.54E-08	2.16E-08			
	amount at 10-6M			561.1	363.8			
**Plate #'s**		**Androstendione**	Androstendione	**Androstenediol-4**	**Androstendiol-5**	**epitestosterone**	**DHEA**	**Androsterone**
AQ711	Bottom	230.3					no fit	230.0
	Top	413.2						260.2
	LogEC50	-7.269						-7.110
	HillSlope	1.003						1.434
	EC50	5.38E-08						7.77E-08
	amount at 10-6M	403.3					236.4	259.5
**Plate #'s**		**Androstendione**	Androstendione	**Androstenediol-4**	**Androstendiol-5**	**epitestosterone**	**DHEA**	**Androsterone**
AQ733	Bottom							
	Top							
	LogEC50							
	HillSlope							
	EC50							
	amount at 10-6M							
**Plate #'s**		**Androstendione**	Androstendione	**Androstenediol-4**	**Androstendiol-5**	**epitestosterone**	**DHEA**	**Androsterone**
AQ735	Bottom			256.5	257.8			
	Top			475.8	327.5			
	LogEC50			-7.899	-7.702			
	HillSlope			1.197	1.009			
	EC50			1.26E-08	1.99E-08			
	amount at 10-6M			467.7	326.7			

	P10	P11	P12	P13	P14
	5a-pregnan-17a-ol-3,20-dione	17a-Hydroxyprogesterone	17OH-pregnenolone	pregnenolone	progesterone
**avg EC50 (M)**					**5.39E-06**
**avg EC50 (nM)**	**10,000**	**10,000**	**10,000**	**10,000**	**5389.13**
**avg log EC50 for those reaching maximum**					
**est. log EC50 if no max activity at 10-6M**					-5.27
**avg log EC50**	**-8.33**	**-14.91**	**-9.46**	**13.68**	**-6.85**
**sd log EC50**	**2.31**	**21.80**	**#DIV/0!**	**33.99**	**0.38**
**n log EC50**	**3**	**4**	**1**	**2**	**4**
**min log EC50**					
**max log EC50**					
sd EC50 lower range					
sd EC50 upper range					
MW	332.48	332.48	330.46	316.48	290.44
**ng/ml at EC50**	**3,325**	**3,325**	**3,305**	**3,165**	**1565.22**
**detectable level in 0.5u/40ul Assay**	**265,984**	**265,984**	**264,368**	**253,184**	**12521.75**

**Plate #'s**		**5a-pregnan-17a-ol-3,20-dione**	**17a-Hydroxyprogesterone**	**17OH-pregnenolone**	**pregnenolone**	**progesterone**
AQ55	Bottom					
AQ56	Top					
	LogEC50					
	HillSlope					
	EC50					
	amount at 10-6M					

**Plate #'s**		**5a-pregnan-17a-ol-3,20-dione**	**17a-Hydroxyprogesterone**	**17OH-pregnenolone**	**pregnenolone**	**progesterone**
AQ286	Bottom					
AQ287	Top					
	LogEC50					
	HillSlope					
	EC50					
	amount at 10-6M					

Plate #'s		5a-pregnan-17a-ol-3,20-dione	17a-Hydroxyprogesterone	17OH-pregnenolone	pregnenolone	progesterone
AQ520	Bottom					
AQ521	Top					
	LogEC50					
	HillSlope					
	EC50					
	amount at 10-6M					

Plate #'s		5a-pregnan-17a-ol-3,20-dione	17a-Hydroxyprogesterone	17OH-pregnenolone	pregnenolone	progesterone
AQ558	Bottom		219.5	210	-340700	214.5
AQ559	Top		227.4	239.4	220	322.5
	LogEC50		-47.01	-9.461	37.71	-7.089
	HillSlope		-1.116	16.22	-0.0999	1.156
	EC50		0	3.46E-10	5.15E+37	8.14E-08
	amount at 10-6M		282.8	222.1	205.3	317.3

Plate #'s		5a-pregnan-17a-ol-3,20-dione	17a-Hydroxyprogesterone	17OH-pregnenolone	pregnenolone	progesterone
AQ578	Bottom					
AQ579	Top					
	LogEC50					
	HillSlope					
	EC50					
	amount at 10-6M					

Plate #'s		5a-pregnan-17a-ol-3,20-dione	17a-Hydroxyprogesterone	17OH-pregnenolone	pregnenolone	progesterone
AQ595	Bottom		189.7			
AQ597	Top		655.6			
	LogEC50		1.635			
	HillSlope		0.1244			
	EC50		43.15			
	amount at 10-6M		239.5			

Plate #'s		5a-pregnan-17a-ol-3,20-dione	17a-Hydroxyprogesterone	17OH-pregnenolone	pregnenolone	progesterone
AQ636	Bottom					
	Top					
	LogEC50					
	HillSlope					
	EC50					
	amount at 10-6M					

Plate #'s		5a-pregnan-17a-ol-3,20-dione	17a-Hydroxyprogesterone	17OH-pregnenolone	pregnenolone	progesterone
AQ641	Bottom					
	Top					
	LogEC50					
	HillSlope					
	EC50					
	amount at 10-6M					

Plate #'s		5a-pregnan-17a-ol-3,20-dione	17a-Hydroxyprogesterone	17OH-pregnenolone	pregnenolone	progesterone
AQ662	Bottom		86.61			
AQ664	Top		105.3			
	LogEC50		-6.696			
	HillSlope		1.23E+00			
	EC50		2.01E-07			
	amount at 10-6M		103.0			

Plate #'s		5a-pregnan-17a-ol-3,20-dione	17a-Hydroxyprogesterone	17OH-pregnenolone	pregnenolone	progesterone
AQ694	Bottom	49.5				
AQ696	Top	174.8				
	LogEC50	-6.591				
	HillSlope	0.7441				
	EC50	2.57E-07				
	amount at 10-6M	141.4				

Plate #'s		5a-pregnan-17a-ol-3,20-dione	17a-Hydroxyprogesterone	17OH-pregnenolone	pregnenolone	progesterone
AQ706	Bottom	38.53				
	Top	51.82				
	LogEC50	-7.45				
	HillSlope	10.39				
	EC50	3.55E-08				
	amount at 10-6M	49.63				

Plate #'s		5a-pregnan-17a-ol-3,20-dione	17a-Hydroxyprogesterone	17OH-pregnenolone	pregnenolone	progesterone
AQ745	Bottom	50.15	55.5			
AQ746	Top	53.21	71.42			
	LogEC50	-10.96	-7.583			
	HillSlope	14.3	0.468			
	EC50	1.11E-11	2.61E-08			
	amount at 10-6M	57.0	70.5			

Plate #'s		5a-pregnan-17a-ol-3,20-dione	17a-Hydroxyprogesterone	17OH-pregnenolone	pregnenolone	progesterone
AQ295	Bottom					
	Top					
	LogEC50					
	HillSlope					
	EC50					
	amount at 10-6M					

Plate #'s		5a-pregnan-17a-ol-3,20-dione	17a-Hydroxyprogesterone	17OH-pregnenolone	pregnenolone	progesterone
AQ296	Bottom					
	Top					
	LogEC50					
	HillSlope					
	EC50					
	amount at 10-6M					

Plate #'s		5a-pregnan-17a-ol-3,20-dione	17a-Hydroxyprogesterone	17OH-pregnenolone	pregnenolone	progesterone
AQ897	Bottom					
	Top					
	LogEC50					
	HillSlope					
	EC50					
	amount at 10-6M					

Plate #'s		5a-pregnan-17a-ol-3,20-dione	17a-Hydroxyprogesterone	17OH-pregnenolone	pregnenolone	progesterone
AQ1347	Bottom					
AQ1348	Top					
AQ1349	LogEC50					
AQ1350	HillSlope					
	EC50					
	amount at 10-6M					

Plate #'s		5a-pregnan-17a-ol-3,20-dione	17a-Hydroxyprogesterone	17OH-pregnenolone	pregnenolone	progesterone
AQ1355	Bottom					
AQ1356	Top					
AQ1357	LogEC50					
AQ1358	HillSlope					
	EC50					
	amount at 10-6M					

Plate #'s		5a-pregnan-17a-ol-3,20-dione	17a-Hydroxyprogesterone	17OH-pregnenolone	pregnenolone	progesterone
AQ1360	Bottom					
AQ1361	Top					
AQ1362	LogEC50					
AQ1363	HillSlope					
	EC50					
	amount at 10-6M					

Plate #'s		5a-pregnan-17a-ol-3,20-dione	17a-Hydroxyprogesterone	17OH-pregnenolone	pregnenolone	progesterone
AQ301	Bottom					
	Top					
	LogEC50					
	HillSlope					
	EC50					
	amount at 10-6M					

Plate #'s		5a-pregnan-17a-ol-3,20-dione	17a-Hydroxyprogesterone	17OH-pregnenolone	pregnenolone	progesterone
AQ312	Bottom	no fit				
	Top					
	LogEC50					
	HillSlope					
	EC50					
	amount at 10-6M	238.6				

Plate #'s		5a-pregnan-17a-ol-3,20-dione	17a-Hydroxyprogesterone	17OH-pregnenolone	pregnenolone	progesterone
AQ352	Bottom		no fit	no fit	no fit	189.8
	Top					222.1
	LogEC50					-6.553
	HillSlope					0.877
	EC50					2.80E-07
	amount at 10-6M		197.4	195.6	200.2	214.1

Plate #'s		5a-pregnan-17a-ol-3,20-dione	17a-Hydroxyprogesterone	17OH-pregnenolone	pregnenolone	progesterone
AQ273	Bottom					
	Top					
	LogEC50					
	HillSlope					
	EC50					
	amount at 10-6M					

**Plate #'s**		**5a-pregnan-17a-ol-3,20-dione**	**17a-Hydroxyprogesterone**	**17OH-pregnenolone**	**pregnenolone**	**progesterone**
AQ327	Bottom	no fit				
	Top					
	LogEC50					
	HillSlope					
	EC50					
	amount at 10-6M	281.4				
**Plate #'s**		**5a-pregnan-17a-ol-3,20-dione**	**17a-Hydroxyprogesterone**	**17OH-pregnenolone**	**pregnenolone**	**progesterone**
AQ290	Bottom		no fit	no fit	269.4	272.7
	Top				277.9	340.6
	LogEC50				-10.360	-7.251
	HillSlope				3.142	0.971
	EC50				4.39E-11	5.61E-08
	amount at 10-6M		290.2	283.1	298.7	337.9
**Plate #'s**		**5a-pregnan-17a-ol-3,20-dione**	**17a-Hydroxyprogesterone**	**17OH-pregnenolone**	**pregnenolone**	**progesterone**
AQ711	Bottom					
	Top					
	LogEC50					
	HillSlope					
	EC50					
	amount at 10-6M					
**Plate #'s**		**5a-pregnan-17a-ol-3,20-dione**	**17a-Hydroxyprogesterone**	**17OH-pregnenolone**	**pregnenolone**	**progesterone**
AQ733	Bottom	no fit				
	Top					
	LogEC50					
	HillSlope					
	EC50					
	amount at 10-6M	237.1				
**Plate #'s**		**5a-pregnan-17a-ol-3,20-dione**	**17a-Hydroxyprogesterone**	**17OH-pregnenolone**	**pregnenolone**	**progesterone**
AQ735	Bottom		no fit	no fit	no fit	254.6
	Top					318.4
	LogEC50					-6.497
	HillSlope					0.6298
	EC50					3.19E-07
	amount at 10-6M		272.1	253.7	251.7	297.6

	C15	C16	C17	C18	C19	E20	E21	E22
	**11-dexoycorticosterone**	**11-deoxycortisol**	**Corticosterone**	**Aldosterone**	**Cortisol**	**Estradiol**	**Estrone**	**Estriol**
**avg EC50 (M)**						**1.69E-06**		
**avg EC50 (nM)**	**10,000**	**10,000**	**10,000**	**10,000**	**10,000**	**1686.61**	**10,000**	**10,000**
**avg log EC50 for those reaching maximum**								
**est. log EC50 if no max activity at 10-6M**						-5.77		
**avg log EC50**	**-7.26**	**-7.77**	**-7.57**	**-7.41**	**-7.47**	**-4.82**	**-7.44**	**-11.52**
**sd log EC50**	**0.14**	**0.88**	**0.58**	**0.43**	**2.16**	**3.48**	**0.53**	**3.76**
**n log EC50**	**3**	**4**	**4**	**4**	**2**	**7**	**4**	**6**
**min log EC50**								
**max log EC50**								
sd EC50 lower range								
sd EC50 upper range								
MW	330.46	346.46	346.46	360.44	362.46	272.38	270.37	288.38
**ng/ml at EC50**	**3,305**	**3,465**	**3,465**	**3,604**	**3,625**	**459.40**	**2,704**	**2,884**
**detectable level in 0.5u/40ul Assay**	**264,368**	**277,168**	**277,168**	**288,352**	**289,968**	**3675.19**	**216,296**	**230,704**

**Plate #'s**		**11-dexoycorticosterone**	**11-deoxycortisol**	**Corticosterone**	**Aldosterone**	**Cortisol**	**Estradiol**	**Estrone**	**Estriol**
AQ55	Bottom								
AQ56	Top								
	LogEC50								
	HillSlope								
	EC50								
	amount at 10-6M								

**Plate #'s**		**11-dexoycorticosterone**	**11-deoxycortisol**	**Corticosterone**	**Aldosterone**	**Cortisol**	**Estradiol**	**Estrone**	**Estriol**
AQ286	Bottom								
AQ287	Top								
	LogEC50								
	HillSlope								
	EC50								
	amount at 10-6M								

Plate #'s		11-dexoycorticosterone	11-deoxycortisol	Corticosterone	Aldosterone	Cortisol	Estradiol	Estrone	Estriol
AQ520	Bottom								
AQ521	Top								
	LogEC50								
	HillSlope								
	EC50								
	amount at 10-6M								

Plate #'s		11-dexoycorticosterone	11-deoxycortisol	Corticosterone	Aldosterone	Cortisol	Estradiol	Estrone	Estriol
AQ558	Bottom	218.8	207.8	224.8	231.4	213.6			
AQ559	Top	273.4	241.8	271.4	266.4	236.7			
	LogEC50	-7.42	-8.838	-7.453	-7.285	-8.998			
	HillSlope	9.54	0.3967	2.232	3.51				
	EC50	3.80E-08	1.45E-09	3.52E-08	5.19E-08	1.01E-09			
	amount at 10-6M	272.2	237.8	265.6	266.3	230.7			

Plate #'s		11-dexoycorticosterone	11-deoxycortisol	Corticosterone	Aldosterone	Cortisol	Estradiol	Estrone	Estriol
AQ578	Bottom						172.8	184.2	
AQ579	Top						10295	241	
	LogEC50						-2.6	-8.18	
	HillSlope						0.3811	0.7744	
	EC50						0.002514	6.61E-09	
	amount at 10-6M						664.1	241.3	

Plate #'s		11-dexoycorticosterone	11-deoxycortisol	Corticosterone	Aldosterone	Cortisol	Estradiol	Estrone	Estriol
AQ595	Bottom								
AQ597	Top								
	LogEC50								
	HillSlope								
	EC50								
	amount at 10-6M								

Plate #'s		11-dexoycorticosterone	11-deoxycortisol	Corticosterone	Aldosterone	Cortisol	Estradiol	Estrone	Estriol
AQ636	Bottom								
	Top								
	LogEC50								
	HillSlope								
	EC50								
	amount at 10-6M								

Plate #'s		11-dexoycorticosterone	11-deoxycortisol	Corticosterone	Aldosterone	Cortisol	Estradiol	Estrone	Estriol
AQ641	Bottom								
	Top								
	LogEC50								
	HillSlope								
	EC50								
	amount at 10-6M								

Plate #'s		11-dexoycorticosterone	11-deoxycortisol	Corticosterone	Aldosterone	Cortisol	Estradiol	Estrone	Estriol
AQ662	Bottom								
AQ664	Top								
	LogEC50								
	HillSlope								
	EC50								
	amount at 10-6M								

Plate #'s		11-dexoycorticosterone	11-deoxycortisol	Corticosterone	Aldosterone	Cortisol	Estradiol	Estrone	Estriol
AQ694	Bottom								
AQ696	Top								
	LogEC50								
	HillSlope								
	EC50								
	amount at 10-6M								

Plate #'s		11-dexoycorticosterone	11-deoxycortisol	Corticosterone	Aldosterone	Cortisol	Estradiol	Estrone	Estriol
AQ706	Bottom				41.44				39.12
	Top				72.20				41.13
	LogEC50				-7.212				-8.923
	HillSlope				1.314				-22.97
	EC50				6.14E-08				1.20E-09
	amount at 10-6M								46.80

Plate #'s		11-dexoycorticosterone	11-deoxycortisol	Corticosterone	Aldosterone	Cortisol	Estradiol	Estrone	Estriol
AQ745	Bottom								63.56
AQ746	Top								57.56
	LogEC50								-9.252
	HillSlope								-21.65
	EC50								5.60E-10
	amount at 10-6M								56.7

Plate #'s		11-dexoycorticosterone	11-deoxycortisol	Corticosterone	Aldosterone	Cortisol	Estradiol	Estrone	Estriol
AQ295	Bottom								
	Top								
	LogEC50								
	HillSlope								
	EC50								
	amount at 10-6M								
**Plate #'s**		**11-dexoycorticosterone**	**11-deoxycortisol**	**Corticosterone**	**Aldosterone**	**Cortisol**	**Estradiol**	**Estrone**	**Estriol**
AQ296	Bottom								
	Top								
	LogEC50								
	HillSlope								
	EC50								
	amount at 10-6M								
**Plate #'s**		**11-dexoycorticosterone**	**11-deoxycortisol**	**Corticosterone**	**Aldosterone**	**Cortisol**	**Estradiol**	**Estrone**	**Estriol**
AQ897	Bottom								
	Top								
	LogEC50								
	HillSlope								
	EC50								
	amount at 10-6M								
**Plate #'s**		**11-dexoycorticosterone**	**11-deoxycortisol**	**Corticosterone**	**Aldosterone**	**Cortisol**	**Estradiol**	**Estrone**	**Estriol**
AQ1347	Bottom						129.9		-345800.0
AQ1348	Top						219.4		103.6
AQ1349	LogEC50						-7.497		-18.110
AQ1350	HillSlope						0.991		0.000
	EC50						3.18E-08		7.69E-19
	amount at 10-6M						217.7		107.5
**Plate #'s**		**11-dexoycorticosterone**	**11-deoxycortisol**	**Corticosterone**	**Aldosterone**	**Cortisol**	**Estradiol**	**Estrone**	**Estriol**
AQ1355	Bottom						77.8		54.7
AQ1356	Top						10028.0		100.6
AQ1357	LogEC50						0.656		-8.203
AQ1358	HillSlope						0.219		0.106
	EC50						4.53E+00		6.27E-09
	amount at 10-6M						430.4		87.9

Plate #'s		11-dexoycorticosterone	11-deoxycortisol	Corticosterone	Aldosterone	Cortisol	Estradiol	Estrone	Estriol
AQ1360	Bottom						97.3		73.8
AQ1361	Top						22065.0		87.3
AQ1362	LogEC50						-1.805		-11.070
AQ1363	HillSlope						0.408		-1.289
	EC50						1.57E-02		8.47E-12
	amount at 10-6M						522.7		75.9
**Plate #'s**		**11-dexoycorticosterone**	**11-deoxycortisol**	**Corticosterone**	**Aldosterone**	**Cortisol**	**Estradiol**	**Estrone**	**Estriol**
AQ301	Bottom				no fit				
	Top								
	LogEC50								
	HillSlope								
	EC50								
	amount at 10-6M				231.2				
**Plate #'s**		**11-dexoycorticosterone**	**11-deoxycortisol**	**Corticosterone**	**Aldosterone**	**Cortisol**	**Estradiol**	**Estrone**	**Estriol**
AQ312	Bottom		233.3	224.9		no fit	237.4	229.2	no fit
	Top		267.3	255.2			361.8	264.8	
	LogEC50		-8.083	-8.239			-7.593	-6.971	
	HillSlope		0.365	0.360			0.704	1.097	
	EC50		8.26E-09	5.77E-09			2.55E-08	1.07E-07	
	amount at 10-6M		262.4	251.9		227.6	357.2	261.8	233.7
**Plate #'s**		**11-dexoycorticosterone**	**11-deoxycortisol**	**Corticosterone**	**Aldosterone**	**Cortisol**	**Estradiol**	**Estrone**	**Estriol**
AQ352	Bottom	183.0							
	Top	198.6							
	LogEC50	-7.158							
	HillSlope	0.923							
	EC50	6.95E-08							
	amount at 10-6M	198.0							
**Plate #'s**		**11-dexoycorticosterone**	**11-deoxycortisol**	**Corticosterone**	**Aldosterone**	**Cortisol**	**Estradiol**	**Estrone**	**Estriol**
AQ273	Bottom				243.2				
	Top				265.9				
	LogEC50				-7.116				
	HillSlope				1.464				
	EC50				7.66E-08				
	amount at 10-6M				265.6				

Plate #'s		11-dexoycorticosterone	11-deoxycortisol	Corticosterone	Aldosterone	Cortisol	Estradiol	Estrone	Estriol
AQ327	Bottom		274.9	271.3		no fit	278.7	271.8	272.3
	Top		296.1	301.7			440.3	292.3	285.0
	LogEC50		-7.304	-7.759			-7.433	-7.172	-13.540
	HillSlope		2.728	1.057			0.769	6.119	-1.562
	EC50		4.97E-08	1.74E-08			3.69E-08	6.73E-08	2.84E-14
	amount at 10-6M		296.1	299.6		280.4	429.4	292.3	274.0
**Plate #'s**		**11-dexoycorticosterone**	**11-deoxycortisol**	**Corticosterone**	**Aldosterone**	**Cortisol**	**Estradiol**	**Estrone**	**Estriol**
AQ290	Bottom	no fit							
	Top								
	LogEC50								
	HillSlope								
	EC50								
	amount at 10-6M	326.1							
**Plate #'s**		**11-dexoycorticosterone**	**11-deoxycortisol**	**Corticosterone**	**Aldosterone**	**Cortisol**	**Estradiol**	**Estrone**	**Estriol**
AQ711	Bottom				238.8				
	Top				229.4				
	LogEC50				-8.046				
	HillSlope				-0.847				
	EC50				8.99E-09				
	amount at 10-6M				236.8				
**Plate #'s**		**11-dexoycorticosterone**	**11-deoxycortisol**	**Corticosterone**	**Aldosterone**	**Cortisol**	**Estradiol**	**Estrone**	**Estriol**
AQ733	Bottom		235.1	222.1		224.2		223.5	no fit
	Top		259.0	261.5		268.7		231.8	
	LogEC50		-6.843	-6.845		-5.948		-7.431	
	HillSlope		1.293	0.530		0.418		1.330	
	EC50		1.44E-07	1.43E-07		1.13E-06		3.71E-08	
	amount at 10-6M		257.4	251.4		246.0		231.6	227.7
**Plate #'s**		**11-dexoycorticosterone**	**11-deoxycortisol**	**Corticosterone**	**Aldosterone**	**Cortisol**	**Estradiol**	**Estrone**	**Estriol**
AQ735	Bottom	255.2					254.0		
	Top	294.7					356.6		
	LogEC50	-7.191					-7.457		
	HillSlope	0.9899					0.6814		
	EC50	6.44E-08					3.50E-08		
	amount at 10-6M	292.9					347.5		

	23	AAS24	AAS25	AAS26	AAS27	AAS28	AAS29	AAS30	AAS31
	**Cholesterol**	**THG**	**mibolerone**	**mestanolone**	**mesterolone**	**normethandrone**	**gestrinone**	**nandrolone**	**trenbolone**
**avg EC50 (M)**		**5.09E-10**	**1.41E-10**	**3.74E-10**	**9.20E-10**	**3.40E-10**	**2.09E-09**	**2.51E-09**	**6.29E-10**
**avg EC50 (nM)**	**10,000**	**0.51**	**0.14**	**0.37**	**0.92**	**0.34**	**2.09**	**2.51**	**0.63**
**avg log EC50 for those reaching maximum**		**-9.29**	**-9.85**	**-9.43**	**-9.04**	**-9.47**	**-8.68**	**-8.60**	**-9.20**
**est. log EC50 if no max activity at 10-6M**									
**avg log EC50**	**-7.27**	**-9.29**	**-9.85**	**-9.43**	**-9.04**	**-9.47**	**-8.68**	**-8.60**	**-9.20**
**sd log EC50**	**8.70**	**0.01**	**0.05**	**0.67**	**0.81**	**0.12**	**0.80**	**0.96**	**0.36**
**n log EC50**	**2**	**2**	**2**	**7**	**8**	**2**	**4**	**7**	**7**
**min log EC50**		**-9.30**	**-9.89**	**-10.77**	**-10.25**	**-9.56**	**-9.86**	**-9.59**	**-9.77**
**max log EC50**		**-9.29**	**-9.81**	**-8.67**	**-7.81**	**-9.38**	**-8.10**	**-7.17**	**-8.74**
sd EC50 lower range		0.50	0.12	0.08	0.14	0.26	0.33	0.28	0.28
sd EC50 upper range		0.52	0.16	1.77	5.96	0.45	13.21	22.88	1.43
MW	386.65	312.45	302.45	304.47	304.47	288.42	308.41	274.40	270.37
**ng/ml at EC50**	**3,867**	**0.16**	**0.04**	**0.11**	**0.28**	**0.10**	**0.64**	**0.69**	**0.17**
**detectable level in 0.5u/40ul Assay**	**309,320**	**1.16**	**0.32**	**0.89**	**2.20**	**0.78**	**5.22**	**5.60**	**1.41**
**Plate #'s**	**Cholesterol**	**THG**	**mibolerone**	**mestanolone**	**mesterolone**	**normethandrone**	**gestrinone**	**nandrolone**	**trenbolone**
AQ55 Bottom							171.3	174.2	
AQ56 Top							947.3	958.9	
LogEC50							-8.096	-7.764	
HillSlope							1.277	1.408	
EC50							8.01E-09	1.72E-08	
amount at 10-6M							983.9	966.7	
**Plate #'s**	**Cholesterol**	**THG**	**mibolerone**	**mestanolone**	**mesterolone**	**normethandrone**	**gestrinone**	**nandrolone**	**trenbolone**
AQ286 Bottom					308.1		301.4	317.4	
AQ287 Top					1315		1108	1317	
LogEC50					-8.769		-8.417	-7.171	
HillSlope					1.485		1.57	3.494	
EC50					1.70E-09		3.83E-09	6.75E-08	
amount at 10-6M					1398.7		1010.3	1206.3	

Plate #'s		Cholesterol	THG	mibolerone	mestanolone	mesterolone	normethandrone	gestrinone	nandrolone	trenbolone
AQ520	Bottom					222.5		209.8	216.9	
AQ521	Top					1192		1104	1182	
	LogEC50					-7.81		-8.344	-7.923	
	HillSlope					1.39		1.147	1.407	
	EC50					1.55E-08		4.53E-09	1.19E-08	
	amount at 10-6M					1217.7		1161.1	1217.2	

Plate #'s		Cholesterol	THG	mibolerone	mestanolone	mesterolone	normethandrone	gestrinone	nandrolone	trenbolone
AQ558	Bottom	226.7								
AQ559	Top	239								
	LogEC50	-1.115								
	HillSlope	9.267								
	EC50	0.07678								
	amount at 10-6M	261.8								

Plate #'s		Cholesterol	THG	mibolerone	mestanolone	mesterolone	normethandrone	gestrinone	nandrolone	trenbolone
AQ578	Bottom									
AQ579	Top									
	LogEC50									
	HillSlope									
	EC50									
	amount at 10-6M									

Plate #'s		Cholesterol	THG	mibolerone	mestanolone	mesterolone	normethandrone	gestrinone	nandrolone	trenbolone
AQ595	Bottom				200.4	257.4			204.7	189.1
AQ597	Top				1731	1692			1651	1495
	LogEC50				-9.532	-8.563			-9.22	-9.09E+00
	HillSlope				1.457	11.88			1.929	1.493
	EC50				2.94E-10	2.73E-09			6.02E-10	8.15E-10
	amount at 10-6M				1802.4	1801.6			1739.3	1484.3

Plate #'s		Cholesterol	THG	mibolerone	mestanolone	mesterolone	normethandrone	gestrinone	nandrolone	trenbolone
AQ636	Bottom									
	Top									
	LogEC50									
	HillSlope									
	EC50									
	amount at 10-6M									

Plate #'s		Cholesterol	THG	mibolerone	mestanolone	mesterolone	normethandrone	gestrinone	nandrolone	trenbolone
AQ641	Bottom									
	Top									
	LogEC50									
	HillSlope									
	EC50									
	amount at 10-6M									
**Plate #'s**		**Cholesterol**	**THG**	**mibolerone**	**mestanolone**	**mesterolone**	**normethandrone**	**gestrinone**	**nandrolone**	**trenbolone**
AQ662	Bottom				100.9	91.32				102.6
AQ664	Top				795	828				742.5
	LogEC50				-8.901	-8.497				-9.009
	HillSlope				0.8259	1.348				1.519
	EC50				1.26E-09	3.19E-09				9.79E-10
	amount at 10-6M				829.7	844.4				761.8
**Plate #'s**		**Cholesterol**	**THG**	**mibolerone**	**mestanolone**	**mesterolone**	**normethandrone**	**gestrinone**	**nandrolone**	**trenbolone**
AQ694	Bottom				48.84	49.74				50.67
AQ696	Top				654.4	671.1				653.8
	LogEC50				-9.439	-8.9330				-9.108
	HillSlope				1.298	1.362				1.514
	EC50				3.64E-10	1.17E-09				7.80E-10
	amount at 10-6M				673.5	699.3				681.3
**Plate #'s**		**Cholesterol**	**THG**	**mibolerone**	**mestanolone**	**mesterolone**	**normethandrone**	**gestrinone**	**nandrolone**	**trenbolone**
AQ706	Bottom							42.98		
	Top							376.30		
	LogEC50							-9.864		
	HillSlope							0.5989		
	EC50							1.37E-10		
	amount at 10-6M							406.13		
**Plate #'s**		**Cholesterol**	**THG**	**mibolerone**	**mestanolone**	**mesterolone**	**normethandrone**	**gestrinone**	**nandrolone**	**trenbolone**
AQ745	Bottom				54.32	72.92				63.97
AQ746	Top				542.7	561.3				522.9
	LogEC50				-9.193	-9.908				-8.742
	HillSlope				1.534	0.6565				1.792
	EC50				6.41E-10	1.24E-10				1.81E-09
	amount at 10-6M				561.9	587.7				547.2

Plate #'s		Cholesterol	THG	mibolerone	mestanolone	mesterolone	normethandrone	gestrinone	nandrolone	trenbolone
AQ295	Bottom		118.9	101.7			108.7			
	Top		525.1	473.8			459.9			
	LogEC50		-9.287	-9.889			-9.555			
	HillSlope		1.215	1.604			1.228			
	EC50		5.16E-10	1.29E-10			2.78E-10			
	amount at 10-6M		536.4	493.3			483.7			

Plate #'s		Cholesterol	THG	mibolerone	mestanolone	mesterolone	normethandrone	gestrinone	nandrolone	trenbolone
AQ296	Bottom		109.8	113.8			103.2			
	Top		457.1	498.1			459.9			
	LogEC50		-9.3	-9.812			-9.382			
	HillSlope		0.9577	1.385			1.168			
	EC50		5.01E-10	1.54E-10			4.15E-10			
	amount at 10-6M		466.1	530.6			474.3			

Plate #'s		Cholesterol	THG	mibolerone	mestanolone	mesterolone	normethandrone	gestrinone	nandrolone	trenbolone
AQ897	Bottom									
	Top									
	LogEC50									
	HillSlope									
	EC50									
	amount at 10-6M									

Plate #'s		Cholesterol	THG	mibolerone	mestanolone	mesterolone	normethandrone	gestrinone	nandrolone	trenbolone
AQ1347	Bottom				138.2	121.1			139.7	136.9
AQ1348	Top				624.0	564.8			657.7	668.9
AQ1349	LogEC50				-10.770	-10.250			-9.074	-9.089
AQ1350	HillSlope				0.997	0.748			1.406	1.884
	EC50				1.70E-11	5.57E-11			8.44E-10	8.14E-10
	amount at 10-6M				634.7	558.1			636.1	652.8

Plate #'s		Cholesterol	THG	mibolerone	mestanolone	mesterolone	normethandrone	gestrinone	nandrolone	trenbolone
AQ1355	Bottom				61.3				82.4	84.0
AQ1356	Top				785.7				687.4	696.8
AQ1357	LogEC50				-8.665				-9.585	-9.772
AQ1358	HillSlope				0.985				1.189	1.345
	EC50				2.17E-09				2.60E-10	1.69E-10
	amount at 10-6M				772.7				680.6	707.7

**Plate #'s**		**Cholesterol**	**THG**	**mibolerone**	**mestanolone**	**mesterolone**	**normethandrone**	**gestrinone**	**nandrolone**	**trenbolone**
AQ1360	Bottom				99.8	559.0			100.9	77.9
AQ1361	Top				825.3	795.3			746.8	769.3
AQ1362	LogEC50				-9.488	-9.559			-9.465	-9.601
AQ1363	HillSlope				0.917	19.540			1.119	1.143
	EC50				3.25E-10	2.76E-10			3.42E-10	2.51E-10
	amount at 10-6M				824.2	747.7			704.9	774.9
**Plate #'s**		**Cholesterol**	**THG**	**mibolerone**	**mestanolone**	**mesterolone**	**normethandrone**	**gestrinone**	**nandrolone**	**trenbolone**
AQ301	Bottom	no fit								
	Top									
	LogEC50									
	HillSlope									
	EC50									
	amount at 10-6M	229.5								
**Plate #'s**		**Cholesterol**	**THG**	**mibolerone**	**mestanolone**	**mesterolone**	**normethandrone**	**gestrinone**	**nandrolone**	**trenbolone**
AQ312	Bottom									
	Top									
	LogEC50									
	HillSlope									
	EC50									
	amount at 10-6M									
**Plate #'s**		**Cholesterol**	**THG**	**mibolerone**	**mestanolone**	**mesterolone**	**normethandrone**	**gestrinone**	**nandrolone**	**trenbolone**
AQ352	Bottom									
	Top									
	LogEC50									
	HillSlope									
	EC50									
	amount at 10-6M									
**Plate #'s**		**Cholesterol**	**THG**	**mibolerone**	**mestanolone**	**mesterolone**	**normethandrone**	**gestrinone**	**nandrolone**	**trenbolone**
AQ273	Bottom	no fit								
	Top									
	LogEC50									
	HillSlope									
	EC50									
	amount at 10-6M	270.8								

Plate #'s		Cholesterol	THG	mibolerone	mestanolone	mesterolone	normethandrone	gestrinone	nandrolone	trenbolone
AQ327	Bottom									
	Top									
	LogEC50									
	HillSlope									
	EC50									
	amount at 10-6M									
**Plate #'s**		**Cholesterol**	**THG**	**mibolerone**	**mestanolone**	**mesterolone**	**normethandrone**	**gestrinone**	**nandrolone**	**trenbolone**
AQ290	Bottom									
	Top									
	LogEC50									
	HillSlope									
	EC50									
	amount at 10-6M									
**Plate #'s**		**Cholesterol**	**THG**	**mibolerone**	**mestanolone**	**mesterolone**	**normethandrone**	**gestrinone**	**nandrolone**	**trenbolone**
AQ711	Bottom									
	Top									
	LogEC50									
	HillSlope									
	EC50									
	amount at 10-6M									
**Plate #'s**		**Cholesterol**	**THG**	**mibolerone**	**mestanolone**	**mesterolone**	**normethandrone**	**gestrinone**	**nandrolone**	**trenbolone**
AQ733	Bottom	222.3								
	Top	227.6								
	LogEC50	-13.420								
	HillSlope	-1.289								
	EC50	3.73E-14								
	amount at 10-6M	224.4								
**Plate #'s**		**Cholesterol**	**THG**	**mibolerone**	**mestanolone**	**mesterolone**	**normethandrone**	**gestrinone**	**nandrolone**	**trenbolone**
AQ735	Bottom									
	Top									
	LogEC50									
	HillSlope									
	EC50									
	amount at 10-6M									

	AAS32	AAS33	AAS34	AAS35	AAS36	AAS37	AAS38	AAS39
	**tibolone**	**methyltestosterone**	**stanozolol**	**fluoxymesterone**	**oxymethalone**	**danazol**	**19-norandrostenedione**	**boldione**
**avg EC50 (M)**	**1.29E-09**	**9.88E-10**	**2.10E-09**	**6.38E-09**	**4.78E-09**	**1.24E-08**	**1.24E-06**	**3.12E-06**
**avg EC50 (nM)**	**1.29**	**0.99**	**2.10**	**6.38**	**4.78**	**12.40**	**1238.02**	**3122.91**
**avg log EC50 for those reaching maximum**	**-8.89**	**-9.01**	**-8.68**	**-8.20**	**-8.32**	**-7.91**		
**est. log EC50 if no max activity at 10-6M**							-5.91	-5.51
**avg log EC50**	**-8.89**	**-9.01**	**-8.68**	**-8.20**	**-8.32**	**-7.91**	**-6.33**	**-5.46**
**sd log EC50**	**0.22**	**0.48**	**0.40**	**0.31**	**0.15**	**0.38**	**0.32**	**1.56**
**n log EC50**	**4**	**7**	**5**	**6**	**2**	**7**	**4**	**7**
**min log EC50**	**-9.08**	**-10.03**	**-9.13**	**-8.57**	**-8.42**	**-8.44**		
**max log EC50**	**-8.57**	**-8.52**	**-8.13**	**-7.77**	**-8.22**	**-7.48**		
sd EC50 lower range	0.77	0.32	0.83	3.12	3.41	5.16		
sd EC50 upper range	2.17	3.01	5.31	13.02	6.70	29.75		
MW	312.45	302.45	328.49	336.44	332.48	337.46	272.38	284.39
**ng/ml at EC50**	**0.40**	**0.30**	**0.69**	**2.15**	**1.59**	**4.18**	**337.21**	**888.13**
**detectable level in 0.5u/40ul Assay**	**3.37**	**2.60**	**6.29**	**20.68**	**16.53**	**49.55**	**2697.69**	**7105.00**
**Plate #'s**	**tibolone**	**methyltestosterone**	**stanozolol**	**fluoxymesterone**	**oxymethalone**	**danazol**	**19-norandrostenedione**	**boldione**
AQ55 Bottom						172.8	174.9	172.6
AQ56 Top						685	628.1	4042
LogEC50						-7.624	-6.625	-3.478
HillSlope						1.041	1.289	0.5201
EC50						2.38E-08	2.37E-07	0.0003324
amount at 10-6M						676.3	565.9	356.2
**Plate #'s**	**tibolone**	**methyltestosterone**	**stanozolol**	**fluoxymesterone**	**oxymethalone**	**danazol**	**19-norandrostenedione**	**boldione**
AQ286 Bottom			327			303.1	275.4	305.3
AQ287 Top			1211			984.9	1946	5111
LogEC50			-8.406			-7.479	-5.881	-3.985
HillSlope			1.001			1.31	0.879	0.5608
EC50			3.93E-09			3.32E-08	1.32E-06	1.04E-04
amount at 10-6M			1292.7			897.0	803.0	658.3

Plate #'s		tibolone	methyltestosterone	stanozolol	fluoxymesterone	oxymethalone	danazol	19-norandrostenedione	boldione
AQ520	Bottom			252.7			214.4	224	227.9
AQ521	Top			1139			838.2	829.2	464.9
	LogEC50			-8.13			-7.642	-6.36	-6.381
	HillSlope			1.509			1.002	0.8687	
	EC50			7.41E-09			2.28E-08	4.36E-07	4.16E-07
	amount at 10-6M			1177.5			837.4	631.4	398.4

Plate #'s		tibolone	methyltestosterone	stanozolol	fluoxymesterone	oxymethalone	danazol	19-norandrostenedione	boldione
AQ558	Bottom								
AQ559	Top								
	LogEC50								
	HillSlope								
	EC50								
	amount at 10-6M								

Plate #'s		tibolone	methyltestosterone	stanozolol	fluoxymesterone	oxymethalone	danazol	19-norandrostenedione	boldione
AQ578	Bottom				177.8				
AQ579	Top				931.6				
	LogEC50				-8.566				
	HillSlope				9.88E-01				
	EC50				2.72E-09				
	amount at 10-6M				940.5				

Plate #'s		tibolone	methyltestosterone	stanozolol	fluoxymesterone	oxymethalone	danazol	19-norandrostenedione	boldione
AQ595	Bottom	213.9	211.6				193.4		
AQ597	Top	1668	1579				1129		
	LogEC50	-8.978	-9.064				-8.07E+00		
	HillSlope	1.149	1.506				1.106		
	EC50	1.05E-09	8.63E-10				8.46E-09		
	amount at 10-6M	1706.6	1692.9				1201.5		

Plate #'s		tibolone	methyltestosterone	stanozolol	fluoxymesterone	oxymethalone	danazol	19-norandrostenedione	boldione
AQ636	Bottom								
	Top								
	LogEC50								
	HillSlope								
	EC50								
	amount at 10-6M								

Plate #'s		tibolone	methyltestosterone	stanozolol	fluoxymesterone	oxymethalone	danazol	19-norandrostenedione	boldione
AQ641	Bottom								
	Top								
	LogEC50								
	HillSlope								
	EC50								
	amount at 10-6M								
**Plate #'s**		**tibolone**	**methyltestosterone**	**stanozolol**	**fluoxymesterone**	**oxymethalone**	**danazol**	**19-norandrostenedione**	**boldione**
AQ662	Bottom		110.8		98.18				
AQ664	Top		688.1		595.9				
	LogEC50		-8.869		-8.016				
	HillSlope		8.75E-01		1.03E+00				
	EC50		1.35E-09		9.64E-09				
	amount at 10-6M		662.8		572.7				
**Plate #'s**		**tibolone**	**methyltestosterone**	**stanozolol**	**fluoxymesterone**	**oxymethalone**	**danazol**	**19-norandrostenedione**	**boldione**
AQ694	Bottom		48.93		55.25				
AQ696	Top		617.5		543.5				
	LogEC50		-8.944		-8.294				
	HillSlope		1.295		1.171				
	EC50		1.14E-09		5.08E-09				
	amount at 10-6M		636.0		526.6				
**Plate #'s**		**tibolone**	**methyltestosterone**	**stanozolol**	**fluoxymesterone**	**oxymethalone**	**danazol**	**19-norandrostenedione**	**boldione**
AQ706	Bottom				39.38				38.12
	Top				307.20				137.7
	LogEC50				-8.501				-7.209
	HillSlope				0.9274				
	EC50				3.16E-09				6.18E-08
	amount at 10-6M				303.63				129.60
**Plate #'s**		**tibolone**	**methyltestosterone**	**stanozolol**	**fluoxymesterone**	**oxymethalone**	**danazol**	**19-norandrostenedione**	**boldione**
AQ745	Bottom		50.39		57.07				
AQ746	Top		506		396				
	LogEC50		-8.523		-7.774				
	HillSlope		1.388		1.261				
	EC50		3.00E-09		1.68E-08				
	amount at 10-6M		520.0		393.0				

**Plate #'s**		**tibolone**	**methyltestosterone**	**stanozolol**	**fluoxymesterone**	**oxymethalone**	**danazol**	**19-norandrostenedione**	**boldione**
AQ295	Bottom					98.48			
	Top					396.3			
	LogEC50					-8.217			
	HillSlope					0.8715			
	EC50					6.07E-09			
	amount at 10-6M					392.6			
**Plate #'s**		**tibolone**	**methyltestosterone**	**stanozolol**	**fluoxymesterone**	**oxymethalone**	**danazol**	**19-norandrostenedione**	**boldione**
AQ296	Bottom					116.6			
	Top					357.8			
	LogEC50					-8.424			
	HillSlope					1.258			
	EC50					3.77E-09			
	amount at 10-6M					362.8			
**Plate #'s**		**tibolone**	**methyltestosterone**	**stanozolol**	**fluoxymesterone**	**oxymethalone**	**danazol**	**19-norandrostenedione**	**boldione**
AQ897	Bottom								
	Top								
	LogEC50								
	HillSlope								
	EC50								
	amount at 10-6M								
**Plate #'s**		**tibolone**	**methyltestosterone**	**stanozolol**	**fluoxymesterone**	**oxymethalone**	**danazol**	**19-norandrostenedione**	**boldione**
AQ1347	Bottom	152.5	122.8	123.3	126.8		139.8	132.5	117.1
AQ1348	Top	630.5	588.2	510.7	573.4		514.6	441.4	1905.0
AQ1349	LogEC50	-9.081	-10.030	-9.132	-8.021		-7.741	-6.460	-4.037
AQ1350	HillSlope	0.939	0.803	0.579	1.080		0.438		0.429
	EC50	8.31E-10	9.30E-11	7.39E-10	9.53E-09		1.82E-08	3.469E-07	9.17E-05
	amount at 10-6M	497.9	627.4	502.3	554.4		453.7	354.3	342.2
**Plate #'s**		**tibolone**	**methyltestosterone**	**stanozolol**	**fluoxymesterone**	**oxymethalone**	**danazol**	**19-norandrostenedione**	**boldione**
AQ1355	Bottom	79.5	79.9	85.6			95.3		77.8
AQ1356	Top	669.7	641.4	621.9			516.6		181.0
AQ1357	LogEC50	-8.925	-8.894	-8.823			-8.442		-6.614
AQ1358	HillSlope	0.909	1.321	0.540			0.715		
	EC50	1.19E-09	1.28E-09	1.50E-09			3.61E-09		2.434E-07
	amount at 10-6M	682.2	644.5	626.2			513.5		161.4

Plate #'s		tibolone	methyltestosterone	stanozolol	fluoxymesterone	oxymethalone	danazol	19-norandrostenedione	boldione
AQ1360	Bottom	83.5	80.2	114.4			107.5		78.8
AQ1361	Top	802.2	789.5	761.6			629.3		346.7
AQ1362	LogEC50	-8.567	-8.713	-8.897			-8.346		-6.532
AQ1363	HillSlope	1.138	1.111	0.767			0.671		
	EC50	2.71E-09	1.94E-09	1.27E-09			4.51E-09		2.938E-07
	amount at 10-6M	802.5	788.5	751.8			608.9		286.5
**Plate #'s**		**tibolone**	**methyltestosterone**	**stanozolol**	**fluoxymesterone**	**oxymethalone**	**danazol**	**19-norandrostenedione**	**boldione**
AQ301	Bottom								
	Top								
	LogEC50								
	HillSlope								
	EC50								
	amount at 10-6M								
**Plate #'s**		**tibolone**	**methyltestosterone**	**stanozolol**	**fluoxymesterone**	**oxymethalone**	**danazol**	**19-norandrostenedione**	**boldione**
AQ312	Bottom								
	Top								
	LogEC50								
	HillSlope								
	EC50								
	amount at 10-6M								
**Plate #'s**		**tibolone**	**methyltestosterone**	**stanozolol**	**fluoxymesterone**	**oxymethalone**	**danazol**	**19-norandrostenedione**	**boldione**
AQ352	Bottom								
	Top								
	LogEC50								
	HillSlope								
	EC50								
	amount at 10-6M								
**Plate #'s**		**tibolone**	**methyltestosterone**	**stanozolol**	**fluoxymesterone**	**oxymethalone**	**danazol**	**19-norandrostenedione**	**boldione**
AQ273	Bottom								
	Top								
	LogEC50								
	HillSlope								
	EC50								
	amount at 10-6M								

Plate #'s		tibolone	methyltestosterone	stanozolol	fluoxymesterone	oxymethalone	danazol	19-norandrostenedione	boldione
AQ327	Bottom								
	Top								
	LogEC50								
	HillSlope								
	EC50								
	amount at 10-6M								
Plate #'s		tibolone	methyltestosterone	stanozolol	fluoxymesterone	oxymethalone	danazol	19-norandrostenedione	boldione
AQ290	Bottom								
	Top								
	LogEC50								
	HillSlope								
	EC50								
	amount at 10-6M								
Plate #'s		tibolone	methyltestosterone	stanozolol	fluoxymesterone	oxymethalone	danazol	19-norandrostenedione	boldione
AQ711	Bottom								
	Top								
	LogEC50								
	HillSlope								
	EC50								
	amount at 10-6M								
Plate #'s		tibolone	methyltestosterone	stanozolol	fluoxymesterone	oxymethalone	danazol	19-norandrostenedione	boldione
AQ733	Bottom								
	Top								
	LogEC50								
	HillSlope								
	EC50								
	amount at 10-6M								
Plate #'s		tibolone	methyltestosterone	stanozolol	fluoxymesterone	oxymethalone	danazol	19-norandrostenedione	boldione
AQ735	Bottom								
	Top								
	LogEC50								
	HillSlope								
	EC50								
	amount at 10-6M								

	m26/33a	m26/33b	m27
	**17a-methyl-5a-androstane-3a,17b-diol**	**17a-methyl-5b-androstane-3a,17b-diol**	**1a-methyl-5a-androstane-3a-ol-17-one**
**avg EC50 (M)**	**6.38E-08**		
**avg EC50 (nM)**	**63.78**	**10,000**	**10,000**
**avg log EC50 for those reaching maximum**	**-7.20**		
**est. log EC50 if no max activity at 10-6M**			
**avg log EC50**	**-7.20**	**-15.63**	**-5.97**
**sd log EC50**	**0.13**	**6.57**	**0.56**
**n log EC50**	**3**	**3**	**3**
**min log EC50**	**-7.34**		
**max log EC50**	**-7.10**		
sd EC50 lower range	47.79		
sd EC50 upper range	85.11		
MW	306.49	306.49	304.50
**ng/ml at EC50**	**19.55**	**3,065**	**3,045**
**detectable level in 0.5u/40ul Assay**	**201.38**	**245,192**	**243,600**
**Plate #'s**	**17a-methyl-5a-androstane-3a,17b-diol**	**17a-methyl-5b-androstane-3a,17b-diol**	**1a-methyl-5a-androstane-3a-ol-17-one**
AQ55 Bottom			
AQ56 Top			
LogEC50			
HillSlope			
EC50			
amount at 10-6M			
**Plate #'s**	**17a-methyl-5a-androstane-3a,17b-diol**	**17a-methyl-5b-androstane-3a,17b-diol**	**1a-methyl-5a-androstane-3a-ol-17-one**
AQ286 Bottom			
AQ287 Top			
LogEC50			
HillSlope			
EC50			
amount at 10-6M			

Plate #'s		17a-methyl-5a-androstane-3a,17b-diol	17a-methyl-5b-androstane-3a,17b-diol	1a-methyl-5a-androstane-3a-ol-17-one
AQ520	Bottom			
AQ521	Top			
	LogEC50			
	HillSlope			
	EC50			
	amount at 10-6M			

Plate #'s		17a-methyl-5a-androstane-3a,17b-diol	17a-methyl-5b-androstane-3a,17b-diol	1a-methyl-5a-androstane-3a-ol-17-one
AQ558	Bottom			
AQ559	Top			
	LogEC50			
	HillSlope			
	EC50			
	amount at 10-6M			

Plate #'s		17a-methyl-5a-androstane-3a,17b-diol	17a-methyl-5b-androstane-3a,17b-diol	1a-methyl-5a-androstane-3a-ol-17-one
AQ578	Bottom			
AQ579	Top			
	LogEC50			
	HillSlope			
	EC50			
	amount at 10-6M			

Plate #'s		17a-methyl-5a-androstane-3a,17b-diol	17a-methyl-5b-androstane-3a,17b-diol	1a-methyl-5a-androstane-3a-ol-17-one
AQ595	Bottom			
AQ597	Top			
	LogEC50			
	HillSlope			
	EC50			
	amount at 10-6M			

Plate #'s		17a-methyl-5a-androstane-3a,17b-diol	17a-methyl-5b-androstane-3a,17b-diol	1a-methyl-5a-androstane-3a-ol-17-one
AQ636	Bottom			
	Top			
	LogEC50			
	HillSlope			
	EC50			
	amount at 10-6M			

Plate #'s		17a-methyl-5a-androstane-3a,17b-diol	17a-methyl-5b-androstane-3a,17b-diol	1a-methyl-5a-androstane-3a-ol-17-one
AQ641	Bottom			
	Top			
	LogEC50			
	HillSlope			
	EC50			
	amount at 10-6M			
**Plate #'s**		**17a-methyl-5a-androstane-3a,17b-diol**	**17a-methyl-5b-androstane-3a,17b-diol**	**1a-methyl-5a-androstane-3a-ol-17-one**
AQ662	Bottom			
AQ664	Top			
	LogEC50			
	HillSlope			
	EC50			
	amount at 10-6M			
**Plate #'s**		**17a-methyl-5a-androstane-3a,17b-diol**	**17a-methyl-5b-androstane-3a,17b-diol**	**1a-methyl-5a-androstane-3a-ol-17-one**
AQ694	Bottom			
AQ696	Top			
	LogEC50			
	HillSlope			
	EC50			
	amount at 10-6M			
**Plate #'s**		**17a-methyl-5a-androstane-3a,17b-diol**	**17a-methyl-5b-androstane-3a,17b-diol**	**1a-methyl-5a-androstane-3a-ol-17-one**
AQ706	Bottom			
	Top			
	LogEC50			
	HillSlope			
	EC50			
	amount at 10-6M			
**Plate #'s**		**17a-methyl-5a-androstane-3a,17b-diol**	**17a-methyl-5b-androstane-3a,17b-diol**	**1a-methyl-5a-androstane-3a-ol-17-one**
AQ745	Bottom			
AQ746	Top			
	LogEC50			
	HillSlope			
	EC50			
	amount at 10-6M			

Plate #'s		17a-methyl-5a-androstane-3a,17b-diol	17a-methyl-5b-androstane-3a,17b-diol	1a-methyl-5a-androstane-3a-ol-17-one
AQ295	Bottom			
	Top			
	LogEC50			
	HillSlope			
	EC50			
	amount at 10-6M			
**Plate #'s**		**17a-methyl-5a-androstane-3a,17b-diol**	**17a-methyl-5b-androstane-3a,17b-diol**	**1a-methyl-5a-androstane-3a-ol-17-one**
AQ296	Bottom			
	Top			
	LogEC50			
	HillSlope			
	EC50			
	amount at 10-6M			
**Plate #'s**		**17a-methyl-5a-androstane-3a,17b-diol**	**17a-methyl-5b-androstane-3a,17b-diol**	**1a-methyl-5a-androstane-3a-ol-17-one**
AQ897	Bottom			
	Top			
	LogEC50			
	HillSlope			
	EC50			
	amount at 10-6M			
**Plate #'s**		**17a-methyl-5a-androstane-3a,17b-diol**	**17a-methyl-5b-androstane-3a,17b-diol**	**1a-methyl-5a-androstane-3a-ol-17-one**
AQ1347	Bottom	108.2	105.9	91.2
AQ1348	Top	500.1	104.8	219.8
AQ1349	LogEC50	-7.150	-23.100	-6.399
AQ1350	HillSlope			1.195
	EC50	7.082E-08	7.937E-24	3.99E-07
	amount at 10-6M	484.7	113.2	188.1
**Plate #'s**		**17a-methyl-5a-androstane-3a,17b-diol**	**17a-methyl-5b-androstane-3a,17b-diol**	**1a-methyl-5a-androstane-3a-ol-17-one**
AQ1355	Bottom	85.0	65.8	77.0
AQ1356	Top	462.3	77.8	114.8
AQ1357	LogEC50	-7.337	-10.760	-6.168
AQ1358	HillSlope		20.940	1.055
	EC50	4.607E-08	1.75E-11	6.80E-07
	amount at 10-6M	446.7	80.1	99.9

Plate #'s		17a-methyl-5a-androstane-3a,17b-diol	17a-methyl-5b-androstane-3a,17b-diol	1a-methyl-5a-androstane-3a-ol-17-one
AQ1360	Bottom	79.1	44.3	72.6
AQ1361	Top	827.6	81.2	504.6
AQ1362	LogEC50	-7.099	-13.030	-5.340
AQ1363	HillSlope	0.805	9.041	0.948
	EC50	7.95E-08	9.37E-14	4.57E-06
	amount at 10-6M	723.2	87.1	155.7

Plate #'s		17a-methyl-5a-androstane-3a,17b-diol	17a-methyl-5b-androstane-3a,17b-diol	1a-methyl-5a-androstane-3a-ol-17-one
AQ301	Bottom			
	Top			
	LogEC50			
	HillSlope			
	EC50			
	amount at 10-6M			

Plate #'s		17a-methyl-5a-androstane-3a,17b-diol	17a-methyl-5b-androstane-3a,17b-diol	1a-methyl-5a-androstane-3a-ol-17-one
AQ312	Bottom			
	Top			
	LogEC50			
	HillSlope			
	EC50			
	amount at 10-6M			

Plate #'s		17a-methyl-5a-androstane-3a,17b-diol	17a-methyl-5b-androstane-3a,17b-diol	1a-methyl-5a-androstane-3a-ol-17-one
AQ352	Bottom			
	Top			
	LogEC50			
	HillSlope			
	EC50			
	amount at 10-6M			

Plate #'s		17a-methyl-5a-androstane-3a,17b-diol	17a-methyl-5b-androstane-3a,17b-diol	1a-methyl-5a-androstane-3a-ol-17-one
AQ273	Bottom			
	Top			
	LogEC50			
	HillSlope			
	EC50			
	amount at 10-6M			

Plate #'s		17a-methyl-5a-androstane-3a,17b-diol	17a-methyl-5b-androstane-3a,17b-diol	1a-methyl-5a-androstane-3a-ol-17-one
AQ327	Bottom			
	Top			
	LogEC50			
	HillSlope			
	EC50			
	amount at 10-6M			
**Plate #'s**		**17a-methyl-5a-androstane-3a,17b-diol**	**17a-methyl-5b-androstane-3a,17b-diol**	**1a-methyl-5a-androstane-3a-ol-17-one**
AQ290	Bottom			
	Top			
	LogEC50			
	HillSlope			
	EC50			
	amount at 10-6M			
**Plate #'s**		**17a-methyl-5a-androstane-3a,17b-diol**	**17a-methyl-5b-androstane-3a,17b-diol**	**1a-methyl-5a-androstane-3a-ol-17-one**
AQ711	Bottom			
	Top			
	LogEC50			
	HillSlope			
	EC50			
	amount at 10-6M			
**Plate #'s**		**17a-methyl-5a-androstane-3a,17b-diol**	**17a-methyl-5b-androstane-3a,17b-diol**	**1a-methyl-5a-androstane-3a-ol-17-one**
AQ733	Bottom			
	Top			
	LogEC50			
	HillSlope			
	EC50			
	amount at 10-6M			
**Plate #'s**		**17a-methyl-5a-androstane-3a,17b-diol**	**17a-methyl-5b-androstane-3a,17b-diol**	**1a-methyl-5a-androstane-3a-ol-17-one**
AQ735	Bottom			
	Top			
	LogEC50			
	HillSlope			
	EC50			
	amount at 10-6M			

	m30	m31	m32	m34a	m34b
	19-Norandrosterone	7a-Trenbolone	3-Hydroxytibolone mixture	16b-hydroxystanozolol	3'-OH-stanozolol
**avg EC50 (M)**		**1.67E-08**			**9.08E-09**
**avg EC50 (nM)**	**10,000**	**16.72**	**10,000**	**10,000**	**1.80**
**avg log EC50 for those reaching maximum**		**-7.78**			**-8.04**
**est. log EC50 if no max activity at 10-6M**					
**avg log EC50**	**-6.50**	**-7.78**	**-5.33**	**-7.36**	**-8.74**
**sd log EC50**	**2.83**	**0.42**	**1.13**	**0.44**	**1.17**
**n log EC50**	**3**	**3**	**3**	**3**	**3**
**min log EC50**		**-8.23**			**-10.09**
**max log EC50**		**-7.41**			**-8.04**
sd EC50 lower range		6.34			0.12
sd EC50 upper range		44.10			26.35
MW	276.42	270.40	328.45	344.50	344.50
**ng/ml at EC50**	**2,764**	**4.52**	**3,285**	**3,445**	**0.62**
**detectable level in 0.5u/40ul Assay**	**221,136**	**60.17**	**262,760**	**275,600**	**5.74**
**Plate #'s**	**19-Norandrosterone**	**7a-Trenbolone**	**3-Hydroxytibolone mixture**	**16b-hydroxystanozolol**	**3'-OH-stanozolol**
AQ55 Bottom					
AQ56 Top					
LogEC50					
HillSlope					
EC50					
amount at 10-6M					
**Plate #'s**	**19-Norandrosterone**	**7a-Trenbolone**	**3-Hydroxytibolone mixture**	**16b-hydroxystanozolol**	**3'-OH-stanozolol**
AQ286 Bottom					
AQ287 Top					
LogEC50					
HillSlope					
EC50					
amount at 10-6M					

Plate #'s		19-Norandrosterone	7a-Trenbolone	3-Hydroxytibolone mixture	16b-hydroxystanozolol	3'-OH-stanozolol
AQ520	Bottom					
AQ521	Top					
	LogEC50					
	HillSlope					
	EC50					
	amount at 10-6M					
**Plate #'s**		**19-Norandrosterone**	**7a-Trenbolone**	**3-Hydroxytibolone mixture**	**16b-hydroxystanozolol**	**3'-OH-stanozolol**
AQ558	Bottom					
AQ559	Top					
	LogEC50					
	HillSlope					
	EC50					
	amount at 10-6M					
**Plate #'s**		**19-Norandrosterone**	**7a-Trenbolone**	**3-Hydroxytibolone mixture**	**16b-hydroxystanozolol**	**3'-OH-stanozolol**
AQ578	Bottom					
AQ579	Top					
	LogEC50					
	HillSlope					
	EC50					
	amount at 10-6M					
**Plate #'s**		**19-Norandrosterone**	**7a-Trenbolone**	**3-Hydroxytibolone mixture**	**16b-hydroxystanozolol**	**3'-OH-stanozolol**
AQ595	Bottom					
AQ597	Top					
	LogEC50					
	HillSlope					
	EC50					
	amount at 10-6M					
**Plate #'s**		**19-Norandrosterone**	**7a-Trenbolone**	**3-Hydroxytibolone mixture**	**16b-hydroxystanozolol**	**3'-OH-stanozolol**
AQ636	Bottom					
	Top					
	LogEC50					
	HillSlope					
	EC50					
	amount at 10-6M					

Plate #'s		19-Norandrosterone	7a-Trenbolone	3-Hydroxytibolone mixture	16b-hydroxystanozolol	3'-OH-stanozolol
AQ641	Bottom					
	Top					
	LogEC50					
	HillSlope					
	EC50					
	amount at 10-6M					
**Plate #'s**		**19-Norandrosterone**	**7a-Trenbolone**	**3-Hydroxytibolone mixture**	**16b-hydroxystanozolol**	**3'-OH-stanozolol**
AQ662	Bottom					
AQ664	Top					
	LogEC50					
	HillSlope					
	EC50					
	amount at 10-6M					
**Plate #'s**		**19-Norandrosterone**	**7a-Trenbolone**	**3-Hydroxytibolone mixture**	**16b-hydroxystanozolol**	**3'-OH-stanozolol**
AQ694	Bottom					
AQ696	Top					
	LogEC50					
	HillSlope					
	EC50					
	amount at 10-6M					
**Plate #'s**		**19-Norandrosterone**	**7a-Trenbolone**	**3-Hydroxytibolone mixture**	**16b-hydroxystanozolol**	**3'-OH-stanozolol**
AQ706	Bottom					
	Top					
	LogEC50					
	HillSlope					
	EC50					
	amount at 10-6M					
**Plate #'s**		**19-Norandrosterone**	**7a-Trenbolone**	**3-Hydroxytibolone mixture**	**16b-hydroxystanozolol**	**3'-OH-stanozolol**
AQ745	Bottom					
AQ746	Top					
	LogEC50					
	HillSlope					
	EC50					
	amount at 10-6M					

Plate #'s		19-Norandrosterone	7a-Trenbolone	3-Hydroxytibolone mixture	16b-hydroxystanozolol	3'-OH-stanozolol
AQ295	Bottom					
	Top					
	LogEC50					
	HillSlope					
	EC50					
	amount at 10-6M					
**Plate #'s**		**19-Norandrosterone**	**7a-Trenbolone**	**3-Hydroxytibolone mixture**	**16b-hydroxystanozolol**	**3'-OH-stanozolol**
AQ296	Bottom					
	Top					
	LogEC50					
	HillSlope					
	EC50					
	amount at 10-6M					
**Plate #'s**		**19-Norandrosterone**	**7a-Trenbolone**	**3-Hydroxytibolone mixture**	**16b-hydroxystanozolol**	**3'-OH-stanozolol**
AQ897	Bottom					
	Top					
	LogEC50					
	HillSlope					
	EC50					
	amount at 10-6M					
**Plate #'s**		**19-Norandrosterone**	**7a-Trenbolone**	**3-Hydroxytibolone mixture**	**16b-hydroxystanozolol**	**3'-OH-stanozolol**
AQ1347	Bottom	94.0	104.0	100.8	104.8	123.4
AQ1348	Top	129.6	363.9	188.7	248.1	648.4
AQ1349	LogEC50	-6.496	-8.234	-6.523	-6.850	-10.090
AQ1350	HillSlope	1.594	0.577	0.836		
	EC50	3.19E-07	5.84E-09	3.00E-07	1.41E-07	8.14E-11
	amount at 10-6M	124.7	346.5	166.1	109.2	230.3
**Plate #'s**		**19-Norandrosterone**	**7a-Trenbolone**	**3-Hydroxytibolone mixture**	**16b-hydroxystanozolol**	**3'-OH-stanozolol**
AQ1355	Bottom	72.3	79.3	79.7	70.4	77.3
AQ1356	Top	81.1	438.9	296.9	182.0	490.6
AQ1357	LogEC50	-9.330	-7.691	-5.193	-7.544	-8.102
AQ1358	HillSlope		0.886	0.529	0.745	0.807
	EC50	4.679E-10	2.04E-08	6.41E-06	2.86E-08	7.91E-09
	amount at 10-6M	90.5	427.7	137.8	173.6	482.6

Plate #'s		19-Norandrosterone	7a-Trenbolone	3-Hydroxytibolone mixture	16b-hydroxystanozolol	3'-OH-stanozolol
AQ1360	Bottom	68.1	74.6	72.6	90.5	100.5
AQ1361	Top	523.0	664.8	631.5	152.0	782.2
AQ1362	LogEC50	-3.670	-7.405	-4.285	-7.673	-8.042
AQ1363	HillSlope	0.480	0.890	0.404	3.095	1.299
	EC50	2.14E-04	3.94E-08	5.18E-05	2.13E-08	9.07E-09
	amount at 10-6M	104.4	640.9	165.5	125.5	776.0

Plate #'s		19-Norandrosterone	7a-Trenbolone	3-Hydroxytibolone mixture	16b-hydroxystanozolol	3'-OH-stanozolol
AQ301	Bottom					
	Top					
	LogEC50					
	HillSlope					
	EC50					
	amount at 10-6M					

Plate #'s		19-Norandrosterone	7a-Trenbolone	3-Hydroxytibolone mixture	16b-hydroxystanozolol	3'-OH-stanozolol
AQ312	Bottom					
	Top					
	LogEC50					
	HillSlope					
	EC50					
	amount at 10-6M					

Plate #'s		19-Norandrosterone	7a-Trenbolone	3-Hydroxytibolone mixture	16b-hydroxystanozolol	3'-OH-stanozolol
AQ352	Bottom					
	Top					
	LogEC50					
	HillSlope					
	EC50					
	amount at 10-6M					

Plate #'s		19-Norandrosterone	7a-Trenbolone	3-Hydroxytibolone mixture	16b-hydroxystanozolol	3'-OH-stanozolol
AQ273	Bottom					
	Top					
	LogEC50					
	HillSlope					
	EC50					
	amount at 10-6M					

Plate #'s		19-Norandrosterone	7a-Trenbolone	3-Hydroxytibolone mixture	16b-hydroxystanozolol	3'-OH-stanozolol
AQ327	Bottom					
	Top					
	LogEC50					
	HillSlope					
	EC50					
	amount at 10-6M					
**Plate #'s**		**19-Norandrosterone**	**7a-Trenbolone**	**3-Hydroxytibolone mixture**	**16b-hydroxystanozolol**	**3'-OH-stanozolol**
AQ290	Bottom					
	Top					
	LogEC50					
	HillSlope					
	EC50					
	amount at 10-6M					
**Plate #'s**		**19-Norandrosterone**	**7a-Trenbolone**	**3-Hydroxytibolone mixture**	**16b-hydroxystanozolol**	**3'-OH-stanozolol**
AQ711	Bottom					
	Top					
	LogEC50					
	HillSlope					
	EC50					
	amount at 10-6M					
**Plate #'s**		**19-Norandrosterone**	**7a-Trenbolone**	**3-Hydroxytibolone mixture**	**16b-hydroxystanozolol**	**3'-OH-stanozolol**
AQ733	Bottom					
	Top					
	LogEC50					
	HillSlope					
	EC50					
	amount at 10-6M					
**Plate #'s**		**19-Norandrosterone**	**7a-Trenbolone**	**3-Hydroxytibolone mixture**	**16b-hydroxystanozolol**	**3'-OH-stanozolol**
AQ735	Bottom					
	Top					
	LogEC50					
	HillSlope					
	EC50					
	amount at 10-6M					

	m34c	m35	m37	m39
	4b-hydroxystanozolol	uoro-17a-methyl-androst-4-ene-a,6b,11b,17b-t	17a-Ethynyl-testosterone	5b-androst-1en-17b-ol-3-one
**avg EC50 (M)**			**8.02E-08**	
**avg EC50 (nM)**	**10,000**	**10,000**	**80.23**	**10,000**
**avg log EC50 for those reaching maximum**			**-7.10**	
**est. log EC50 if no max activity at 10-6M**				
**avg log EC50**	**-4.69**	**-5.63**	**-7.10**	**-5.82**
**sd log EC50**	**13.54**	**1.01**	**0.11**	**1.30**
**n log EC50**	**3**	**3**	**3**	**3**
**min log EC50**			**-7.20**	
**max log EC50**			**-6.98**	
sd EC50 lower range			62.15	
sd EC50 upper range			103.57	
MW	344.50	354.50	312.45	288.4
**ng/ml at EC50**	**3,445**	**3,545**	**25.07**	**2,884**
**detectable level in 0.5u/40ul Assay**	**275,600**	**283,600**	**587.88**	**230,720**
**Plate #'s**	4b-hydroxystanozolol	9a-fluoro-17a-methyl-androst-4-ene-a,6b,11b,	17a-Ethynyl-testosterone	5b-androst-1en-17b-ol-3-one
AQ55 Bottom				
AQ56 Top				
LogEC50				
HillSlope				
EC50				
amount at 10-6M				
**Plate #'s**	4b-hydroxystanozolol	9a-fluoro-17a-methyl-androst-4-ene-a,6b,11b,	17a-Ethynyl-testosterone	5b-androst-1en-17b-ol-3-one
AQ286 Bottom				
AQ287 Top				
LogEC50				
HillSlope				
EC50				
amount at 10-6M				

**Plate #'s**		**4b-hydroxystanozolol**	**9a-fluoro-17a-methyl-androst-4-ene-a,6b,11b,**	**17a-Ethynyl-testosterone**	**5b-androst-1en-17b-ol-3-one**
AQ520	Bottom				
AQ521	Top				
	LogEC50				
	HillSlope				
	EC50				
	amount at 10-6M				
**Plate #'s**		**4b-hydroxystanozolol**	**9a-fluoro-17a-methyl-androst-4-ene-a,6b,11b,**	**17a-Ethynyl-testosterone**	**5b-androst-1en-17b-ol-3-one**
AQ558	Bottom				
AQ559	Top				
	LogEC50				
	HillSlope				
	EC50				
	amount at 10-6M				
**Plate #'s**		**4b-hydroxystanozolol**	**9a-fluoro-17a-methyl-androst-4-ene-a,6b,11b,**	**17a-Ethynyl-testosterone**	**5b-androst-1en-17b-ol-3-one**
AQ578	Bottom				
AQ579	Top				
	LogEC50				
	HillSlope				
	EC50				
	amount at 10-6M				
**Plate #'s**		**4b-hydroxystanozolol**	**9a-fluoro-17a-methyl-androst-4-ene-a,6b,11b,**	**17a-Ethynyl-testosterone**	**5b-androst-1en-17b-ol-3-one**
AQ595	Bottom				
AQ597	Top				
	LogEC50				
	HillSlope				
	EC50				
	amount at 10-6M				
**Plate #'s**		**4b-hydroxystanozolol**	**9a-fluoro-17a-methyl-androst-4-ene-a,6b,11b,**	**17a-Ethynyl-testosterone**	**5b-androst-1en-17b-ol-3-one**
AQ636	Bottom				
	Top				
	LogEC50				
	HillSlope				
	EC50				
	amount at 10-6M				

Plate #'s		4b-hydroxystanozolol	9a-fluoro-17a-methyl-androst-4-ene-a,6b,11b,	17a-Ethynyl-testosterone	5b-androst-1en-17b-ol-3-one
AQ641	Bottom				
	Top				
	LogEC50				
	HillSlope				
	EC50				
	amount at 10-6M				

Plate #'s		4b-hydroxystanozolol	9a-fluoro-17a-methyl-androst-4-ene-a,6b,11b,	17a-Ethynyl-testosterone	5b-androst-1en-17b-ol-3-one
AQ662	Bottom				
AQ664	Top				
	LogEC50				
	HillSlope				
	EC50				
	amount at 10-6M				

Plate #'s		4b-hydroxystanozolol	9a-fluoro-17a-methyl-androst-4-ene-a,6b,11b,	17a-Ethynyl-testosterone	5b-androst-1en-17b-ol-3-one
AQ694	Bottom				
AQ696	Top				
	LogEC50				
	HillSlope				
	EC50				
	amount at 10-6M				

Plate #'s		4b-hydroxystanozolol	9a-fluoro-17a-methyl-androst-4-ene-a,6b,11b,	17a-Ethynyl-testosterone	5b-androst-1en-17b-ol-3-one
AQ706	Bottom				
	Top				
	LogEC50				
	HillSlope				
	EC50				
	amount at 10-6M				

Plate #'s		4b-hydroxystanozolol	9a-fluoro-17a-methyl-androst-4-ene-a,6b,11b,	17a-Ethynyl-testosterone	5b-androst-1en-17b-ol-3-one
AQ745	Bottom				
AQ746	Top				
	LogEC50				
	HillSlope				
	EC50				
	amount at 10-6M				

Plate #'s		4b-hydroxystanozolol	9a-fluoro-17a-methyl-androst-4-ene-a,6b,11b,	17a-Ethynyl-testosterone	5b-androst-1en-17b-ol-3-one
AQ295	Bottom				
	Top				
	LogEC50				
	HillSlope				
	EC50				
	amount at 10-6M				
**Plate #'s**		**4b-hydroxystanozolol**	**9a-fluoro-17a-methyl-androst-4-ene-a,6b,11b,**	**17a-Ethynyl-testosterone**	**5b-androst-1en-17b-ol-3-one**
AQ296	Bottom				
	Top				
	LogEC50				
	HillSlope				
	EC50				
	amount at 10-6M				
**Plate #'s**		**4b-hydroxystanozolol**	**9a-fluoro-17a-methyl-androst-4-ene-a,6b,11b,**	**17a-Ethynyl-testosterone**	**5b-androst-1en-17b-ol-3-one**
AQ897	Bottom				
	Top				
	LogEC50				
	HillSlope				
	EC50				
	amount at 10-6M				
**Plate #'s**		**4b-hydroxystanozolol**	**9a-fluoro-17a-methyl-androst-4-ene-a,6b,11b,**	**17a-Ethynyl-testosterone**	**5b-androst-1en-17b-ol-3-one**
AQ1347	Bottom	127.7	86.5	108.3	110.1
AQ1348	Top	102.3	948.7	280.8	213.5
AQ1349	LogEC50	-12.590	-4.537	-7.116	-6.739
AQ1350	HillSlope			1.201	0.000
	EC50	2.57E-13	2.903E-05	7.66E-08	1.82E-07
	amount at 10-6M	130.0	116.3	276.1	199.5
**Plate #'s**		**4b-hydroxystanozolol**	**9a-fluoro-17a-methyl-androst-4-ene-a,6b,11b,**	**17a-Ethynyl-testosterone**	**5b-androst-1en-17b-ol-3-one**
AQ1355	Bottom	70.7	74.2	69.0	72.9
AQ1356	Top	4190000.0	137.0	300.1	217.5
AQ1357	LogEC50	10.950	-5.811	-7.195	-6.388
AQ1358	HillSlope	0.308		0.759	0.780
	EC50	8.83E+10	1.546E-06	6.38E-08	4.10E-07
	amount at 10-6M	99.6	98.3	276.6	168.3

**Plate #'s**		**4b-hydroxystanozolol**	**9a-fluoro-17a-methyl-androst-4-ene-a,6b,11b,**	**17a-Ethynyl-testosterone**	**5b-androst-1en-17b-ol-3-one**
AQ1360	Bottom	79.7	77.3	80.4	80.8
AQ1361	Top	100.3	103.5	393.6	837.7
AQ1362	LogEC50	-12.420	-6.531	-6.976	-4.332
AQ1363	HillSlope	-0.762	2.354		0.574
	EC50	3.82E-13	2.95E-07	1.058E-07	4.66E-05
	amount at 10-6M	102.0	102.1	376.4	157.1
**Plate #'s**		**4b-hydroxystanozolol**	**9a-fluoro-17a-methyl-androst-4-ene-a,6b,11b,**	**17a-Ethynyl-testosterone**	**5b-androst-1en-17b-ol-3-one**
AQ301	Bottom				
	Top				
	LogEC50				
	HillSlope				
	EC50				
	amount at 10-6M				
**Plate #'s**		**4b-hydroxystanozolol**	**9a-fluoro-17a-methyl-androst-4-ene-a,6b,11b,**	**17a-Ethynyl-testosterone**	**5b-androst-1en-17b-ol-3-one**
AQ312	Bottom				
	Top				
	LogEC50				
	HillSlope				
	EC50				
	amount at 10-6M				
**Plate #'s**		**4b-hydroxystanozolol**	**9a-fluoro-17a-methyl-androst-4-ene-a,6b,11b,**	**17a-Ethynyl-testosterone**	**5b-androst-1en-17b-ol-3-one**
AQ352	Bottom				
	Top				
	LogEC50				
	HillSlope				
	EC50				
	amount at 10-6M				
**Plate #'s**		**4b-hydroxystanozolol**	**9a-fluoro-17a-methyl-androst-4-ene-a,6b,11b,**	**17a-Ethynyl-testosterone**	**5b-androst-1en-17b-ol-3-one**
AQ273	Bottom				
	Top				
	LogEC50				
	HillSlope				
	EC50				
	amount at 10-6M				

Plate #'s		4b-hydroxystanozolol	9a-fluoro-17a-methyl-androst-4-ene-a,6b,11b,	17a-Ethynyl-testosterone	5b-androst-1en-17b-ol-3-one
AQ327	Bottom				
	Top				
	LogEC50				
	HillSlope				
	EC50				
	amount at 10-6M				
**Plate #'s**		**4b-hydroxystanozolol**	**9a-fluoro-17a-methyl-androst-4-ene-a,6b,11b,**	**17a-Ethynyl-testosterone**	**5b-androst-1en-17b-ol-3-one**
AQ290	Bottom				
	Top				
	LogEC50				
	HillSlope				
	EC50				
	amount at 10-6M				
**Plate #'s**		**4b-hydroxystanozolol**	**9a-fluoro-17a-methyl-androst-4-ene-a,6b,11b,**	**17a-Ethynyl-testosterone**	**5b-androst-1en-17b-ol-3-one**
AQ711	Bottom				
	Top				
	LogEC50				
	HillSlope				
	EC50				
	amount at 10-6M				
**Plate #'s**		**4b-hydroxystanozolol**	**9a-fluoro-17a-methyl-androst-4-ene-a,6b,11b,**	**17a-Ethynyl-testosterone**	**5b-androst-1en-17b-ol-3-one**
AQ733	Bottom				
	Top				
	LogEC50				
	HillSlope				
	EC50				
	amount at 10-6M				
**Plate #'s**		**4b-hydroxystanozolol**	**9a-fluoro-17a-methyl-androst-4-ene-a,6b,11b,**	**17a-Ethynyl-testosterone**	**5b-androst-1en-17b-ol-3-one**
AQ735	Bottom				
	Top				
	LogEC50				
	HillSlope				
	EC50				
	amount at 10-6M				
