# Supplementary material for: Advantages and Limitations of Androgen Receptor-Based Methods for Detecting Anabolic Androgenic Steroid Abuse as Performance Enhancing Drugs
Source: PLoS One. 2016 Mar 21;11(3):e0151860. doi: 10.1371/journal.pone.0151860 (PMC4801337; doi:10.1371/journal.pone.0151860)
Supplement: S3 Source Data — The difference between the top and bottom of the dose response curves (see data in S2 Source Data) for each compound was compared against that of testosterone measured in parallel. The efficacy relative to testosterone was calculated and shown in this supporting figure. For ligands that do not substantially activate the AR BioAssay, curve fitting can result in nonsensical tops of the curve. In those instances, the AR BioAssay activity at 1 μM compound is shown relative to that of the maximal activation by testosterone. (PDF) [file pone.0151860.s006.pdf]

Legend
T-like' efficacy (max reached at 10-6M)
low efficacy (max reached at 10-6M)
unknown efficacy (max not reached at 10-6M)
**no to minimal activity, levels are >amount indicated**

	A1	A2	A3
	**Testosterone**	**DHT**	**Androstanediol**
	99.96%	106.62%	64.92%
**avg % of Teststerone at max**	99.96%	106.62%	64.92%
**sd % of Teststerone at max**	4.20%	4.28%	9.59%
**n % of Teststerone at max**	53	6	7
**min % of Teststerone at max**	92.63%	101.36%	48.54%
**max % of Teststerone at max**	109.76%	113.00%	79.88%
**avg % of T amount at 10-6M**	100.35%	106.56%	62.25%
**sd % of T amount at 10-6M**	7.09%	8.88%	10.37%
**n % of T amount at 10-6M**	52	6	7
**min % of T amount at 10-6M**	85.56%	94.16%	43.86%
**max % of T amount at 10-6M**	118.46%	115.98%	77.95%

**Plate #'s**		**Testosterone**	Testosterone	Testosterone	Testosterone	**DHT**	**Androstanediol**	Androstanediol
AQ55	Bottom							
AQ56	Top	100.00%						
	LogEC50							
	HillSlope							
	EC50							
	amount at 10-6M	94.71%						

**Plate #'s**		**Testosterone**	Testosterone	Testosterone	Testosterone	**DHT**	**Androstanediol**	Androstanediol
AQ286	Bottom							
AQ287	Top	100.00%				105.81%		
	LogEC50							
	HillSlope							
	EC50							
	amount at 10-6M	106.65%				98.66%		

**Plate #'s**		**Testosterone**	Testosterone	Testosterone	Testosterone	**DHT**	**Androstanediol**	Androstanediol
AQ520	Bottom							
AQ521	Top	99.72%	95.72%	104.56%		109.39%		
	LogEC50							
	HillSlope							
	EC50							
	amount at 10-6M	94.14%	103.75%			114.42%		
**Plate #'s**		**Testosterone**	Testosterone	Testosterone	Testosterone	**DHT**	**Androstanediol**	Androstanediol
AQ558	Bottom							
AQ559	Top	98.76%	102.37%	98.86%				
	LogEC50							
	HillSlope							
	EC50							
	amount at 10-6M	102.36%	101.40%	85.56%				
**Plate #'s**		**Testosterone**	Testosterone	Testosterone	Testosterone	**DHT**	**Androstanediol**	Androstanediol
AQ578	Bottom							
AQ579	Top	102.12%	93.63%	104.25%		102.83%	64.31%	65.29%
	LogEC50							
	HillSlope							
	EC50							
	amount at 10-6M	103.33%	86.51%	100.91%		94.16%	63.46%	58.93%
**Plate #'s**		**Testosterone**	Testosterone	Testosterone	Testosterone	**DHT**	**Androstanediol**	Androstanediol
AQ595	Bottom							
AQ597	Top	94.87%	100.15%	96.72%	108.25%			
	LogEC50							
	HillSlope							
	EC50							
	amount at 10-6M	92.64%	94.96%	96.15%	108.44%			
**Plate #'s**		**Testosterone**	Testosterone	Testosterone	Testosterone	**DHT**	**Androstanediol**	Androstanediol
AQ636	Bottom							
	Top	99.11%	100.89%					
	LogEC50							
	HillSlope							
	EC50							
	amount at 10-6M	100.53%						

Plate #'s		Testosterone	Testosterone	Testosterone	Testosterone	DHT	Androstanediol	Androstanediol
AQ641	Bottom							
	Top	100.00%						
	LogEC50							
	HillSlope							
	EC50							
	amount at 10-6M	94.07%						
**Plate #'s**		**Testosterone**	Testosterone	Testosterone	Testosterone	**DHT**	**Androstanediol**	Androstanediol
AQ662	Bottom							
AQ664	Top	94.63%	101.13%	94.47%	109.76%		48.54%	
	LogEC50							
	HillSlope							
	EC50							
	amount at 10-6M	91.54%	100.88%	90.82%	109.86%		43.86%	
**Plate #'s**		**Testosterone**	Testosterone	Testosterone	Testosterone	**DHT**	**Androstanediol**	Androstanediol
AQ694	Bottom							
AQ696	Top	93.58%	104.44%	94.26%	107.72%			
	LogEC50							
	HillSlope							
	EC50							
	amount at 10-6M	98.91%	106.77%	98.49%	109.21%			
**Plate #'s**		**Testosterone**	Testosterone	Testosterone	Testosterone	**DHT**	**Androstanediol**	Androstanediol
AQ706	Bottom							
	Top	100.00%					70.70%	
	LogEC50							
	HillSlope							
	EC50							
	amount at 10-6M	98.60%					67.58%	
**Plate #'s**		**Testosterone**	Testosterone	Testosterone	Testosterone	**DHT**	**Androstanediol**	Androstanediol
AQ745	Bottom							
AQ746	Top	97.41%	102.59%					
	LogEC50							
	HillSlope							
	EC50							
	amount at 10-6M	97.04%	94.27%					

Plate #'s		Testosterone	Testosterone	Testosterone	Testosterone	DHT	Androstanediol	Androstanediol
AQ295	Bottom							
	Top	103.71%	96.29%					
	LogEC50							
	HillSlope							
	EC50							
	amount at 10-6M	105.70%	91.53%					

Plate #'s		Testosterone	Testosterone	Testosterone	Testosterone	DHT	Androstanediol	Androstanediol
AQ296	Bottom							
	Top	95.97%	104.03%					
	LogEC50							
	HillSlope							
	EC50							
	amount at 10-6M	97.80%	114.87%	104.29%				

Plate #'s		Testosterone	Testosterone	Testosterone	Testosterone	DHT	Androstanediol	Androstanediol
AQ897	Bottom							
	Top	100.00%						
	LogEC50							
	HillSlope							
	EC50							
	amount at 10-6M	112.17%						

Plate #'s		Testosterone	Testosterone	Testosterone	Testosterone	DHT	Androstanediol	Androstanediol
AQ1347	Bottom							
AQ1348	Top	100.92%	93.97%	105.11%				
AQ1349	LogEC50							
AQ1350	HillSlope							
	EC50							
	amount at 10-6M	108.52%	101.56%	118.46%				

Plate #'s		Testosterone	Testosterone	Testosterone	Testosterone	DHT	Androstanediol	Androstanediol
AQ1355	Bottom							
AQ1356	Top	93.69%	98.18%	103.42%	102.55%			
AQ1357	LogEC50							
AQ1358	HillSlope							
	EC50							
	amount at 10-6M	99.10%	94.53%	107.08%	103.42%			

**Plate #'s**		**Testosterone**	Testosterone	Testosterone	Testosterone	**DHT**	**Androstanediol**	Androstanediol
AQ1360	Bottom							
AQ1361	Top	92.63%	97.84%	109.53%				
AQ1362	LogEC50							
AQ1363	HillSlope							
	EC50							
	amount at 10-6M	90.71%	96.11%	112.00%				
**Plate #'s**		**Testosterone**	Testosterone	Testosterone	Testosterone	**DHT**	**Androstanediol**	Androstanediol
AQ301	Bottom							
	Top	100.00%				107.34%	79.88%	
	LogEC50							
	HillSlope							
	EC50							
	amount at 10-6M	99.07%				115.98%	77.95%	
**Plate #'s**		**Testosterone**	Testosterone	Testosterone	Testosterone	**DHT**	**Androstanediol**	Androstanediol
AQ312	Bottom							
	Top	100.00%						
	LogEC50							
	HillSlope							
	EC50							
	amount at 10-6M	99.34%						
**Plate #'s**		**Testosterone**	Testosterone	Testosterone	Testosterone	**DHT**	**Androstanediol**	Androstanediol
AQ352	Bottom							
	Top	100.00%						
	LogEC50							
	HillSlope							
	EC50							
	amount at 10-6M	98.38%						
**Plate #'s**		**Testosterone**	Testosterone	Testosterone	Testosterone	**DHT**	**Androstanediol**	Androstanediol
AQ273	Bottom							
	Top	100.00%				113.00%	65.81%	
	LogEC50							
	HillSlope							
	EC50							
	amount at 10-6M	94.58%				111.43%	65.15%	

**Plate #'s**		**Testosterone**	Testosterone	Testosterone	Testosterone	**DHT**	**Androstanediol**	Androstanediol
AQ327	Bottom							
	Top	100.00%						
	LogEC50							
	HillSlope							
	EC50							
	amount at 10-6M	102.68%						
**Plate #'s**		**Testosterone**	Testosterone	Testosterone	Testosterone	**DHT**	**Androstanediol**	Androstanediol
AQ290	Bottom							
	Top	100.00%						
	LogEC50							
	HillSlope							
	EC50							
	amount at 10-6M	95.50%						
**Plate #'s**		**Testosterone**	Testosterone	Testosterone	Testosterone	**DHT**	**Androstanediol**	Androstanediol
AQ711	Bottom							
	Top	95.40%	104.60%			101.36%	59.89%	
	LogEC50							
	HillSlope							
	EC50							
	amount at 10-6M	98.65%	109.69%			104.71%	58.85%	
**Plate #'s**		**Testosterone**	Testosterone	Testosterone	Testosterone	**DHT**	**Androstanediol**	Androstanediol
AQ733	Bottom							
	Top	100.00%						
	LogEC50							
	HillSlope							
	EC50							
	amount at 10-6M	99.79%						
**Plate #'s**		**Testosterone**	Testosterone	Testosterone	Testosterone	**DHT**	**Androstanediol**	Androstanediol
AQ735	Bottom							
	Top							
	LogEC50							
	HillSlope							
	EC50							
	amount at 10-6M							

	A4		A5	A6	A7	A8	A9
	**Androstendione**		**Androstenediol-4**	**Androstendiol-5**	**epitestosterone**	**DHEA**	**Androsterone**
	39.66%		54.18%	15.49%	9.24%	3.42%	6.90%
**avg % of Teststerone at max**	39.66%		54.18%				
**sd % of Teststerone at max**	19.01%		13.05%				
**n % of Teststerone at max**	12		9				
**min % of Teststerone at max**	20.19%		43.24%				
**max % of Teststerone at max**	81.92%		85.72%				
**avg % of T amount at 10-6M**	34.43%		51.31%	15.49%	9.24%	3.42%	6.90%
**sd % of T amount at 10-6M**	14.58%		13.62%	6.62%	4.92%	4.87%	3.20%
**n % of T amount at 10-6M**	12		9	6	4	7	6
**min % of T amount at 10-6M**	10.62%		33.15%	9.21%	5.74%	-1.93%	1.69%
**max % of T amount at 10-6M**	61.69%		81.29%	23.29%	16.47%	13.57%	10.44%

**Plate #'s**		**Androstendione**	Androstendione	**Androstenediol-4**	**Androstendiol-5**	**epitestosterone**	**DHEA**	**Androsterone**
AQ55	Bottom							
AQ56	Top					9.27%	11.61%	6.36%
	LogEC50							
	HillSlope							
	EC50							
	amount at 10-6M					8.09%	3.78%	6.18%

**Plate #'s**		**Androstendione**	Androstendione	**Androstenediol-4**	**Androstendiol-5**	**epitestosterone**	**DHEA**	**Androsterone**
AQ286	Bottom							
AQ287	Top					3508031.14%	-4.71%	
	LogEC50							
	HillSlope							
	EC50							
	amount at 10-6M					16.47%	13.57%	

**Plate #'s**		**Androstendione**	Androstendione	**Androstenediol-4**	**Androstendiol-5**	**epitestosterone**	**DHEA**	**Androsterone**
AQ520	Bottom							
AQ521	Top					6.68%	4.98%	0.37%
	LogEC50							
	HillSlope							
	EC50							
	amount at 10-6M					5.74%	-1.93%	1.69%
**Plate #'s**		**Androstendione**	Androstendione	**Androstenediol-4**	**Androstendiol-5**	**epitestosterone**	**DHEA**	**Androsterone**
AQ558	Bottom							
AQ559	Top						4605.63%	
	LogEC50							
	HillSlope							
	EC50							
	amount at 10-6M						2.97%	
**Plate #'s**		**Androstendione**	Androstendione	**Androstenediol-4**	**Androstendiol-5**	**epitestosterone**	**DHEA**	**Androsterone**
AQ578	Bottom							
AQ579	Top			85.72%	10.42%			
	LogEC50							
	HillSlope							
	EC50							
	amount at 10-6M			81.29%	9.62%			
**Plate #'s**		**Androstendione**	Androstendione	**Androstenediol-4**	**Androstendiol-5**	**epitestosterone**	**DHEA**	**Androsterone**
AQ595	Bottom							
AQ597	Top	35.72%						
	LogEC50							
	HillSlope							
	EC50							
	amount at 10-6M	34.81%						
**Plate #'s**		**Androstendione**	Androstendione	**Androstenediol-4**	**Androstendiol-5**	**epitestosterone**	**DHEA**	**Androsterone**
AQ636	Bottom							
	Top	34.70%		43.24%				
	LogEC50							
	HillSlope							
	EC50							
	amount at 10-6M	31.82%		42.11%				

Plate #'s		Androstendione	Androstendione	Androstenediol-4	Androstendiol-5	epitestosterone	DHEA	Androsterone
AQ641	Bottom							
	Top	22.97%	27.63%	43.56%				
	LogEC50							
	HillSlope							
	EC50							
	amount at 10-6M	22.25%	25.61%	42.61%				
**Plate #'s**		**Androstendione**	Androstendione	**Androstenediol-4**	**Androstendiol-5**	**epitestosterone**	**DHEA**	**Androsterone**
AQ662	Bottom							
AQ664	Top	20.19%	37.45%	48.36%	10.89%			
	LogEC50							
	HillSlope							
	EC50							
	amount at 10-6M	10.62%	32.31%	46.22%	10.10%			
**Plate #'s**		**Androstendione**	Androstendione	**Androstenediol-4**	**Androstendiol-5**	**epitestosterone**	**DHEA**	**Androsterone**
AQ694	Bottom							
AQ696	Top	21.03%		48.20%				
	LogEC50							
	HillSlope							
	EC50							
	amount at 10-6M	20.19%		50.04%				
**Plate #'s**		**Androstendione**	Androstendione	**Androstenediol-4**	**Androstendiol-5**	**epitestosterone**	**DHEA**	**Androsterone**
AQ706	Bottom							
	Top							
	LogEC50							
	HillSlope							
	EC50							
	amount at 10-6M							8.27%
**Plate #'s**		**Androstendione**	Androstendione	**Androstenediol-4**	**Androstendiol-5**	**epitestosterone**	**DHEA**	**Androsterone**
AQ745	Bottom							
AQ746	Top			48.69%	5.83%			
	LogEC50							
	HillSlope							
	EC50							
	amount at 10-6M			33.15%	9.21%			

Plate #'s		**Androstendione**	Androstendione	**Androstenediol-4**	**Androstendiol-5**	**epitestosterone**	**DHEA**	**Androsterone**
AQ295	Bottom							
	Top	81.92%						
	LogEC50							
	HillSlope							
	EC50							
	amount at 10-6M	56.18%						
**Plate #'s**		**Androstendione**	Androstendione	**Androstenediol-4**	**Androstendiol-5**	**epitestosterone**	**DHEA**	**Androsterone**
AQ296	Bottom							
	Top	70.21%						
	LogEC50							
	HillSlope							
	EC50							
	amount at 10-6M	61.69%						
**Plate #'s**		**Androstendione**	Androstendione	**Androstenediol-4**	**Androstendiol-5**	**epitestosterone**	**DHEA**	**Androsterone**
AQ897	Bottom							
	Top					7.78%		
	LogEC50							
	HillSlope							
	EC50							
	amount at 10-6M					6.64%		
**Plate #'s**		**Androstendione**	Androstendione	**Androstenediol-4**	**Androstendiol-5**	**epitestosterone**	**DHEA**	**Androsterone**
AQ1347	Bottom							
AQ1348	Top							
AQ1349	LogEC50							
AQ1350	HillSlope							
	EC50							
	amount at 10-6M							
**Plate #'s**		**Androstendione**	Androstendione	**Androstenediol-4**	**Androstendiol-5**	**epitestosterone**	**DHEA**	**Androsterone**
AQ1355	Bottom							
AQ1356	Top							
AQ1357	LogEC50							
AQ1358	HillSlope							
	EC50							
	amount at 10-6M							

Plate #'s		Androstendione	Androstendione	Androstenediol-4	Androstendiol-5	epitestosterone	DHEA	Androsterone
AQ1360	Bottom							
AQ1361	Top							
AQ1362	LogEC50							
AQ1363	HillSlope							
	EC50							
	amount at 10-6M							
**Plate #'s**		**Androstendione**	Androstendione	**Androstenediol-4**	**Androstendiol-5**	**epitestosterone**	**DHEA**	**Androsterone**
AQ301	Bottom							
	Top	46.22%						17.11%
	LogEC50							
	HillSlope							
	EC50							
	amount at 10-6M	43.71%					3.06%	10.44%
**Plate #'s**		**Androstendione**	Androstendione	**Androstenediol-4**	**Androstendiol-5**	**epitestosterone**	**DHEA**	**Androsterone**
AQ312	Bottom							
	Top							
	LogEC50							
	HillSlope							
	EC50							
	amount at 10-6M							
**Plate #'s**		**Androstendione**	Androstendione	**Androstenediol-4**	**Androstendiol-5**	**epitestosterone**	**DHEA**	**Androsterone**
AQ352	Bottom							
	Top			53.24%	22.92%			
	LogEC50							
	HillSlope							
	EC50							
	amount at 10-6M			52.43%	22.28%			
**Plate #'s**		**Androstendione**	Androstendione	**Androstenediol-4**	**Androstendiol-5**	**epitestosterone**	**DHEA**	**Androsterone**
AQ273	Bottom							
	Top	41.78%						16.31%
	LogEC50							
	HillSlope							
	EC50							
	amount at 10-6M	39.80%					1.76%	9.48%

Plate #'s		**Androstendione**	Androstendione	**Androstenediol-4**	**Androstendiol-5**	**epitestosterone**	**DHEA**	**Androsterone**
AQ327	Bottom							
	Top							
	LogEC50							
	HillSlope							
	EC50							
	amount at 10-6M							
**Plate #'s**		**Androstendione**	Androstendione	**Androstenediol-4**	**Androstendiol-5**	**epitestosterone**	**DHEA**	**Androsterone**
AQ290	Bottom							
	Top			59.19%	18.60%			
	LogEC50							
	HillSlope							
	EC50							
	amount at 10-6M			58.36%	18.47%			
**Plate #'s**		**Androstendione**	Androstendione	**Androstenediol-4**	**Androstendiol-5**	**epitestosterone**	**DHEA**	**Androsterone**
AQ711	Bottom							
	Top	36.11%						5.48%
	LogEC50							
	HillSlope							
	EC50							
	amount at 10-6M	34.13%					0.73%	5.34%
**Plate #'s**		**Androstendione**	Androstendione	**Androstenediol-4**	**Androstendiol-5**	**epitestosterone**	**DHEA**	**Androsterone**
AQ733	Bottom							
	Top							
	LogEC50							
	HillSlope							
	EC50							
	amount at 10-6M							
**Plate #'s**		**Androstendione**	Androstendione	**Androstenediol-4**	**Androstendiol-5**	**epitestosterone**	**DHEA**	**Androsterone**
AQ735	Bottom							
	Top			57.40%	23.46%			
	LogEC50							
	HillSlope							
	EC50							
	amount at 10-6M			55.55%	23.29%			

	P10	P11	P12	P13	P14
	5a-pregnan-17a-ol-3,20-dione	17a-Hydroxyprogesterone	17OH-pregnenolone	pregnenolone	progesterone
	2.92%	4.37%	3.54%	4.34%	13.42%
**avg % of Teststerone at max**					
**sd % of Teststerone at max**					
**n % of Teststerone at max**					
**min % of Teststerone at max**					
**max % of Teststerone at max**					
**avg % of T amount at 10-6M**	2.92%	4.37%	3.54%	4.34%	13.42%
**sd % of T amount at 10-6M**	4.57%	3.80%	2.21%	2.93%	2.29%
**n % of T amount at 10-6M**	6	7	4	4	4
**min % of T amount at 10-6M**	-0.09%	-0.41%	1.67%	-0.02%	11.22%
**max % of T amount at 10-6M**	12.01%	10.77%	6.58%	6.10%	16.61%

**Plate #'s**		**5a-pregnan-17a-ol-3,20-dione**	**17a-Hydroxyprogesterone**	**17OH-pregnenolone**	**pregnenolone**	**progesterone**
AQ55	Bottom					
AQ56	Top					
	LogEC50					
	HillSlope					
	EC50					
	amount at 10-6M					

**Plate #'s**		**5a-pregnan-17a-ol-3,20-dione**	**17a-Hydroxyprogesterone**	**17OH-pregnenolone**	**pregnenolone**	**progesterone**
AQ286	Bottom					
AQ287	Top					
	LogEC50					
	HillSlope					
	EC50					
	amount at 10-6M					

Plate #'s		5a-pregnan-17a-ol-3,20-dione	17a-Hydroxyprogesterone	17OH-pregnenolone	pregnenolone	progesterone
AQ520	Bottom					
AQ521	Top					
	LogEC50					
	HillSlope					
	EC50					
	amount at 10-6M					
**Plate #'s**		**5a-pregnan-17a-ol-3,20-dione**	**17a-Hydroxyprogesterone**	**17OH-pregnenolone**	**pregnenolone**	**progesterone**
AQ558	Bottom					
AQ559	Top		2.20%	3.41%	1.46%	11.74%
	LogEC50					
	HillSlope					
	EC50					
	amount at 10-6M		7.76%	1.67%	-0.02%	11.22%
**Plate #'s**		**5a-pregnan-17a-ol-3,20-dione**	**17a-Hydroxyprogesterone**	**17OH-pregnenolone**	**pregnenolone**	**progesterone**
AQ578	Bottom					
AQ579	Top					
	LogEC50					
	HillSlope					
	EC50					
	amount at 10-6M					
**Plate #'s**		**5a-pregnan-17a-ol-3,20-dione**	**17a-Hydroxyprogesterone**	**17OH-pregnenolone**	**pregnenolone**	**progesterone**
AQ595	Bottom					
AQ597	Top		30.16%			
	LogEC50					
	HillSlope					
	EC50					
	amount at 10-6M		1.61%			
**Plate #'s**		**5a-pregnan-17a-ol-3,20-dione**	**17a-Hydroxyprogesterone**	**17OH-pregnenolone**	**pregnenolone**	**progesterone**
AQ636	Bottom					
	Top					
	LogEC50					
	HillSlope					
	EC50					
	amount at 10-6M					

Plate #'s		5a-pregnan-17a-ol-3,20-dione	17a-Hydroxyprogesterone	17OH-pregnenolone	pregnenolone	progesterone
AQ641	Bottom					
	Top					
	LogEC50					
	HillSlope					
	EC50					
	amount at 10-6M					

Plate #'s		5a-pregnan-17a-ol-3,20-dione	17a-Hydroxyprogesterone	17OH-pregnenolone	pregnenolone	progesterone
AQ662	Bottom					
AQ664	Top		-0.04%			
	LogEC50					
	HillSlope					
	EC50					
	amount at 10-6M		-0.41%			

Plate #'s		5a-pregnan-17a-ol-3,20-dione	17a-Hydroxyprogesterone	17OH-pregnenolone	pregnenolone	progesterone
AQ694	Bottom					
AQ696	Top	17.84%				
	LogEC50					
	HillSlope					
	EC50					
	amount at 10-6M	12.01%				

Plate #'s		5a-pregnan-17a-ol-3,20-dione	17a-Hydroxyprogesterone	17OH-pregnenolone	pregnenolone	progesterone
AQ706	Bottom					
	Top	1.08%				
	LogEC50					
	HillSlope					
	EC50					
	amount at 10-6M	0.27%				

Plate #'s		5a-pregnan-17a-ol-3,20-dione	17a-Hydroxyprogesterone	17OH-pregnenolone	pregnenolone	progesterone
AQ745	Bottom					
AQ746	Top	-0.86%	2.83%			
	LogEC50					
	HillSlope					
	EC50					
	amount at 10-6M	-0.09%	2.64%			

Plate #'s		5a-pregnan-17a-ol-3,20-dione	17a-Hydroxyprogesterone	17OH-pregnenolone	pregnenolone	progesterone
AQ295	Bottom					
	Top					
	LogEC50					
	HillSlope					
	EC50					
	amount at 10-6M					

Plate #'s		5a-pregnan-17a-ol-3,20-dione	17a-Hydroxyprogesterone	17OH-pregnenolone	pregnenolone	progesterone
AQ296	Bottom					
	Top					
	LogEC50					
	HillSlope					
	EC50					
	amount at 10-6M					

Plate #'s		5a-pregnan-17a-ol-3,20-dione	17a-Hydroxyprogesterone	17OH-pregnenolone	pregnenolone	progesterone
AQ897	Bottom					
	Top					
	LogEC50					
	HillSlope					
	EC50					
	amount at 10-6M					

Plate #'s		5a-pregnan-17a-ol-3,20-dione	17a-Hydroxyprogesterone	17OH-pregnenolone	pregnenolone	progesterone
AQ1347	Bottom					
AQ1348	Top					
AQ1349	LogEC50					
AQ1350	HillSlope					
	EC50					
	amount at 10-6M					

Plate #'s		5a-pregnan-17a-ol-3,20-dione	17a-Hydroxyprogesterone	17OH-pregnenolone	pregnenolone	progesterone
AQ1355	Bottom					
AQ1356	Top					
AQ1357	LogEC50					
AQ1358	HillSlope					
	EC50					
	amount at 10-6M					

Plate #'s		5a-pregnan-17a-ol-3,20-dione	17a-Hydroxyprogesterone	17OH-pregnenolone	pregnenolone	progesterone
AQ1360	Bottom					
AQ1361	Top					
AQ1362	LogEC50					
AQ1363	HillSlope					
	EC50					
	amount at 10-6M					
**Plate #'s**		**5a-pregnan-17a-ol-3,20-dione**	**17a-Hydroxyprogesterone**	**17OH-pregnenolone**	**pregnenolone**	**progesterone**
AQ301	Bottom					
	Top					
	LogEC50					
	HillSlope					
	EC50					
	amount at 10-6M					
**Plate #'s**		**5a-pregnan-17a-ol-3,20-dione**	**17a-Hydroxyprogesterone**	**17OH-pregnenolone**	**pregnenolone**	**progesterone**
AQ312	Bottom					
	Top					
	LogEC50					
	HillSlope					
	EC50					
	amount at 10-6M	1.72%				
**Plate #'s**		**5a-pregnan-17a-ol-3,20-dione**	**17a-Hydroxyprogesterone**	**17OH-pregnenolone**	**pregnenolone**	**progesterone**
AQ352	Bottom					
	Top					16.48%
	LogEC50					
	HillSlope					
	EC50					
	amount at 10-6M		4.63%	3.75%	5.97%	12.64%
**Plate #'s**		**5a-pregnan-17a-ol-3,20-dione**	**17a-Hydroxyprogesterone**	**17OH-pregnenolone**	**pregnenolone**	**progesterone**
AQ273	Bottom					
	Top					
	LogEC50					
	HillSlope					
	EC50					
	amount at 10-6M					

Plate #'s		5a-pregnan-17a-ol-3,20-dione	17a-Hydroxyprogesterone	17OH-pregnenolone	pregnenolone	progesterone
AQ327	Bottom					
	Top					
	LogEC50					
	HillSlope					
	EC50					
	amount at 10-6M	0.84%				
**Plate #'s**		**5a-pregnan-17a-ol-3,20-dione**	**17a-Hydroxyprogesterone**	**17OH-pregnenolone**	**pregnenolone**	**progesterone**
AQ290	Bottom					
	Top				1.09%	13.77%
	LogEC50					
	HillSlope					
	EC50					
	amount at 10-6M		3.57%	2.15%	5.31%	13.23%
**Plate #'s**		**5a-pregnan-17a-ol-3,20-dione**	**17a-Hydroxyprogesterone**	**17OH-pregnenolone**	**pregnenolone**	**progesterone**
AQ711	Bottom					
	Top					
	LogEC50					
	HillSlope					
	EC50					
	amount at 10-6M					
**Plate #'s**		**5a-pregnan-17a-ol-3,20-dione**	**17a-Hydroxyprogesterone**	**17OH-pregnenolone**	**pregnenolone**	**progesterone**
AQ733	Bottom					
	Top					
	LogEC50					
	HillSlope					
	EC50					
	amount at 10-6M	2.78%				
**Plate #'s**		**5a-pregnan-17a-ol-3,20-dione**	**17a-Hydroxyprogesterone**	**17OH-pregnenolone**	**pregnenolone**	**progesterone**
AQ735	Bottom					
	Top					21.38%
	LogEC50					
	HillSlope					
	EC50					
	amount at 10-6M		10.77%	6.58%	6.10%	16.61%

	C15	C16	C17	C18	C19	E20	E21	E22
	11-dexoycorticosterone	11-deoxycortisol	Corticosterone	Aldosterone	Cortisol	Estradiol	Estrone	Estriol
	9.49%	5.41%	5.30%	2.94%	1.76%	38.65%	3.78%	-0.43%
**avg % of Teststerone at max**								
**sd % of Teststerone at max**								
**n % of Teststerone at max**								
**min % of Teststerone at max**								
**max % of Teststerone at max**								
**avg % of T amount at 10-6M**	9.49%	5.41%	5.30%	2.94%	1.76%	38.65%	3.78%	-0.43%
**sd % of T amount at 10-6M**	4.74%	2.35%	0.92%	2.31%	2.48%	15.68%	2.52%	1.46%
**n % of T amount at 10-6M**	4	4	4	4	4	7	4	8
**min % of T amount at 10-6M**	4.90%	3.25%	4.15%	0.81%	-0.95%	19.74%	1.50%	-3.15%
**max % of T amount at 10-6M**	15.54%	7.49%	6.05%	6.11%	4.80%	59.57%	7.36%	1.59%

**Plate #'s**		**11-dexoycorticosterone**	**11-deoxycortisol**	**Corticosterone**	**Aldosterone**	**Cortisol**	**Estradiol**	**Estrone**	**Estriol**
AQ55	Bottom								
AQ56	Top								
	LogEC50								
	HillSlope								
	EC50								
	amount at 10-6M								

**Plate #'s**		**11-dexoycorticosterone**	**11-deoxycortisol**	**Corticosterone**	**Aldosterone**	**Cortisol**	**Estradiol**	**Estrone**	**Estriol**
AQ286	Bottom								
AQ287	Top								
	LogEC50								
	HillSlope								
	EC50								
	amount at 10-6M								

**Plate #'s**		**11-dexoycorticosterone**	**11-deoxycortisol**	**Corticosterone**	**Aldosterone**	**Cortisol**	**Estradiol**	**Estrone**	**Estriol**
AQ520	Bottom								
AQ521	Top								
	LogEC50								
	HillSlope								
	EC50								
	amount at 10-6M								
**Plate #'s**		**11-dexoycorticosterone**	**11-deoxycortisol**	**Corticosterone**	**Aldosterone**	**Cortisol**	**Estradiol**	**Estrone**	**Estriol**
AQ558	Bottom								
AQ559	Top	6.82%	3.65%	6.62%	6.12%	3.14%			
	LogEC50								
	HillSlope								
	EC50								
	amount at 10-6M	6.70%	3.25%	6.03%	6.11%	2.54%			
**Plate #'s**		**11-dexoycorticosterone**	**11-deoxycortisol**	**Corticosterone**	**Aldosterone**	**Cortisol**	**Estradiol**	**Estrone**	**Estriol**
AQ578	Bottom								
AQ579	Top						1189.33%	3.39%	
	LogEC50								
	HillSlope								
	EC50								
	amount at 10-6M						53.30%	3.43%	
**Plate #'s**		**11-dexoycorticosterone**	**11-deoxycortisol**	**Corticosterone**	**Aldosterone**	**Cortisol**	**Estradiol**	**Estrone**	**Estriol**
AQ595	Bottom								
AQ597	Top								
	LogEC50								
	HillSlope								
	EC50								
	amount at 10-6M								
**Plate #'s**		**11-dexoycorticosterone**	**11-deoxycortisol**	**Corticosterone**	**Aldosterone**	**Cortisol**	**Estradiol**	**Estrone**	**Estriol**
AQ636	Bottom								
	Top								
	LogEC50								
	HillSlope								
	EC50								
	amount at 10-6M								

Plate #'s		11-dexoycorticosterone	11-deoxycortisol	Corticosterone	Aldosterone	Cortisol	Estradiol	Estrone	Estriol
AQ641	Bottom								
	Top								
	LogEC50								
	HillSlope								
	EC50								
	amount at 10-6M								

Plate #'s		11-dexoycorticosterone	11-deoxycortisol	Corticosterone	Aldosterone	Cortisol	Estradiol	Estrone	Estriol
AQ662	Bottom								
AQ664	Top								
	LogEC50								
	HillSlope								
	EC50								
	amount at 10-6M								

Plate #'s		11-dexoycorticosterone	11-deoxycortisol	Corticosterone	Aldosterone	Cortisol	Estradiol	Estrone	Estriol
AQ694	Bottom								
AQ696	Top								
	LogEC50								
	HillSlope								
	EC50								
	amount at 10-6M								

Plate #'s		11-dexoycorticosterone	11-deoxycortisol	Corticosterone	Aldosterone	Cortisol	Estradiol	Estrone	Estriol
AQ706	Bottom								
	Top				8.60%				-2.87%
	LogEC50								
	HillSlope								
	EC50								
	amount at 10-6M								-0.77%

Plate #'s		11-dexoycorticosterone	11-deoxycortisol	Corticosterone	Aldosterone	Cortisol	Estradiol	Estrone	Estriol
AQ745	Bottom								
AQ746	Top								0.02%
	LogEC50								
	HillSlope								
	EC50								
	amount at 10-6M								-0.15%

Plate #'s		11-dexoycorticosterone	11-deoxycortisol	Corticosterone	Aldosterone	Cortisol	Estradiol	Estrone	Estriol
AQ295	Bottom								
	Top								
	LogEC50								
	HillSlope								
	EC50								
	amount at 10-6M								
**Plate #'s**		**11-dexoycorticosterone**	**11-deoxycortisol**	**Corticosterone**	**Aldosterone**	**Cortisol**	**Estradiol**	**Estrone**	**Estriol**
AQ296	Bottom								
	Top								
	LogEC50								
	HillSlope								
	EC50								
	amount at 10-6M								
**Plate #'s**		**11-dexoycorticosterone**	**11-deoxycortisol**	**Corticosterone**	**Aldosterone**	**Cortisol**	**Estradiol**	**Estrone**	**Estriol**
AQ897	Bottom								
	Top								
	LogEC50								
	HillSlope								
	EC50								
	amount at 10-6M								
**Plate #'s**		**11-dexoycorticosterone**	**11-deoxycortisol**	**Corticosterone**	**Aldosterone**	**Cortisol**	**Estradiol**	**Estrone**	**Estriol**
AQ1347	Bottom								
AQ1348	Top						20.07%		-2.30%
AQ1349	LogEC50								
AQ1350	HillSlope								
	EC50								
	amount at 10-6M						19.74%		-1.55%
**Plate #'s**		**11-dexoycorticosterone**	**11-deoxycortisol**	**Corticosterone**	**Aldosterone**	**Cortisol**	**Estradiol**	**Estrone**	**Estriol**
AQ1355	Bottom								
AQ1356	Top						1453.48%		3.44%
AQ1357	LogEC50								
AQ1358	HillSlope								
	EC50								
	amount at 10-6M						51.61%		1.59%

Plate #'s		11-dexoycorticosterone	11-deoxycortisol	Corticosterone	Aldosterone	Cortisol	Estradiol	Estrone	Estriol
AQ1360	Bottom								
AQ1361	Top						3084.18%		-1.56%
AQ1362	LogEC50								
AQ1363	HillSlope								
	EC50								
	amount at 10-6M						59.57%		-3.15%
**Plate #'s**		**11-dexoycorticosterone**	**11-deoxycortisol**	**Corticosterone**	**Aldosterone**	**Cortisol**	**Estradiol**	**Estrone**	**Estriol**
AQ301	Bottom								
	Top								
	LogEC50								
	HillSlope								
	EC50								
	amount at 10-6M				1.75%				
**Plate #'s**		**11-dexoycorticosterone**	**11-deoxycortisol**	**Corticosterone**	**Aldosterone**	**Cortisol**	**Estradiol**	**Estrone**	**Estriol**
AQ312	Bottom								
	Top		8.69%	5.75%			31.61%	8.08%	
	LogEC50								
	HillSlope								
	EC50								
	amount at 10-6M		7.49%	4.96%		-0.95%	30.50%	7.36%	0.53%
**Plate #'s**		**11-dexoycorticosterone**	**11-deoxycortisol**	**Corticosterone**	**Aldosterone**	**Cortisol**	**Estradiol**	**Estrone**	**Estriol**
AQ352	Bottom								
	Top	5.19%							
	LogEC50								
	HillSlope								
	EC50								
	amount at 10-6M	4.90%							
**Plate #'s**		**11-dexoycorticosterone**	**11-deoxycortisol**	**Corticosterone**	**Aldosterone**	**Cortisol**	**Estradiol**	**Estrone**	**Estriol**
AQ273	Bottom								
	Top				3.16%				
	LogEC50								
	HillSlope								
	EC50								
	amount at 10-6M				3.10%				

**Plate #'s**		**11-dexoycorticosterone**	**11-deoxycortisol**	**Corticosterone**	**Aldosterone**	**Cortisol**	**Estradiol**	**Estrone**	**Estriol**
AQ327	Bottom								
	Top		3.51%	4.53%			29.77%	2.82%	1.49%
	LogEC50								
	HillSlope								
	EC50								
	amount at 10-6M		3.51%	4.15%		0.66%	27.79%	2.82%	-0.52%
**Plate #'s**		**11-dexoycorticosterone**	**11-deoxycortisol**	**Corticosterone**	**Aldosterone**	**Cortisol**	**Estradiol**	**Estrone**	**Estriol**
AQ290	Bottom								
	Top								
	LogEC50								
	HillSlope								
	EC50								
	amount at 10-6M	10.83%							
**Plate #'s**		**11-dexoycorticosterone**	**11-deoxycortisol**	**Corticosterone**	**Aldosterone**	**Cortisol**	**Estradiol**	**Estrone**	**Estriol**
AQ711	Bottom								
	Top								
	LogEC50								
	HillSlope								
	EC50								
	amount at 10-6M				0.81%				
**Plate #'s**		**11-dexoycorticosterone**	**11-deoxycortisol**	**Corticosterone**	**Aldosterone**	**Cortisol**	**Estradiol**	**Estrone**	**Estriol**
AQ733	Bottom								
	Top		7.78%	8.35%		10.00%		1.56%	
	LogEC50								
	HillSlope								
	EC50								
	amount at 10-6M		7.41%	6.05%		4.80%		1.50%	0.62%
**Plate #'s**		**11-dexoycorticosterone**	**11-deoxycortisol**	**Corticosterone**	**Aldosterone**	**Cortisol**	**Estradiol**	**Estrone**	**Estriol**
AQ735	Bottom								
	Top	15.95%					30.12%		
	LogEC50								
	HillSlope								
	EC50								
	amount at 10-6M	15.54%					28.03%		

	23	AAS24	AAS25	AAS26	AAS27	AAS28	AAS29	AAS30	AAS31
	Cholesterol	THG	mibolerone	mestanolone	mesterolone	normethandrone	gestrinone	nandrolone	trenbolone
	2.77%	109.53%	108.19%	102.58%	101.82%	100.70%	98.68%	98.43%	96.74%
**avg % of Teststerone at max**		109.53%	108.19%	102.58%	101.82%	100.70%	98.68%	98.43%	96.74%
**sd % of Teststerone at max**		10.56%	8.10%	4.06%	9.23%	3.08%	19.37%	7.66%	6.87%
**n % of Teststerone at max**		2	2	7	8	2	4	7	7
**min % of Teststerone at max**		102.07%	102.46%	98.24%	86.80%	98.53%	73.64%	89.15%	87.74%
**max % of Teststerone at max**		117.00%	113.92%	110.28%	115.55%	102.88%	120.81%	110.81%	106.91%
**avg % of T amount at 10-6M**	2.77%	112.44%	115.66%	105.11%	104.80%	106.15%	101.92%	96.88%	98.36%
**sd % of T amount at 10-6M**	2.62%	10.98%	10.84%	5.51%	11.28%	1.25%	28.21%	11.62%	7.26%
**n % of T amount at 10-6M**	4	2	2	7	8	2	4	7	7
**min % of T amount at 10-6M**	-0.13%	104.67%	107.99%	100.30%	85.50%	105.27%	63.85%	83.50%	87.01%
**max % of T amount at 10-6M**	5.65%	120.20%	123.32%	115.83%	118.18%	107.03%	131.82%	114.96%	106.27%

**Plate #'s**		**Cholesterol**	**THG**	**mibolerone**	**mestanolone**	**mesterolone**	**normethandrone**	**gestrinone**	**nandrolone**	**trenbolone**
AQ55	Bottom									
AQ56	Top							98.66%	100.25%	
	LogEC50									
	HillSlope									
	EC50									
	amount at 10-6M							103.67%	101.31%	

**Plate #'s**		**Cholesterol**	**THG**	**mibolerone**	**mestanolone**	**mesterolone**	**normethandrone**	**gestrinone**	**nandrolone**	**trenbolone**
AQ286	Bottom									
AQ287	Top					94.39%		73.64%	94.59%	
	LogEC50									
	HillSlope									
	EC50									
	amount at 10-6M					102.77%		63.85%	83.50%	

**Plate #'s**		**Cholesterol**	**THG**	**mibolerone**	**mestanolone**	**mesterolone**	**normethandrone**	**gestrinone**	**nandrolone**	**trenbolone**
AQ520	Bottom									
AQ521	Top					111.99%		101.61%	110.81%	
	LogEC50									
	HillSlope									
	EC50									
	amount at 10-6M					115.02%		108.34%	114.96%	
**Plate #'s**		**Cholesterol**	**THG**	**mibolerone**	**mestanolone**	**mesterolone**	**normethandrone**	**gestrinone**	**nandrolone**	**trenbolone**
AQ558	Bottom									
AQ559	Top	3.37%								
	LogEC50									
	HillSlope									
	EC50									
	amount at 10-6M	5.65%								
**Plate #'s**		**Cholesterol**	**THG**	**mibolerone**	**mestanolone**	**mesterolone**	**normethandrone**	**gestrinone**	**nandrolone**	**trenbolone**
AQ578	Bottom									
AQ579	Top									
	LogEC50									
	HillSlope									
	EC50									
	amount at 10-6M									
**Plate #'s**		**Cholesterol**	**THG**	**mibolerone**	**mestanolone**	**mesterolone**	**normethandrone**	**gestrinone**	**nandrolone**	**trenbolone**
AQ595	Bottom									
AQ597	Top				103.93%	101.25%			98.44%	87.74%
	LogEC50									
	HillSlope									
	EC50									
	amount at 10-6M				108.83%	108.77%			104.49%	87.01%
**Plate #'s**		**Cholesterol**	**THG**	**mibolerone**	**mestanolone**	**mesterolone**	**normethandrone**	**gestrinone**	**nandrolone**	**trenbolone**
AQ636	Bottom									
	Top									
	LogEC50									
	HillSlope									
	EC50									
	amount at 10-6M									

Plate #'s		Cholesterol	THG	mibolerone	mestanolone	mesterolone	normethandrone	gestrinone	nandrolone	trenbolone
AQ641	Bottom									
	Top									
	LogEC50									
	HillSlope									
	EC50									
	amount at 10-6M									
**Plate #'s**		**Cholesterol**	**THG**	**mibolerone**	**mestanolone**	**mesterolone**	**normethandrone**	**gestrinone**	**nandrolone**	**trenbolone**
AQ662	Bottom									
AQ664	Top				110.28%	115.55%				101.88%
	LogEC50									
	HillSlope									
	EC50									
	amount at 10-6M				115.83%	118.18%				104.96%
**Plate #'s**		**Cholesterol**	**THG**	**mibolerone**	**mestanolone**	**mesterolone**	**normethandrone**	**gestrinone**	**nandrolone**	**trenbolone**
AQ694	Bottom									
AQ696	Top				101.58%	104.49%				101.47%
	LogEC50									
	HillSlope									
	EC50									
	amount at 10-6M				104.91%	109.42%				106.27%
**Plate #'s**		**Cholesterol**	**THG**	**mibolerone**	**mestanolone**	**mesterolone**	**normethandrone**	**gestrinone**	**nandrolone**	**trenbolone**
AQ706	Bottom									
	Top							120.81%		
	LogEC50									
	HillSlope									
	EC50									
	amount at 10-6M							131.82%		
**Plate #'s**		**Cholesterol**	**THG**	**mibolerone**	**mestanolone**	**mesterolone**	**normethandrone**	**gestrinone**	**nandrolone**	**trenbolone**
AQ745	Bottom									
AQ746	Top				98.47%	102.24%				94.45%
	LogEC50									
	HillSlope									
	EC50									
	amount at 10-6M				102.37%	107.60%				99.39%

Plate #'s		Cholesterol	THG	mibolerone	mestanolone	mesterolone	normethandrone	gestrinone	nandrolone	trenbolone
AQ295	Bottom									
	Top		117.00%	102.46%			98.53%			
	LogEC50									
	HillSlope									
	EC50									
	amount at 10-6M		120.20%	107.99%			105.27%			
**Plate #'s**		**Cholesterol**	**THG**	**mibolerone**	**mestanolone**	**mesterolone**	**normethandrone**	**gestrinone**	**nandrolone**	**trenbolone**
AQ296	Bottom									
	Top		102.07%	113.92%			102.88%			
	LogEC50									
	HillSlope									
	EC50									
	amount at 10-6M		104.67%	123.32%			107.03%			
**Plate #'s**		**Cholesterol**	**THG**	**mibolerone**	**mestanolone**	**mesterolone**	**normethandrone**	**gestrinone**	**nandrolone**	**trenbolone**
AQ897	Bottom									
	Top									
	LogEC50									
	HillSlope									
	EC50									
	amount at 10-6M									
**Plate #'s**		**Cholesterol**	**THG**	**mibolerone**	**mestanolone**	**mesterolone**	**normethandrone**	**gestrinone**	**nandrolone**	**trenbolone**
AQ1347	Bottom									
AQ1348	Top				98.24%	86.80%			104.75%	106.91%
AQ1349	LogEC50									
AQ1350	HillSlope									
	EC50									
	amount at 10-6M				100.30%	85.50%			100.58%	103.79%
**Plate #'s**		**Cholesterol**	**THG**	**mibolerone**	**mestanolone**	**mesterolone**	**normethandrone**	**gestrinone**	**nandrolone**	**trenbolone**
AQ1355	Bottom									
AQ1356	Top				103.51%				89.15%	90.52%
AQ1357	LogEC50									
AQ1358	HillSlope									
	EC50									
	amount at 10-6M				101.61%				88.16%	92.12%

Plate #'s		Cholesterol	THG	mibolerone	mestanolone	mesterolone	normethandrone	gestrinone	nandrolone	trenbolone
AQ1360	Bottom									
AQ1361	Top				102.06%	97.85%			91.04%	94.20%
AQ1362	LogEC50									
AQ1363	HillSlope									
	EC50									
	amount at 10-6M				101.90%	91.16%			85.16%	94.99%
**Plate #'s**		**Cholesterol**	**THG**	**mibolerone**	**mestanolone**	**mesterolone**	**normethandrone**	**gestrinone**	**nandrolone**	**trenbolone**
AQ301	Bottom									
	Top									
	LogEC50									
	HillSlope									
	EC50									
	amount at 10-6M	1.39%								
**Plate #'s**		**Cholesterol**	**THG**	**mibolerone**	**mestanolone**	**mesterolone**	**normethandrone**	**gestrinone**	**nandrolone**	**trenbolone**
AQ312	Bottom									
	Top									
	LogEC50									
	HillSlope									
	EC50									
	amount at 10-6M									
**Plate #'s**		**Cholesterol**	**THG**	**mibolerone**	**mestanolone**	**mesterolone**	**normethandrone**	**gestrinone**	**nandrolone**	**trenbolone**
AQ352	Bottom									
	Top									
	LogEC50									
	HillSlope									
	EC50									
	amount at 10-6M									
**Plate #'s**		**Cholesterol**	**THG**	**mibolerone**	**mestanolone**	**mesterolone**	**normethandrone**	**gestrinone**	**nandrolone**	**trenbolone**
AQ273	Bottom									
	Top									
	LogEC50									
	HillSlope									
	EC50									
	amount at 10-6M	4.16%								

Plate #'s		Cholesterol	THG	mibolerone	mestanolone	mesterolone	normethandrone	gestrinone	nandrolone	trenbolone
AQ327	Bottom									
	Top									
	LogEC50									
	HillSlope									
	EC50									
	amount at 10-6M									
**Plate #'s**		**Cholesterol**	**THG**	**mibolerone**	**mestanolone**	**mesterolone**	**normethandrone**	**gestrinone**	**nandrolone**	**trenbolone**
AQ290	Bottom									
	Top									
	LogEC50									
	HillSlope									
	EC50									
	amount at 10-6M									
**Plate #'s**		**Cholesterol**	**THG**	**mibolerone**	**mestanolone**	**mesterolone**	**normethandrone**	**gestrinone**	**nandrolone**	**trenbolone**
AQ711	Bottom									
	Top									
	LogEC50									
	HillSlope									
	EC50									
	amount at 10-6M									
**Plate #'s**		**Cholesterol**	**THG**	**mibolerone**	**mestanolone**	**mesterolone**	**normethandrone**	**gestrinone**	**nandrolone**	**trenbolone**
AQ733	Bottom									
	Top	0.60%								
	LogEC50									
	HillSlope									
	EC50									
	amount at 10-6M	-0.13%								
**Plate #'s**		**Cholesterol**	**THG**	**mibolerone**	**mestanolone**	**mesterolone**	**normethandrone**	**gestrinone**	**nandrolone**	**trenbolone**
AQ735	Bottom									
	Top									
	LogEC50									
	HillSlope									
	EC50									
	amount at 10-6M									

	AAS32	AAS33	AAS34	AAS35	AAS36	AAS37	AAS38	AAS39
	tibolone	methyltestosterone	stanozolol	fluoxymesterone	oxymethalone	danazol	19-norandrostenedione	boldione
	96.12%	91.95%	87.75%	83.00%	76.93%	67.54%	45.36%	25.27%
avg % of Teststerone at max	96.12%	91.95%	87.75%	83.00%	76.93%	67.54%		
sd % of Teststerone at max	6.38%	4.69%	11.87%	9.07%	5.05%	6.40%		
n % of Teststerone at max	4	7	5	6	2	7		
min % of Teststerone at max	86.56%	82.43%	76.35%	68.70%	73.36%	61.30%		
max % of Teststerone at max	99.61%	97.04%	105.74%	95.31%	80.51%	77.10%		
avg % of T amount at 10-6M	90.84%	94.48%	89.82%	81.13%	77.14%	64.65%	45.36%	25.27%
sd % of T amount at 10-6M	12.76%	6.46%	13.67%	9.13%	3.29%	6.43%	1.54%	10.40%
n % of T amount at 10-6M	4	7	5	6	2	7	4	7
min % of T amount at 10-6M	73.87%	82.88%	74.72%	68.09%	74.81%	52.49%	43.07%	12.32%
max % of T amount at 10-6M	102.25%	101.31%	110.28%	94.00%	79.47%	71.68%	46.36%	43.79%

Plate #'s		tibolone	methyltestosterone	stanozolol	fluoxymesterone	oxymethalone	danazol	19-norandrostenedione	boldione
AQ55	Bottom								
AQ56	Top						62.70%	54.89%	522.94%
	LogEC50								
	HillSlope								
	EC50								
	amount at 10-6M						61.50%	46.36%	17.62%

Plate #'s		tibolone	methyltestosterone	stanozolol	fluoxymesterone	oxymethalone	danazol	19-norandrostenedione	boldione
AQ286	Bottom								
AQ287	Top			83.96%			61.30%	157.63%	474.86%
	LogEC50								
	HillSlope								
	EC50								
	amount at 10-6M			92.15%			52.49%	43.07%	28.57%

Plate #'s		tibolone	methyltestosterone	stanozolol	fluoxymesterone	oxymethalone	danazol	19-norandrostenedione	boldione
AQ520	Bottom								
AQ521	Top			105.74%			70.27%	69.21%	26.25%
	LogEC50								
	HillSlope								
	EC50								
	amount at 10-6M			110.28%			70.17%	45.89%	18.41%
**Plate #'s**		**tibolone**	**methyltestosterone**	**stanozolol**	**fluoxymesterone**	**oxymethalone**	**danazol**	**19-norandrostenedione**	**boldione**
AQ558	Bottom								
AQ559	Top								
	LogEC50								
	HillSlope								
	EC50								
	amount at 10-6M								
**Plate #'s**		**tibolone**	**methyltestosterone**	**stanozolol**	**fluoxymesterone**	**oxymethalone**	**danazol**	**19-norandrostenedione**	**boldione**
AQ578	Bottom								
AQ579	Top				84.85%				
	LogEC50								
	HillSlope								
	EC50								
	amount at 10-6M				85.90%				
**Plate #'s**		**tibolone**	**methyltestosterone**	**stanozolol**	**fluoxymesterone**	**oxymethalone**	**danazol**	**19-norandrostenedione**	**boldione**
AQ595	Bottom								
AQ597	Top	99.61%	93.50%				62.63%		
	LogEC50								
	HillSlope								
	EC50								
	amount at 10-6M	102.25%	101.31%				67.61%		
**Plate #'s**		**tibolone**	**methyltestosterone**	**stanozolol**	**fluoxymesterone**	**oxymethalone**	**danazol**	**19-norandrostenedione**	**boldione**
AQ636	Bottom								
	Top								
	LogEC50								
	HillSlope								
	EC50								
	amount at 10-6M								

**Plate #'s**		**tibolone**	**methyltestosterone**	**stanozolol**	**fluoxymesterone**	**oxymethalone**	**danazol**	**19-norandrostenedione**	**boldione**
AQ641	Bottom								
	Top								
	LogEC50								
	HillSlope								
	EC50								
	amount at 10-6M								
**Plate #'s**		**tibolone**	**methyltestosterone**	**stanozolol**	**fluoxymesterone**	**oxymethalone**	**danazol**	**19-norandrostenedione**	**boldione**
AQ662	Bottom								
AQ664	Top		93.18%		78.43%				
	LogEC50								
	HillSlope								
	EC50								
	amount at 10-6M		89.14%		74.72%				
**Plate #'s**		**tibolone**	**methyltestosterone**	**stanozolol**	**fluoxymesterone**	**oxymethalone**	**danazol**	**19-norandrostenedione**	**boldione**
AQ694	Bottom								
AQ696	Top		95.13%		82.21%				
	LogEC50								
	HillSlope								
	EC50								
	amount at 10-6M		98.36%		79.26%				
**Plate #'s**		**tibolone**	**methyltestosterone**	**stanozolol**	**fluoxymesterone**	**oxymethalone**	**danazol**	**19-norandrostenedione**	**boldione**
AQ706	Bottom								
	Top				95.31%				32.77%
	LogEC50								
	HillSlope								
	EC50								
	amount at 10-6M				94.00%				29.78%
**Plate #'s**		**tibolone**	**methyltestosterone**	**stanozolol**	**fluoxymesterone**	**oxymethalone**	**danazol**	**19-norandrostenedione**	**boldione**
AQ745	Bottom								
AQ746	Top		91.02%		68.70%				
	LogEC50								
	HillSlope								
	EC50								
	amount at 10-6M		93.87%		68.09%				

Plate #'s		tibolone	methyltestosterone	stanozolol	fluoxymesterone	oxymethalone	danazol	19-norandrostenedione	boldione
AQ295	Bottom								
	Top					80.51%			
	LogEC50								
	HillSlope								
	EC50								
	amount at 10-6M					79.47%			
**Plate #'s**		**tibolone**	**methyltestosterone**	**stanozolol**	**fluoxymesterone**	**oxymethalone**	**danazol**	**19-norandrostenedione**	**boldione**
AQ296	Bottom								
	Top					73.36%			
	LogEC50								
	HillSlope								
	EC50								
	amount at 10-6M					74.81%			
**Plate #'s**		**tibolone**	**methyltestosterone**	**stanozolol**	**fluoxymesterone**	**oxymethalone**	**danazol**	**19-norandrostenedione**	**boldione**
AQ897	Bottom								
	Top								
	LogEC50								
	HillSlope								
	EC50								
	amount at 10-6M								
**Plate #'s**		**tibolone**	**methyltestosterone**	**stanozolol**	**fluoxymesterone**	**oxymethalone**	**danazol**	**19-norandrostenedione**	**boldione**
AQ1347	Bottom								
AQ1348	Top	99.49%	91.32%	76.35%	88.46%		77.10%	0.00%	345.71%
AQ1349	LogEC50								
AQ1350	HillSlope								
	EC50								
	amount at 10-6M	73.87%	98.89%	74.72%	84.80%		65.33%	46.13%	43.79%
**Plate #'s**		**tibolone**	**methyltestosterone**	**stanozolol**	**fluoxymesterone**	**oxymethalone**	**danazol**	**19-norandrostenedione**	**boldione**
AQ1355	Bottom								
AQ1356	Top	86.56%	82.43%	79.58%			64.20%		15.18%
AQ1357	LogEC50								
AQ1358	HillSlope								
	EC50								
	amount at 10-6M	88.39%	82.88%	80.22%			63.75%		12.32%

Plate #'s		tibolone	methyltestosterone	stanozolol	fluoxymesterone	oxymethalone	danazol	19-norandrostenedione	boldione
AQ1360	Bottom								
AQ1361	Top	98.82%	97.04%	93.12%			74.54%		34.87%
AQ1362	LogEC50								
AQ1363	HillSlope								
	EC50								
	amount at 10-6M	98.87%	96.90%	91.74%			71.68%		26.41%

Plate #'s		tibolone	methyltestosterone	stanozolol	fluoxymesterone	oxymethalone	danazol	19-norandrostenedione	boldione
AQ301	Bottom								
	Top								
	LogEC50								
	HillSlope								
	EC50								
	amount at 10-6M								

Plate #'s		tibolone	methyltestosterone	stanozolol	fluoxymesterone	oxymethalone	danazol	19-norandrostenedione	boldione
AQ312	Bottom								
	Top								
	LogEC50								
	HillSlope								
	EC50								
	amount at 10-6M								

Plate #'s		tibolone	methyltestosterone	stanozolol	fluoxymesterone	oxymethalone	danazol	19-norandrostenedione	boldione
AQ352	Bottom								
	Top								
	LogEC50								
	HillSlope								
	EC50								
	amount at 10-6M								

Plate #'s		tibolone	methyltestosterone	stanozolol	fluoxymesterone	oxymethalone	danazol	19-norandrostenedione	boldione
AQ273	Bottom								
	Top								
	LogEC50								
	HillSlope								
	EC50								
	amount at 10-6M								

Plate #'s		tibolone	methyltestosterone	stanozolol	fluoxymesterone	oxymethalone	danazol	19-norandrostenedione	boldione
AQ327	Bottom								
	Top								
	LogEC50								
	HillSlope								
	EC50								
	amount at 10-6M								
**Plate #'s**		**tibolone**	**methyltestosterone**	**stanozolol**	**fluoxymesterone**	**oxymethalone**	**danazol**	**19-norandrostenedione**	**boldione**
AQ290	Bottom								
	Top								
	LogEC50								
	HillSlope								
	EC50								
	amount at 10-6M								
**Plate #'s**		**tibolone**	**methyltestosterone**	**stanozolol**	**fluoxymesterone**	**oxymethalone**	**danazol**	**19-norandrostenedione**	**boldione**
AQ711	Bottom								
	Top								
	LogEC50								
	HillSlope								
	EC50								
	amount at 10-6M								
**Plate #'s**		**tibolone**	**methyltestosterone**	**stanozolol**	**fluoxymesterone**	**oxymethalone**	**danazol**	**19-norandrostenedione**	**boldione**
AQ733	Bottom								
	Top								
	LogEC50								
	HillSlope								
	EC50								
	amount at 10-6M								
**Plate #'s**		**tibolone**	**methyltestosterone**	**stanozolol**	**fluoxymesterone**	**oxymethalone**	**danazol**	**19-norandrostenedione**	**boldione**
AQ735	Bottom								
	Top								
	LogEC50								
	HillSlope								
	EC50								
	amount at 10-6M								

	m26/33a	m26/33b	m27
	17a-methyl-5a-androstane-3a,17b-diol	17a-methyl-5b-androstane-3a,17b-diol	1a-methyl-5a-androstane-3a-ol-17-one
	77.65%	-0.53%	8.47%
**avg % of Teststerone at max**	77.65%		
**sd % of Teststerone at max**	23.24%		
**n % of Teststerone at max**	3		
**min % of Teststerone at max**	56.27%		
**max % of Teststerone at max**	102.39%		
**avg % of T amount at 10-6M**	71.02%	-0.53%	8.47%
**sd % of T amount at 10-6M**	16.87%	1.02%	5.35%
**n % of T amount at 10-6M**	3	3	3
**min % of T amount at 10-6M**	54.00%	-1.59%	3.34%
**max % of T amount at 10-6M**	87.73%	0.44%	14.02%

**Plate #'s**		17a-methyl-5a-androstane-3a,17b-diol	17a-methyl-5b-androstane-3a,17b-diol	1a-methyl-5a-androstane-3a-ol-17-one
AQ55	Bottom			
AQ56	Top			
	LogEC50			
	HillSlope			
	EC50			
	amount at 10-6M			
**Plate #'s**		17a-methyl-5a-androstane-3a,17b-diol	17a-methyl-5b-androstane-3a,17b-diol	1a-methyl-5a-androstane-3a-ol-17-one
AQ286	Bottom			
AQ287	Top			
	LogEC50			
	HillSlope			
	EC50			
	amount at 10-6M			

Plate #'s		17a-methyl-5a-androstane-3a,17b-diol	17a-methyl-5b-androstane-3a,17b-diol	1a-methyl-5a-androstane-3a-ol-17-one
AQ520	Bottom			
AQ521	Top			
	LogEC50			
	HillSlope			
	EC50			
	amount at 10-6M			
**Plate #'s**		**17a-methyl-5a-androstane-3a,17b-diol**	**17a-methyl-5b-androstane-3a,17b-diol**	**1a-methyl-5a-androstane-3a-ol-17-one**
AQ558	Bottom			
AQ559	Top			
	LogEC50			
	HillSlope			
	EC50			
	amount at 10-6M			
**Plate #'s**		**17a-methyl-5a-androstane-3a,17b-diol**	**17a-methyl-5b-androstane-3a,17b-diol**	**1a-methyl-5a-androstane-3a-ol-17-one**
AQ578	Bottom			
AQ579	Top			
	LogEC50			
	HillSlope			
	EC50			
	amount at 10-6M			
**Plate #'s**		**17a-methyl-5a-androstane-3a,17b-diol**	**17a-methyl-5b-androstane-3a,17b-diol**	**1a-methyl-5a-androstane-3a-ol-17-one**
AQ595	Bottom			
AQ597	Top			
	LogEC50			
	HillSlope			
	EC50			
	amount at 10-6M			
**Plate #'s**		**17a-methyl-5a-androstane-3a,17b-diol**	**17a-methyl-5b-androstane-3a,17b-diol**	**1a-methyl-5a-androstane-3a-ol-17-one**
AQ636	Bottom			
	Top			
	LogEC50			
	HillSlope			
	EC50			
	amount at 10-6M			

Plate #'s		17a-methyl-5a-androstane-3a,17b-diol	17a-methyl-5b-androstane-3a,17b-diol	1a-methyl-5a-androstane-3a-ol-17-one
AQ641	Bottom			
	Top			
	LogEC50			
	HillSlope			
	EC50			
	amount at 10-6M			

Plate #'s		17a-methyl-5a-androstane-3a,17b-diol	17a-methyl-5b-androstane-3a,17b-diol	1a-methyl-5a-androstane-3a-ol-17-one
AQ662	Bottom			
AQ664	Top			
	LogEC50			
	HillSlope			
	EC50			
	amount at 10-6M			

Plate #'s		17a-methyl-5a-androstane-3a,17b-diol	17a-methyl-5b-androstane-3a,17b-diol	1a-methyl-5a-androstane-3a-ol-17-one
AQ694	Bottom			
AQ696	Top			
	LogEC50			
	HillSlope			
	EC50			
	amount at 10-6M			

Plate #'s		17a-methyl-5a-androstane-3a,17b-diol	17a-methyl-5b-androstane-3a,17b-diol	1a-methyl-5a-androstane-3a-ol-17-one
AQ706	Bottom			
	Top			
	LogEC50			
	HillSlope			
	EC50			
	amount at 10-6M			

Plate #'s		17a-methyl-5a-androstane-3a,17b-diol	17a-methyl-5b-androstane-3a,17b-diol	1a-methyl-5a-androstane-3a-ol-17-one
AQ745	Bottom			
AQ746	Top			
	LogEC50			
	HillSlope			
	EC50			
	amount at 10-6M			

Plate #'s		17a-methyl-5a-androstane-3a,17b-diol	17a-methyl-5b-androstane-3a,17b-diol	1a-methyl-5a-androstane-3a-ol-17-one
AQ295	Bottom			
	Top			
	LogEC50			
	HillSlope			
	EC50			
	amount at 10-6M			
**Plate #'s**		**17a-methyl-5a-androstane-3a,17b-diol**	**17a-methyl-5b-androstane-3a,17b-diol**	**1a-methyl-5a-androstane-3a-ol-17-one**
AQ296	Bottom			
	Top			
	LogEC50			
	HillSlope			
	EC50			
	amount at 10-6M			
**Plate #'s**		**17a-methyl-5a-androstane-3a,17b-diol**	**17a-methyl-5b-androstane-3a,17b-diol**	**1a-methyl-5a-androstane-3a-ol-17-one**
AQ897	Bottom			
	Top			
	LogEC50			
	HillSlope			
	EC50			
	amount at 10-6M			
**Plate #'s**		**17a-methyl-5a-androstane-3a,17b-diol**	**17a-methyl-5b-androstane-3a,17b-diol**	**1a-methyl-5a-androstane-3a-ol-17-one**
AQ1347	Bottom			
AQ1348	Top	74.30%	0.00%	20.15%
AQ1349	LogEC50			
AQ1350	HillSlope			
	EC50			
	amount at 10-6M	71.32%	-0.45%	14.02%
**Plate #'s**		**17a-methyl-5a-androstane-3a,17b-diol**	**17a-methyl-5b-androstane-3a,17b-diol**	**1a-methyl-5a-androstane-3a-ol-17-one**
AQ1355	Bottom			
AQ1356	Top	56.27%	0.10%	5.51%
AQ1357	LogEC50			
AQ1358	HillSlope			
	EC50			
	amount at 10-6M	54.00%	0.44%	3.34%

Plate #'s		17a-methyl-5a-androstane-3a,17b-diol	17a-methyl-5b-androstane-3a,17b-diol	1a-methyl-5a-androstane-3a-ol-17-one
AQ1360	Bottom			
AQ1361	Top	102.39%	-2.41%	57.04%
AQ1362	LogEC50			
AQ1363	HillSlope			
	EC50			
	amount at 10-6M	87.73%	-1.59%	8.05%
**Plate #'s**		**17a-methyl-5a-androstane-3a,17b-diol**	**17a-methyl-5b-androstane-3a,17b-diol**	**1a-methyl-5a-androstane-3a-ol-17-one**
AQ301	Bottom			
	Top			
	LogEC50			
	HillSlope			
	EC50			
	amount at 10-6M			
**Plate #'s**		**17a-methyl-5a-androstane-3a,17b-diol**	**17a-methyl-5b-androstane-3a,17b-diol**	**1a-methyl-5a-androstane-3a-ol-17-one**
AQ312	Bottom			
	Top			
	LogEC50			
	HillSlope			
	EC50			
	amount at 10-6M			
**Plate #'s**		**17a-methyl-5a-androstane-3a,17b-diol**	**17a-methyl-5b-androstane-3a,17b-diol**	**1a-methyl-5a-androstane-3a-ol-17-one**
AQ352	Bottom			
	Top			
	LogEC50			
	HillSlope			
	EC50			
	amount at 10-6M			
**Plate #'s**		**17a-methyl-5a-androstane-3a,17b-diol**	**17a-methyl-5b-androstane-3a,17b-diol**	**1a-methyl-5a-androstane-3a-ol-17-one**
AQ273	Bottom			
	Top			
	LogEC50			
	HillSlope			
	EC50			
	amount at 10-6M			

Plate #'s		17a-methyl-5a-androstane-3a,17b-diol	17a-methyl-5b-androstane-3a,17b-diol	1a-methyl-5a-androstane-3a-ol-17-one
AQ327	Bottom			
	Top			
	LogEC50			
	HillSlope			
	EC50			
	amount at 10-6M			
**Plate #'s**		**17a-methyl-5a-androstane-3a,17b-diol**	**17a-methyl-5b-androstane-3a,17b-diol**	**1a-methyl-5a-androstane-3a-ol-17-one**
AQ290	Bottom			
	Top			
	LogEC50			
	HillSlope			
	EC50			
	amount at 10-6M			
**Plate #'s**		**17a-methyl-5a-androstane-3a,17b-diol**	**17a-methyl-5b-androstane-3a,17b-diol**	**1a-methyl-5a-androstane-3a-ol-17-one**
AQ711	Bottom			
	Top			
	LogEC50			
	HillSlope			
	EC50			
	amount at 10-6M			
**Plate #'s**		**17a-methyl-5a-androstane-3a,17b-diol**	**17a-methyl-5b-androstane-3a,17b-diol**	**1a-methyl-5a-androstane-3a-ol-17-one**
AQ733	Bottom			
	Top			
	LogEC50			
	HillSlope			
	EC50			
	amount at 10-6M			
**Plate #'s**		**17a-methyl-5a-androstane-3a,17b-diol**	**17a-methyl-5b-androstane-3a,17b-diol**	**1a-methyl-5a-androstane-3a-ol-17-one**
AQ735	Bottom			
	Top			
	LogEC50			
	HillSlope			
	EC50			
	amount at 10-6M			

		m30	m31	m32	m34a	m34b
		19-Norandrosterone	7a-Trenbolone	3-Hydroxytibolone mixture	16b-hydroxystanozolol	3'-OH-stanozolol
		1.53%	60.12%	9.36%	5.57%	86.46%
	**avg % of Teststerone at max**		60.12%			86.46%
	**sd % of Teststerone at max**		16.98%			22.83%
	**n % of Teststerone at max**		3			3
	**min % of Teststerone at max**		47.99%			60.40%
	**max % of Teststerone at max**		79.53%			102.95%
	**avg % of T amount at 10-6M**	1.53%	57.34%	9.36%	5.57%	58.85%
	**sd % of T amount at 10-6M**	0.60%	16.64%	0.45%	7.81%	36.49%
	**n % of T amount at 10-6M**	3	3	3	3	3
	**min % of T amount at 10-6M**	0.84%	44.63%	8.87%	-1.21%	22.17%
	**max % of T amount at 10-6M**	1.97%	76.17%	9.77%	14.10%	95.15%

Plate #'s		19-Norandrosterone	7a-Trenbolone	3-Hydroxytibolone mixture	16b-hydroxystanozolol	3'-OH-stanozolol
AQ55	Bottom					
AQ56	Top					
	LogEC50					
	HillSlope					
	EC50					
	amount at 10-6M					

Plate #'s		19-Norandrosterone	7a-Trenbolone	3-Hydroxytibolone mixture	16b-hydroxystanozolol	3'-OH-stanozolol
AQ286	Bottom					
AQ287	Top					
	LogEC50					
	HillSlope					
	EC50					
	amount at 10-6M					

Plate #'s		19-Norandrosterone	7a-Trenbolone	3-Hydroxytibolone mixture	16b-hydroxystanozolol	3'-OH-stanozolol
AQ520	Bottom					
AQ521	Top					
	LogEC50					
	HillSlope					
	EC50					
	amount at 10-6M					

Plate #'s		19-Norandrosterone	7a-Trenbolone	3-Hydroxytibolone mixture	16b-hydroxystanozolol	3'-OH-stanozolol
AQ558	Bottom					
AQ559	Top					
	LogEC50					
	HillSlope					
	EC50					
	amount at 10-6M					

Plate #'s		19-Norandrosterone	7a-Trenbolone	3-Hydroxytibolone mixture	16b-hydroxystanozolol	3'-OH-stanozolol
AQ578	Bottom					
AQ579	Top					
	LogEC50					
	HillSlope					
	EC50					
	amount at 10-6M					

Plate #'s		19-Norandrosterone	7a-Trenbolone	3-Hydroxytibolone mixture	16b-hydroxystanozolol	3'-OH-stanozolol
AQ595	Bottom					
AQ597	Top					
	LogEC50					
	HillSlope					
	EC50					
	amount at 10-6M					

Plate #'s		19-Norandrosterone	7a-Trenbolone	3-Hydroxytibolone mixture	16b-hydroxystanozolol	3'-OH-stanozolol
AQ636	Bottom					
	Top					
	LogEC50					
	HillSlope					
	EC50					
	amount at 10-6M					

Plate #'s		19-Norandrosterone	7a-Trenbolone	3-Hydroxytibolone mixture	16b-hydroxystanozolol	3'-OH-stanozolol
AQ641	Bottom					
	Top					
	LogEC50					
	HillSlope					
	EC50					
	amount at 10-6M					
**Plate #'s**		**19-Norandrosterone**	**7a-Trenbolone**	**3-Hydroxytibolone mixture**	**16b-hydroxystanozolol**	**3'-OH-stanozolol**
AQ662	Bottom					
AQ664	Top					
	LogEC50					
	HillSlope					
	EC50					
	amount at 10-6M					
**Plate #'s**		**19-Norandrosterone**	**7a-Trenbolone**	**3-Hydroxytibolone mixture**	**16b-hydroxystanozolol**	**3'-OH-stanozolol**
AQ694	Bottom					
AQ696	Top					
	LogEC50					
	HillSlope					
	EC50					
	amount at 10-6M					
**Plate #'s**		**19-Norandrosterone**	**7a-Trenbolone**	**3-Hydroxytibolone mixture**	**16b-hydroxystanozolol**	**3'-OH-stanozolol**
AQ706	Bottom					
	Top					
	LogEC50					
	HillSlope					
	EC50					
	amount at 10-6M					
**Plate #'s**		**19-Norandrosterone**	**7a-Trenbolone**	**3-Hydroxytibolone mixture**	**16b-hydroxystanozolol**	**3'-OH-stanozolol**
AQ745	Bottom					
AQ746	Top					
	LogEC50					
	HillSlope					
	EC50					
	amount at 10-6M					

Plate #'s		19-Norandrosterone	7a-Trenbolone	3-Hydroxytibolone mixture	16b-hydroxystanozolol	3'-OH-stanozolol
AQ295	Bottom					
	Top					
	LogEC50					
	HillSlope					
	EC50					
	amount at 10-6M					
**Plate #'s**		**19-Norandrosterone**	**7a-Trenbolone**	**3-Hydroxytibolone mixture**	**16b-hydroxystanozolol**	**3'-OH-stanozolol**
AQ296	Bottom					
	Top					
	LogEC50					
	HillSlope					
	EC50					
	amount at 10-6M					
**Plate #'s**		**19-Norandrosterone**	**7a-Trenbolone**	**3-Hydroxytibolone mixture**	**16b-hydroxystanozolol**	**3'-OH-stanozolol**
AQ897	Bottom					
	Top					
	LogEC50					
	HillSlope					
	EC50					
	amount at 10-6M					
**Plate #'s**		**19-Norandrosterone**	**7a-Trenbolone**	**3-Hydroxytibolone mixture**	**16b-hydroxystanozolol**	**3'-OH-stanozolol**
AQ1347	Bottom					
AQ1348	Top	2.72%	47.99%	14.14%	25.62%	102.95%
AQ1349	LogEC50					
AQ1350	HillSlope					
	EC50					
	amount at 10-6M	1.78%	44.63%	9.77%	-1.21%	22.17%
**Plate #'s**		**19-Norandrosterone**	**7a-Trenbolone**	**3-Hydroxytibolone mixture**	**16b-hydroxystanozolol**	**3'-OH-stanozolol**
AQ1355	Bottom					
AQ1356	Top	0.59%	52.85%	0.00%	15.33%	60.40%
AQ1357	LogEC50					
AQ1358	HillSlope					
	EC50					
	amount at 10-6M	1.97%	51.22%	8.87%	14.10%	59.24%

Plate #'s		19-Norandrosterone	7a-Trenbolone	3-Hydroxytibolone mixture	16b-hydroxystanozolol	3'-OH-stanozolol
AQ1360	Bottom					
AQ1361	Top	59.62%	79.53%	0.00%	7.53%	96.01%
AQ1362	LogEC50					
AQ1363	HillSlope					
	EC50					
	amount at 10-6M	0.84%	76.17%	9.43%	3.81%	95.15%
**Plate #'s**		**19-Norandrosterone**	**7a-Trenbolone**	**3-Hydroxytibolone mixture**	**16b-hydroxystanozolol**	**3'-OH-stanozolol**
AQ301	Bottom					
	Top					
	LogEC50					
	HillSlope					
	EC50					
	amount at 10-6M					
**Plate #'s**		**19-Norandrosterone**	**7a-Trenbolone**	**3-Hydroxytibolone mixture**	**16b-hydroxystanozolol**	**3'-OH-stanozolol**
AQ312	Bottom					
	Top					
	LogEC50					
	HillSlope					
	EC50					
	amount at 10-6M					
**Plate #'s**		**19-Norandrosterone**	**7a-Trenbolone**	**3-Hydroxytibolone mixture**	**16b-hydroxystanozolol**	**3'-OH-stanozolol**
AQ352	Bottom					
	Top					
	LogEC50					
	HillSlope					
	EC50					
	amount at 10-6M					
**Plate #'s**		**19-Norandrosterone**	**7a-Trenbolone**	**3-Hydroxytibolone mixture**	**16b-hydroxystanozolol**	**3'-OH-stanozolol**
AQ273	Bottom					
	Top					
	LogEC50					
	HillSlope					
	EC50					
	amount at 10-6M					

Plate #'s		19-Norandrosterone	7a-Trenbolone	3-Hydroxytibolone mixture	16b-hydroxystanozolol	3'-OH-stanozolol
AQ327	Bottom					
	Top					
	LogEC50					
	HillSlope					
	EC50					
	amount at 10-6M					

Plate #'s		19-Norandrosterone	7a-Trenbolone	3-Hydroxytibolone mixture	16b-hydroxystanozolol	3'-OH-stanozolol
AQ290	Bottom					
	Top					
	LogEC50					
	HillSlope					
	EC50					
	amount at 10-6M					

Plate #'s		19-Norandrosterone	7a-Trenbolone	3-Hydroxytibolone mixture	16b-hydroxystanozolol	3'-OH-stanozolol
AQ711	Bottom					
	Top					
	LogEC50					
	HillSlope					
	EC50					
	amount at 10-6M					

Plate #'s		19-Norandrosterone	7a-Trenbolone	3-Hydroxytibolone mixture	16b-hydroxystanozolol	3'-OH-stanozolol
AQ733	Bottom					
	Top					
	LogEC50					
	HillSlope					
	EC50					
	amount at 10-6M					

Plate #'s		19-Norandrosterone	7a-Trenbolone	3-Hydroxytibolone mixture	16b-hydroxystanozolol	3'-OH-stanozolol
AQ735	Bottom					
	Top					
	LogEC50					
	HillSlope					
	EC50					
	amount at 10-6M					

		m34c	m35	m37	m39
		4b-hydroxystanozolol	9a-fluoro-17a-methyl-androst-4-ene-a,6b,1	17a-Ethynyl-testosterone	5b-androst-1en-17b-ol-3-one
		2.20%	1.26%	34.11%	12.60%
	**avg % of Teststerone at max**			34.11%	
	**sd % of Teststerone at max**			3.23%	
	**n % of Teststerone at max**			3	
	**min % of Teststerone at max**			31.93%	
	**max % of Teststerone at max**			37.82%	
	**avg % of T amount at 10-6M**	2.20%	1.26%	33.07%	12.60%
	**sd % of T amount at 10-6M**	1.48%	1.60%	5.26%	4.04%
	**n % of T amount at 10-6M**	3	3	3	3
	**min % of T amount at 10-6M**	0.51%	0.15%	29.14%	8.25%
	**max % of T amount at 10-6M**	3.29%	3.10%	39.04%	16.22%

**Plate #'s**		**4b-hydroxystanozolol**	**9a-fluoro-17a-methyl-androst-4-ene-a,6b,1**	**17a-Ethynyl-testosterone**	**5b-androst-1en-17b-ol-3-one**
AQ55	Bottom				
AQ56	Top				
	LogEC50				
	HillSlope				
	EC50				
	amount at 10-6M				

**Plate #'s**		**4b-hydroxystanozolol**	**9a-fluoro-17a-methyl-androst-4-ene-a,6b,1**	**17a-Ethynyl-testosterone**	**5b-androst-1en-17b-ol-3-one**
AQ286	Bottom				
AQ287	Top				
	LogEC50				
	HillSlope				
	EC50				
	amount at 10-6M				

Plate #'s		4b-hydroxystanozolol	9a-fluoro-17a-methyl-androst-4-ene-a,6b,1	17a-Ethynyl-testosterone	5b-androst-1en-17b-ol-3-one
AQ520	Bottom				
AQ521	Top				
	LogEC50				
	HillSlope				
	EC50				
	amount at 10-6M				
**Plate #'s**		**4b-hydroxystanozolol**	**9a-fluoro-17a-methyl-androst-4-ene-a,6b,1**	**17a-Ethynyl-testosterone**	**5b-androst-1en-17b-ol-3-one**
AQ558	Bottom				
AQ559	Top				
	LogEC50				
	HillSlope				
	EC50				
	amount at 10-6M				
**Plate #'s**		**4b-hydroxystanozolol**	**9a-fluoro-17a-methyl-androst-4-ene-a,6b,1**	**17a-Ethynyl-testosterone**	**5b-androst-1en-17b-ol-3-one**
AQ578	Bottom				
AQ579	Top				
	LogEC50				
	HillSlope				
	EC50				
	amount at 10-6M				
**Plate #'s**		**4b-hydroxystanozolol**	**9a-fluoro-17a-methyl-androst-4-ene-a,6b,1**	**17a-Ethynyl-testosterone**	**5b-androst-1en-17b-ol-3-one**
AQ595	Bottom				
AQ597	Top				
	LogEC50				
	HillSlope				
	EC50				
	amount at 10-6M				
**Plate #'s**		**4b-hydroxystanozolol**	**9a-fluoro-17a-methyl-androst-4-ene-a,6b,1**	**17a-Ethynyl-testosterone**	**5b-androst-1en-17b-ol-3-one**
AQ636	Bottom				
	Top				
	LogEC50				
	HillSlope				
	EC50				
	amount at 10-6M				

Plate #'s		4b-hydroxystanozolol	9a-fluoro-17a-methyl-androst-4-ene-a,6b,1	17a-Ethynyl-testosterone	5b-androst-1en-17b-ol-3-one
AQ641	Bottom				
	Top				
	LogEC50				
	HillSlope				
	EC50				
	amount at 10-6M				
**Plate #'s**		**4b-hydroxystanozolol**	**9a-fluoro-17a-methyl-androst-4-ene-a,6b,1**	**17a-Ethynyl-testosterone**	**5b-androst-1en-17b-ol-3-one**
AQ662	Bottom				
AQ664	Top				
	LogEC50				
	HillSlope				
	EC50				
	amount at 10-6M				
**Plate #'s**		**4b-hydroxystanozolol**	**9a-fluoro-17a-methyl-androst-4-ene-a,6b,1**	**17a-Ethynyl-testosterone**	**5b-androst-1en-17b-ol-3-one**
AQ694	Bottom				
AQ696	Top				
	LogEC50				
	HillSlope				
	EC50				
	amount at 10-6M				
**Plate #'s**		**4b-hydroxystanozolol**	**9a-fluoro-17a-methyl-androst-4-ene-a,6b,1**	**17a-Ethynyl-testosterone**	**5b-androst-1en-17b-ol-3-one**
AQ706	Bottom				
	Top				
	LogEC50				
	HillSlope				
	EC50				
	amount at 10-6M				
**Plate #'s**		**4b-hydroxystanozolol**	**9a-fluoro-17a-methyl-androst-4-ene-a,6b,1**	**17a-Ethynyl-testosterone**	**5b-androst-1en-17b-ol-3-one**
AQ745	Bottom				
AQ746	Top				
	LogEC50				
	HillSlope				
	EC50				
	amount at 10-6M				

Plate #'s		4b-hydroxystanozolol	9a-fluoro-17a-methyl-androst-4-ene-a,6b,1	17a-Ethynyl-testosterone	5b-androst-1en-17b-ol-3-one
AQ295	Bottom				
	Top				
	LogEC50				
	HillSlope				
	EC50				
	amount at 10-6M				
**Plate #'s**		**4b-hydroxystanozolol**	**9a-fluoro-17a-methyl-androst-4-ene-a,6b,1**	**17a-Ethynyl-testosterone**	**5b-androst-1en-17b-ol-3-one**
AQ296	Bottom				
	Top				
	LogEC50				
	HillSlope				
	EC50				
	amount at 10-6M				
**Plate #'s**		**4b-hydroxystanozolol**	**9a-fluoro-17a-methyl-androst-4-ene-a,6b,1**	**17a-Ethynyl-testosterone**	**5b-androst-1en-17b-ol-3-one**
AQ897	Bottom				
	Top				
	LogEC50				
	HillSlope				
	EC50				
	amount at 10-6M				
**Plate #'s**		**4b-hydroxystanozolol**	**9a-fluoro-17a-methyl-androst-4-ene-a,6b,1**	**17a-Ethynyl-testosterone**	**5b-androst-1en-17b-ol-3-one**
AQ1347	Bottom				
AQ1348	Top	-2.55%	160.96%	31.93%	18.93%
AQ1349	LogEC50				
AQ1350	HillSlope				
	EC50				
	amount at 10-6M	2.79%	0.15%	31.03%	16.22%
**Plate #'s**		**4b-hydroxystanozolol**	**9a-fluoro-17a-methyl-androst-4-ene-a,6b,1**	**17a-Ethynyl-testosterone**	**5b-androst-1en-17b-ol-3-one**
AQ1355	Bottom				
AQ1356	Top	611998.90%	8.76%	32.58%	20.51%
AQ1357	LogEC50				
AQ1358	HillSlope				
	EC50				
	amount at 10-6M	3.29%	3.10%	29.14%	13.32%

Plate #'s		4b-hydroxystanozolol	9a-fluoro-17a-methyl-androst-4-ene-a,6b,1	17a-Ethynyl-testosterone	5b-androst-1en-17b-ol-3-one
AQ1360	Bottom				
AQ1361	Top	0.27%	0.72%	37.82%	103.80%
AQ1362	LogEC50				
AQ1363	HillSlope				
	EC50				
	amount at 10-6M	0.51%	0.52%	39.04%	8.25%
**Plate #'s**		**4b-hydroxystanozolol**	**9a-fluoro-17a-methyl-androst-4-ene-a,6b,1**	**17a-Ethynyl-testosterone**	**5b-androst-1en-17b-ol-3-one**
AQ301	Bottom				
	Top				
	LogEC50				
	HillSlope				
	EC50				
	amount at 10-6M				
**Plate #'s**		**4b-hydroxystanozolol**	**9a-fluoro-17a-methyl-androst-4-ene-a,6b,1**	**17a-Ethynyl-testosterone**	**5b-androst-1en-17b-ol-3-one**
AQ312	Bottom				
	Top				
	LogEC50				
	HillSlope				
	EC50				
	amount at 10-6M				
**Plate #'s**		**4b-hydroxystanozolol**	**9a-fluoro-17a-methyl-androst-4-ene-a,6b,1**	**17a-Ethynyl-testosterone**	**5b-androst-1en-17b-ol-3-one**
AQ352	Bottom				
	Top				
	LogEC50				
	HillSlope				
	EC50				
	amount at 10-6M				
**Plate #'s**		**4b-hydroxystanozolol**	**9a-fluoro-17a-methyl-androst-4-ene-a,6b,1**	**17a-Ethynyl-testosterone**	**5b-androst-1en-17b-ol-3-one**
AQ273	Bottom				
	Top				
	LogEC50				
	HillSlope				
	EC50				
	amount at 10-6M				

Plate #'s		4b-hydroxystanozolol	9a-fluoro-17a-methyl-androst-4-ene-a,6b,1	17a-Ethynyl-testosterone	5b-androst-1en-17b-ol-3-one
AQ327	Bottom				
	Top				
	LogEC50				
	HillSlope				
	EC50				
	amount at 10-6M				
**Plate #'s**		**4b-hydroxystanozolol**	**9a-fluoro-17a-methyl-androst-4-ene-a,6b,1**	**17a-Ethynyl-testosterone**	**5b-androst-1en-17b-ol-3-one**
AQ290	Bottom				
	Top				
	LogEC50				
	HillSlope				
	EC50				
	amount at 10-6M				
**Plate #'s**		**4b-hydroxystanozolol**	**9a-fluoro-17a-methyl-androst-4-ene-a,6b,1**	**17a-Ethynyl-testosterone**	**5b-androst-1en-17b-ol-3-one**
AQ711	Bottom				
	Top				
	LogEC50				
	HillSlope				
	EC50				
	amount at 10-6M				
**Plate #'s**		**4b-hydroxystanozolol**	**9a-fluoro-17a-methyl-androst-4-ene-a,6b,1**	**17a-Ethynyl-testosterone**	**5b-androst-1en-17b-ol-3-one**
AQ733	Bottom				
	Top				
	LogEC50				
	HillSlope				
	EC50				
	amount at 10-6M				
**Plate #'s**		**4b-hydroxystanozolol**	**9a-fluoro-17a-methyl-androst-4-ene-a,6b,1**	**17a-Ethynyl-testosterone**	**5b-androst-1en-17b-ol-3-one**
AQ735	Bottom				
	Top				
	LogEC50				
	HillSlope				
	EC50				
	amount at 10-6M				
